# Sub-Telomere Directed Gene Expression during Initiation of Invasive Aspergillosis

**DOI:** 10.1371/journal.ppat.1000154

**Published:** 2008-09-12

**Authors:** Andrew McDonagh, Natalie D. Fedorova, Jonathan Crabtree, Yan Yu, Stanley Kim, Dan Chen, Omar Loss, Timothy Cairns, Gustavo Goldman, Darius Armstrong-James, Ken Haynes, Hubertus Haas, Markus Schrettl, Gregory May, William C. Nierman, Elaine Bignell

**Affiliations:** 1 Department of Microbiology, Imperial College London, London, United Kingdom; 2 The J. Craig Venter Institute, Rockville, Maryland, United States of America; 3 Korea University, College of Medicine, Department of Medicine, Anam-Dong, Seongbuk-Gu, Seoul, Korea; 4 Faculdade de Ciências Farmacêuticas de Ribeirão Preto, Universidade de São Paulo, Brazil; 5 Biocenter-Divison of Molecular Biology, Innsbruck Medical University, Innsbruck, Austria; 6 Microbiology and Molecular Genetics, UT-Houston Medical School, Houston, Texas, United States of America; 7 The George Washington University School of Medicine, Department of Biochemistry and Molecular Biology, Washington D.C., United States of America; Johns Hopkins University School of Medicine, United States of America

## Abstract

*Aspergillus fumigatus* is a common mould whose spores are a component of the normal airborne flora. Immune dysfunction permits developmental growth of inhaled spores in the human lung causing aspergillosis, a significant threat to human health in the form of allergic, and life-threatening invasive infections. The success of *A. fumigatus* as a pathogen is unique among close phylogenetic relatives and is poorly characterised at the molecular level. Recent genome sequencing of several *Aspergillus* species provides an exceptional opportunity to analyse fungal virulence attributes within a genomic and evolutionary context. To identify genes preferentially expressed during adaptation to the mammalian host niche, we generated multiple gene expression profiles from minute samplings of *A. fumigatus* germlings during initiation of murine infection. They reveal a highly co-ordinated *A. fumigatus* gene expression programme, governing metabolic and physiological adaptation, which allows the organism to prosper within the mammalian niche. As functions of phylogenetic conservation and genetic locus, 28% and 30%, respectively, of the *A. fumigatus* subtelomeric and lineage-specific gene repertoires are induced relative to laboratory culture, and physically clustered genes including loci directing pseurotin, gliotoxin and siderophore biosyntheses are a prominent feature. Locationally biased *A. fumigatus* gene expression is not prompted by *in vitro* iron limitation, acid, alkaline, anaerobic or oxidative stress. However, subtelomeric gene expression is favoured following *ex vivo* neutrophil exposure and in comparative analyses of richly and poorly nourished laboratory cultured germlings. We found remarkable concordance between the *A. fumigatus* host-adaptation transcriptome and those resulting from *in vitro* iron depletion, alkaline shift, nitrogen starvation and loss of the methyltransferase LaeA. This first transcriptional snapshot of a fungal genome during initiation of mammalian infection provides the global perspective required to direct much-needed diagnostic and therapeutic strategies and reveals genome organisation and subtelomeric diversity as potential driving forces in the evolution of pathogenicity in the genus *Aspergillus*.

## Author Summary

Airborne spores of the fungus *Aspergillus fumigatus* are present in significant quantities worldwide and are responsible for a range of illnesses from allergy to deadly invasive lung infection. A number of fungal properties are likely required for germination and growth of the fungus in the host, and now that the genome sequence of *A. fumigatus* is available it is possible to address which genes become important during initiation of infection. Understanding this might lead to new therapeutics and diagnostic tools. We have compared *A. fumigatus* gene activation during infection in a murine model to that in a laboratory culture to identify fungal attributes preferentially employed during disease. Our analysis entailed measurement of activity from most of the >9000 *A. fumigatus* genes, identifying iron limitation, alkaline stress, and nitrogen starvation as prominent stresses imposed by the host environment. We also found that genes preferentially employed for infection occur in clusters and are more likely to reside near the end of chromosomes, otherwise known as telomeres.

## Introduction

A small fraction of the estimated 1.5 million fungal species on Earth can colonise and infect human beings. Among them, the ascomycete *Aspergillus fumigatus* is the leading cause of mould-related death, most of which results from invasive lung disease in immune-deficient patients[1]. The ascomycetes' ecologically important saprophytism demands metabolic diversity and species-specific inventories of secreted enzymes. These are attributes which may ultimately contribute to the pathogenicity of certain species in plants and humans and have long influenced interpretations of virulence[1],[2]. Despite the importance of appropriate transcriptional control in orchestrating these processes, accurate data from within the host niche has eluded researchers, principally due to difficulties associated with sample recovery.

Most *A. fumigatus* infections are a direct consequence of the enormous propensity of *A. fumigatus* spores for airborne dispersal in large quantities, such that the human lung is constantly exposed to them. If infection ensues, its nature and severity is governed by the status of the host, which determines whether the spores are cleared effectively or whether they go on to germinate in, colonise, or even to invade the surrounding lung tissue[3]. *Ex vivo* and epidemiological analyses place macrophages and neutrophils on the frontline of cell-mediated defense[4],[5] against *A. fumigatus* infection and have implicated the fungal secondary metabolite gliotoxin (GT), in an immunotoxigenic capacity, as contributing to virulence[6]. This hypothesis was recently substantiated within a physiological context with the finding that the proapoptotic Bcl-2 family member, Bak, is required for GT-induced apoptosis in murine embryonic fibroblasts. Moreover, Bak knockout mice are resistant to *A. fumigatus* infection with a GT-producing clinical isolate[7]. Deletion of gliotoxin biosynthetic genes can differentially affect virulence, dependent upon immunsuppressive regimen, in murine infection supporting an important role of the host environment in determining pathogenic potential of *A. fumigatus*. This dichotomy of virulence phenotype renders the clinical significance of gliotoxin uncertain at the current time[8]–[12].

Fungi that sporulate or produce fruiting bodies demonstrate co-ordinate expression of biologically active secondary metabolites and spore-related products during development[13]. Such regulation is mediated at the level of transcription, from clusters of physically linked, co-ordinately regulated genes and is profoundly affected by both the developmental program under execution, and environmental factors such as pH and nutrient availability[14]. Renewed interest in the significance of secondary metabolites in establishing *Aspergillus* infection has been triggered by the discovery of a global transcriptional regulator of *A. fumigatus* secondary metabolite biosynthesis, LaeA[15]. *lae*A deletion does not affect gross changes in growth or sporulation in media *in vitro*, but it does result in reduced virulence in mice. This impairment in the ability to cause infection can be correlated with a loss of detectable gliotoxin as well as mis-regulation of gene expression within 13 gene clusters[16]. Siderophore-mediated iron uptake and storage, also partially under LaeA control[16] is indispensable for *A. fumigatus* virulence[17],[18], and adequate nutrient acquisition within the host niche is inextricably linked to pathogenesis of invasive disease being necessary to support sufficient growth to promote infection[19]–[21].

Transcriptional profiling can greatly illuminate the host pathogen interaction but the potential of this approach remains limited due to difficulties associated with obtaining good quality RNA in sufficient quantities from sites of infection. The feasibility of performing microarray analyses on limited material has been tested by a number of researchers employing linear amplification of mRNA, an approach which has recently proven successful in murine candidiasis[22], though a truly global transcriptional signature has yet to be reported for any fungal pathogen initiating mammalian invasive disease. The Eberwine method of mRNA amplification involves reverse transcription of mRNA with an oligo dT primer bearing a T7 RNA polymerase promoter site, to direct *in vitro* transcription of antisense RNA (aRNA) after double stranded cDNA synthesis and is favoured for linear mRNA amplification from limited quantities of starting material. It provides the basis for the methodology employed in our study, and in the majority of reported instances where mRNA amplification has been applied to samples destined for microarray analysis[23].

To identify fungal attributes preferentially employed during adaptation to the host niche, and thus contributing to the virulence of the saprophytic parasite *A. fumigatus*, we compared the transcriptomes of developmentally matched *A. fumigatus* isolates following laboratory culture or initiation of infection in the neutropenic murine lung. We report the development of a highly robust methodology for global profiling of *A. fumigatus* gene expression in germlings rescued directly from the murine lung, a tool which will empower the analysis of virulence in this pathogen. Our methodology employs facile molecular manipulations which, combined with custom bioinformatic scripts based on the latest annotations of the *A. fumigatus* Af293 genome, mark a significant advance in understanding orchestration of fungal virulence. Our analyses identify iron limitation, alkaline stress and nutrient deprivation as relevant host-imposed stresses during early-stage *A. fumigatus* infection and reveal a biased distribution of host-adaptation genes (relative to laboratory culture) in subtelomeric regions of chromosomes. Finally we assess lineage specificity of functions favoured during initiation of infection within varyingly virulent members of the genus *Aspergillus*.

## Results

### *A. fumigatus* RNA extraction and mRNA amplification

Microarray analyses are constrained by the availability of sufficient RNA for fluorophore labelling and hybridisation. One strategy to overcome the requirement for large quantities of material is global amplification of the sample. This approach has been employed for a number of reported microarray expression analyses[23],[24] with favourable results. We chose to analyse an early time point of infection to facilitate separation of fungal cells from those of the host with the advantage of studying a stage at which germinating hyphae have sensed, and are adapting to, their host. Early time points of *A. fumigatus* infection represent a vulnerable phase of morphogenesis *in vivo* since epithelial invasion and formation of mycelial mass have yet to occur. They also mark a point at which diagnoses capable of distinguishing infection from carriage of fungal spores would be most desirable, and antifungal therapy most effective.

**Figure 1:**
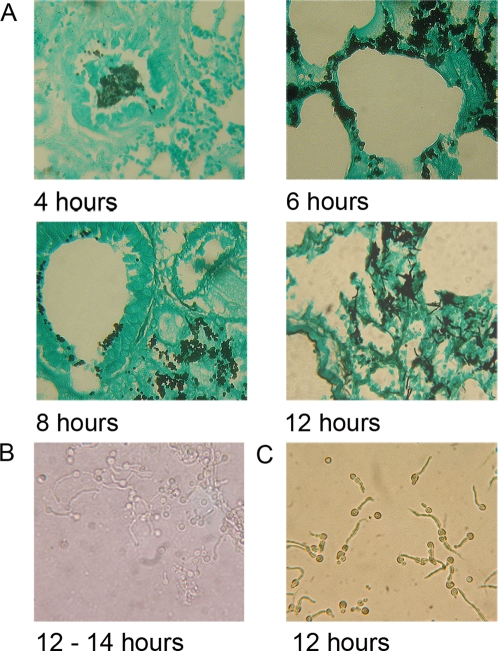
Comparative time-course of *A. fumigatus* Af293 germination and hyphal development in the murine lung, and laboratory culture. (A) Time-course of Af293 germination and hyphal development in the neutropenic murine lung. (B) Microscopic appearance of Af293 germlings recovered from a typical single murine BALF, (harvested at 12–14 hours post-infection). (C) Microscopy of developmentally matched laboratory cultured Af293 germlings, following liquid culture for 12 hours in YPD at 37°C.

We firstly characterised the time course of hyphal development in the sequenced clinical isolate Af293 by histopathological examination of infected neutropenic murine lung tissues (Figure 1). Lung sections collected and formalin-fixed at 4, 6, 8 and 12 hours post-infection contained numerous *A. fumigatus* spores in close association with murine epithelium in the bronchioles and alveoli (Figure 1A). At 12–14 hours post-infection >80% of *A. fumigatus* conidia had undergone germination and primary hyphal production. We therefore performed all BAL extractions for downstream analyses on concurrently infected neutropenic mice within a two hour window of infectious growth corresponding to 12–14 hours post-infection. At this time point recovery of germlings in BAL fluid was routinely achievable in the order of 10^3^ germlings per lavaged lung (Figure 1B).

To isolate fungal RNA from the site of *A. fumigatus* infection we inoculated pools of 24 neutropenic CD1 male mice with 10^8^ conidiospores and culled after 12–14 hours. Bronchoalveolar lavage was performed immediately using pre-warmed sterile saline and samples (BALFs) were snap frozen prior to RNA extraction and amplification. Within infection groups BALFs were pooled prior to RNA extraction and mRNA amplification. Total RNA yields from pooled BALFs ranged from 108–800 ng and yielded up to 258 µg aRNA after 2 rounds of linear amplification. Amplification factors therefore ranged from 2.8×10^3^–3.9×10^5^ based upon 2% of the total RNA population being mRNA (Table S1). *In vitro* reference RNA samples were similarly prepared from developmentally matched *A. fumigatus* germlings (Figure 1C) which were harvested, and snap-frozen, following 12 hour culture at 37°C in rich medium and subjected to two rounds of mRNA amplification prior to co-hybridisation (Table S1).

### Impact of mRNA amplification on preservation of transcript ratios

**Figure 2:**
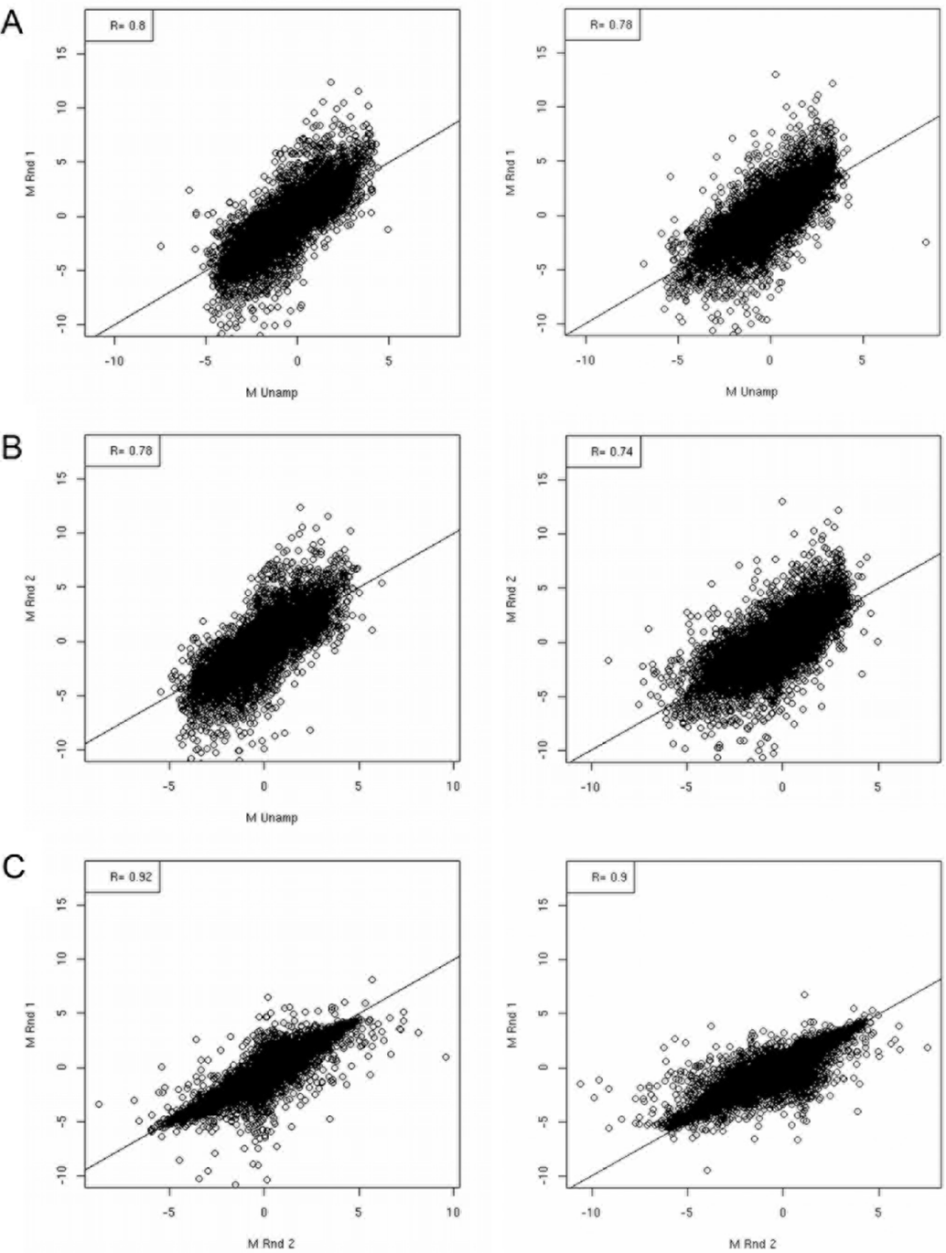
Correlation of log_2_ ratios resulting from comparative transcriptional analysis of the laboratory cultured *A. fumigatus* cell populations T0 and T60 under varying mRNA amplification protocols. Correlation of technically duplicated log_2_ ratios between competitive hybridisations using single (aRNA_1_), double (aRNA_2_) and unamplified (totRNA) RNA samples. (A and B) Correlation between log_2_ ratios obtained using cDNA derived from amplified and total RNA (totRNA v aRNA_r1_ *r* = 0.74–0.80, totRNA v aRNA_r2_ *r* = 0.74–0.80) (C) Cross-protocol pairings revealed highest correlations between slides using cDNA derived from amplified RNA (aRNA_r1_ v aRNA_r2_ *r* = 0.88–0.91) Surprisingly, technical replicates of slides using cDNA derived from total RNA (totRNA *r* = 0.80) were comparable to cross-protocol pairings (data not shown).

A suitable global mRNA amplification protocol should provide sufficient material for fluorophore cDNA labelling reactions whilst preserving the samples' original relative transcript abundance. To estimate amplification-related error (and distinguish such error from systematic error inherent to microarray methodologies) a mock experiment was devised to quantify the proportion of transcripts having significantly aberrant log_2_ ratios as a result of RNA amplification. This was achieved by indirectly comparing cDNA samples from different amplification protocols in a statistical linear model and fitting relevant contrast matrices (see Materials and Methods and Figure S1). For the mock experiment total RNA (totRNA) was isolated from two *A. fumigatus* cell populations T0 and T60, and subjected to either one (aRNA_r1_) or two (aRNA_r2_) rounds of mRNA amplification prior to cDNA fluorophore labelling and microarray hybridisation (Figure 2). To evaluate whether ratios are preserved between amplification protocols, we adopted the approach of Nygaard *et. al.* [23] who used corrected gene-wise t-tests to identify differences in the mean log intensity between aRNA_2_ and total RNA populations. In preparing the data for multiple t-testing, we excluded spots that were flagged by the the TIGR spotfinder software, or where the intensity was lower than twice the background intensity in either the Cy5 or Cy3 channel. The proportion of excluded spots was used as an indication of the hybridisation quality of the slide as a whole. Slides hybridised with aRNA had fewer spots (13.49–18.22%) removed by the filtering process than did slides hybridised with cDNA (44.56–48.95%), see Table S2. This apparent amplification-related improvement in hybridisation quality has been previously reported[24]. In our study, this can be attributed to a greater signal to noise ratio, and more specifically to increased foreground intensity on these slides (data not shown). We identified 8.49% of retained spots showing evidence of amplification protocol dependent differences in the log_2_ ratios. Thus our estimated measures of confidence from the above quality-control (QC) exercises fall within a range conducive for deriving biologically useful information, based upon reports of analytical studies published to date[23],[24] where Pearson correlation coefficients range from 0.75 to 0.99 (Figure 2) and rejected gene sets approximate 10% of spots included in QC analysis.

### *A. fumigatus* transcript profile during initiation of mammalian infection

**Figure 3:**
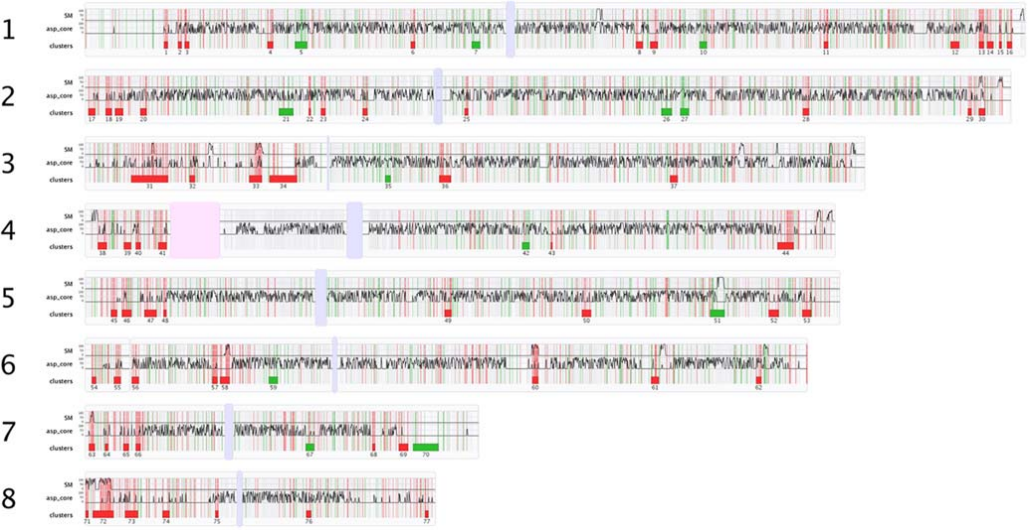
A genome-wide transcriptional snapshot of *A. fumigatus* Af293 during intiation of murine infection. Red and green vertical lines correspond to individual up- and down-regulated genes, respectively. Thin light gray vertical lines indicate the positions of all other genes. (SM) and (asp_core) are density graphs of secondary metabolite and *Aspergillus*-core genes, respectively, expressed as a percentage of the total bases contained per gene type, per non-overlapping 2 kb of chromosomal sequence. Induced and repressed gene clusters, are depicted by red and green rectangles, respectively, below each chromosome. A complete listing of genes housed in these co-regulated clusters can be found in Table S6. Light blue/gray vertical bars represent putative centromeres and the pink vertical bar in chromosome 4 represents a region of ribosomal DNA.

A common reference design was adopted for the microarray experiment from which data were analysed and processed as described in the Materials and Methods section. We performed the infection experiment 5 times in total generating pooled (n = 24) BALFs from five independent Af293 infections (samples A to E, Table S1). These samples were co-hybridised with similarly amplified mRNA prepared from developmentally matched laboratory cultured Af293 germlings (Materials and Methods and Table S1). Fluorescent signals from 9075 out of a possible 9516 represented ORFs were detectable from these hybridisation analyses (Dataset S1 and Array Express (http://www.ebi.ac.uk/microarray-as/aer/#ae-main0 Accession number E-TABM-327). Of 2180 genes (22.6% of the whole genome) having a fold-change in log intensity ratio of 2 or greater, 1281 were up-regulated and 897 were down-regulated. The entire expression dataset is graphically represented in Figure 3 which plots log_2_ ratios against chromosomal locus. Initial interpretations of the dataset were performed by the Expression Analysis Systematic Explorer[25] (EASE) to infer function by homology to *Saccharomyces cerevisiae* and identify over-represented Gene Ontology (GO) terms among differentially expressed genes. The results of these analyses are partially listed in Table 1 (full listings in Table S3). Distinct trends among favoured cellular processes are evident from these analyses which reveal a marked investment, by the host-adapting *A. fumigatus* cell, into transport of metal ion, cation, carbohydrate and siderophore iron. Given the importance of iron acquisition for microbial pathogenesis in general, and the absolute requirement for siderophore biosynthesis during murine *A. fumigatus* pathogenesis in this model of infection [17],[18] we expected transcripts from genes involved in iron mobilization and transport to be differentially abundant in this analysis, relative to iron-rich laboratory culture media. Accordingly we could identify a minimum of eleven siderophore biosynthesis/transport genes as important during growth in the murine lung (Table 2) including two ferric-chelate reductases (Afu1g17270 and Afu6g13750). Thirteen amino acid permease genes were more abundantly represented during host-adaptation than growth in YPD (Table 2) including 4 GABA (Afu8g01450, Afu7g0040, Afu5g14660 and Afu5g00710), and three proline permeases (Afu2g11220, Afu8g02200 and Afu7g01090) as well as the general amino acid permease, Gap1 (Afu7g04290). Nine genes annotated as maltose permeases or transporters in the current Af293 annotation were also more abundantly represented during initiation of murine infection (Table 2). Extracellular proteases have been implicated as virulence factors in invasive aspergillosis, as well as antigens causing inflammatory irregularity during allergic *A. fumigatus* disease[1]. Our analysis identified increased abundance of transcripts from the elastinolytic metalloprotease (Mep) (Afu8g07080), an aorsin-like serine protease (Afu6g6g10250) and three dipeptidylpeptidases (Afu4g09320, Afu2g09030 and AfuAfu3g07850). Thus transcription of this subset of *A fumigatus* proteases is significantly higher in the murine lung relative to rich laboratory culture. Functional categories of ergosterol biosynthesis, heme biosynthesis and aerobic respiration were significant among genes underrepresented during infection, relative to laboratory culture (Table 1) as well as multiple functional categories representing ribosome biogenesis and assembly, and protein biosynthesis and processing. This may reflect the poor nutritional value of murine lung relative to YPD and/or reduced growth (due to any number of stresses) during host-adaptation compared to broth culture. This trend is evidenced on multiple levels within our dataset, comprising repression of genes directing ribosomal protein synthesis, rRNA synthesis, RNA polymerase I and II activity, translation initiation and elongation, tRNA processing and synthesis, intracellular trafficking, secretion and vesicular trafficking (Table 1 and Table S3). While such metabolic dampening is often observed in microbial systems under stress, the observed *A. fumigatus* regulatory signature mimics that of rapamycin-mediated TOR kinase[26] inhibition and typifies fungal starvation. The *S. cerevisiae* TOR proteins *TOR1* and *TOR2* are phosphatidylinositol kinase homologues, first identified as the targets of the immunophilin-immunosuppressant complex FKBP-rapamycin[27], combined deletion of which causes yeast cells to arrest growth, undergo a reduction in protein synthesis, accumulate the storage carbohydrate glycogen and acquire thermotolerance. Comparison of our dataset to that obtained following rapamycin-induced TOR inhibition in *S. cerevisiae* [26] reveals extensive overlap in induced (n = 35) and repressed (n = 90) homologous genes between the two datasets (Table S4). Thus a clear TOR repression-like starvation signature, relative to laboratory culture, is observable during early-stage infection, which may derive from the relative nutritional status of the tested conditions and/or slower growth within the context of our experiment. The indicated cellular down-turn in metabolism observed is strongly countered by up-regulation of genes encoding functions associated with amino acid and carbohydrate catabolism (Tables 1 and S3).

**Table 1:** Over-represented Gene Ontology terms among differentially expressed genes.

Over represented biological processes among genes induced *in vivo*						
GO ID	GO term	List hits	List size	Pop.hits	Pop. Size	Fisher's Exact
GO:0006810	transport	133	448	761	4219	1.08E-10
GO:0008643	carbohydrate transport	19	448	40	4219	3.41E-09
GO:0009063	amino acid catabolism	14	448	35	4219	5.37E-06
GO:0044242	cellular lipid catabolism	10	448	19	4219	6.30E-06
GO:0044270	nitrogen compound catabolism	15	448	42	4219	1.28E-05
GO:0030001	metal ion transport	19	448	74	4219	1.76E-04
GO:0015892	siderophore-iron transport	5	448	8	4219	5.63E-04
GO:0006812	cation transport	21	448	94	4219	6.45E-04
GO:0019541	propionate metabolism	3	448	4	4219	4.38E-03
GO:0006830	high-affinity zinc ion transport	2	448	2	4219	1.13E-02
GO:0006631	fatty acid metabolism	13	448	62	4219	1.17E-02

Over represented biological processes among genes repressed *in vivo*						
GO ID	GO term	List hits	List size	Pop.hits	Pop. Size	Fisher's Exact
GO:0019538	protein metabolism	174	561	781	4219	7.35E-15
GO:0042254	ribosome biogenesis and assembly	51	561	161	4219	5.56E-10
GO:0044237	cellular metabolism	366	561	2251	4219	6.51E-10
GO:0007046	ribosome biogenesis	46	561	140	4219	1.08E-09
GO:0016072	rRNA metabolism	41	561	118	4219	1.37E-09
GO:0006364	rRNA processing	40	561	115	4219	2.12E-09
GO:0044238	primary metabolism	334	561	2028	4219	3.30E-09
GO:0006457	protein folding	23	561	50	4219	1.48E-08
GO:0006412	protein biosynthesis	72	561	302	4219	1.63E-07
GO:0006783	heme biosynthesis	6	561	13	4219	4.03E-03
GO:0009060	aerobic respiration	13	561	48	4219	8.02E-03
GO:0006696	ergosterol biosynthesis	11	561	38	4219	8.45E-03

Table lists selected biological processes significantly over-represented among differentially expressed genes, with respect to their occurrence in the *A. fumigatus* Af293 genome. To identify over-represented Gene Ontology terms, loci having significantly different expression were analyzed by the Expression Analysis Systematic Explorer (EASE) (PMID:14519205), which is implemented in MEV within the TIGR TM4 microarray data analysis suite (http://TM4.org). Numbers of genes in the indicated Gene Ontology categories were subjected to statistical analysis by EASE[25] to identify categories overrepresented compared with the whole genome data set. Only categories with Fisher's exact test probabilities below 5.00E-02 were included. Full results of the analysis can be found in Table S3.

**Table 2:** Functional classification of selected genes having altered transcript abundance, relative to laboratory culture, in the murine lung.

Ergosterol and Heme Biosynthesis			Iron acquisition		
Log_2_ Ratio	ORF Number	Gene annotation	Log_2_ Ratio	ORF number	Gene annotation
−2.072748506	Afu1g05720	c-14 sterol reductase	2.3932159	Afu7g06060	siderochrome-iron transporter (Sit1)
−2.135790025	Afu1g03950	cytochrome P450 sterol C-22 desaturase	2.5525794	Afu7g04730	siderochrome-iron transporter
−2.1829312	Afu8g07210	hydroxymethylglutaryl-CoA synthase	3.9717973	Afu6g13750	ferric-chelate reductase
−2.297904455	Afu5g02450	farnesyl-pyrophosphate synthetase	6.105128	Afu3g03440	MFS family siderophore transporter
−2.346162536	Afu7g03740	14-alpha sterol demethylase Cyp51B	3.4018665	Afu4g14640	low affinity iron transporter
−2.895486437	Afu1g07140	c-24(28) sterol reductase	2.6380832	Afu3g03650	sidG
−2.923648336	Afu1g03150	c-14 sterol reductase	6.4549049	Afu3g03640	siderochrome-iron transporter (MirB)
−2.970089164	Afu2g00320	sterol delta 5	6.1431709	Afu3g03420	sidD
−3.376200503	Afu6g05140	sterol delta 5	6.4520669	Afu3g03400	siderophore biosynthesis acetylase AceI (sidF)
−3.422246472	Afu5g14350	c-24(28) sterol reductase	3.4125222	Afu3g03390	siderophore biosynthesis lipase/esterase
−3.931510834	Afu4g06890	14-alpha sterol demethylase Cyp51A	4.8541282	Afu3g03350	nonribosomal peptide synthase (sidE)
−4.401905784	Afu4g09190	S-adenosyl-methionine-sterol-C- methyltransferase	4.0089025	Afu3g01360	siderochrome-iron transporter
−4.300123651	Afu1g07480	coproporphyrinogen III oxidase	5.2921431	Afu1g17270	ferric-chelate reductase (Fre2)
−2.435558859	Afu5g06270	5-aminolevulinic acid synthase	2.6644565	Afu1g17200	nonribosomal peptide synthase (sidC)
−2.128197751	Afu5g07750	ferrochelatase precursor	2.6144266	Afu8g01310	metalloreductase (FRE1)
−3.016960611	Afu6g07670	cytochrome c oxidase assembly protein cox15	**Carbohydrate transport**		
**Nitrate assimilation**			5.7013458	Afu7g06390	maltose permease
−4.050567754	Afu1g12840	nitrite reductase	5.8795989	Afu7g05190	maltose permease
−0.297138231	Afu1g12850	nitrate transporter (nitrate permease)	4.6926397	Afu6g11920	maltose permease
−2.332483829	Afu1g12830	nitrate reductase NiaD	2.722422	Afu5g00500	maltose permease
0.471365637	Afu5g10420	nitrate reductase	4.7493339	Afu3g01700	maltose permease
7.059699432	Afu1g17470	high affinity nitrate transporter NrtB	2.917548	Afu2g10910	maltose permease
0.55716766	Afu6g13230	Nit protein 2	2.6084555	Afu1g03280	maltose permease
**Secreted Proteins**			3.2031907	Afu3g03380	maltose O-acetyltransferase
7.40411592	Afu5g14190	beta-glucanase	2.5614034	Afu8g07070	maltase
5.898216645	Afu1g17510	lipase/esterase	4.678081	Afu7g06380	maltase
5.644563577	Afu2g09380	cutinase	3.4802952	Afu4g00150	MFS maltose transporter
5.496020587	Afu8g07090	extracellular proline-serine rich protein	2.2425193	Afu8g07240	MFS maltose permease
5.069338693	Afu2g05150	cell wall galactomannoprotein Mp2	3.0811489	Afu6g01860	MFS lactose permease
5.009338142	Afu5g00540	extracellular signaling protein FacC	2.0826028	Afu1g17310	MFS lactose permease
4.974193046	Afu7g01180	extracellular lipase	4.3502528	Afu3g01670	MFS hexose transporter
4.926922531	Afu1g16250	alpha-glucosidase B	4.6946836	Afu2g08120	MFS monosaccharide transporter (Hxt8)
4.701754948	Afu3g14030	alkaline phosphatase	3.3131505	Afu5g14540	MFS monosaccharide transporter
4.683929957	Afu2g00490	glycosyl hydrolase	4.8648019	Afu4g00800	MFS monosaccharide transporter
4.678081027	Afu7g06380	maltase	5.0594343	Afu7g00780	MFS monocarboxylate transporter
4.659742223	Afu8g01050	lipase/esterase	2.7697017	Afu3g03320	MFS monocarboxylate transporter
4.62947329	Afu3g14910	extracellular signalling protein (factor C)	3.0355716	Afu3g03240	MFS monocarboxylate transporter
4.628176816	Afu8g01130	alpha-galactosidase C	2.8084929	Afu6g03060	monosaccharide transporter
4.576610248	Afu4g01070	acid phosphatase	3.4709519	Afu5g01160	monosaccharide transporter
4.53846138	Afu7g05610	glucanase	3.7652613	Afu4g13080	monosaccharide transporter
4.316438927	Afu4g00870	antigenic cell wall galactomannoprotein	2.8100298	Afu7g05830	MFS sugar transporter
4.069715654	Afu6g02980	extracellular exo-polygalacturonase	6.9692415	Afu6g14500	MFS sugar transporter
3.883566446	Afu8g04710	xylosidase	2.0479063	Afu5g06720	MFS sugar transporter
2.176380223	Afu3g07850	dipeptidyl aminopeptidase Ste13	4.4698085	Afu1g11050	MFS sugar transporter
3.497276152	Afu2g09030	secreted dipeptidyl peptidase	2.4191786	Afu3g12010	high-affinity hexose transporter
3.821050502	Afu6g11500	dipeptidase	2.8865489	Afu3g00430	high-affinity glucose transporter
**Antigens**			3.2509899	Afu8g04480	hexose transporter protein
5.642295426	Afu4g09320	antigenic dipeptidyl-peptidase Dpp4	3.6472387	Afu6g06730	l-fucose permease
4.316438927	Afu4g00870	antigenic cell wall galactomannoprotein	**Amino acid transport**		
7.128239041	Afu4g09580	major allergen Asp F2	4.7164066	Afu7g04290	amino acid permease (Gap1)
6.439814589	Afu8g07080	elastinolytic metalloproteinase Mep	4.0522077	Afu2g08800	amino acid permease (Dip5)
3.218425558	Afu6g10250	alkaline serine protease AorO	3.7333962	Afu8g06090	amino acid permease
**Carbohydrate and/or protein glycosylation**			5.6472247	Afu5g09440	amino acid permease
2.540391388	Afu8g02020	glycosyl transferase	2.9622857	Afu2g10560	amino acid permease
6.098633033	Afu4g14070	glycosyl transferase	3.2688804	Afu1g09120	amino acid permease
2.229215652	Afu5g00670	glycosyl hydrolase family 35	2.9580145	Afu8g02200	proline permease
2.266930252	Afu6g11910	glycosyl hydrolase family 3	5.7069031	Afu7g01090	proline permease
3.177447564	Afu4g00390	glycosyl hydrolase	3.3818247	Afu2g11220	proline permease
2.065677868	Afu2g03270	glycosyl hydrolase	4.1850538	Afu8g01450	GABA permease
4.683929957	Afu2g00490	glycosyl hydrolase	2.3940135	Afu7g00440	GABA permease
2.14112188	Afu2g03120	cell wall glucanase (Utr2)	2.9136458	Afu5g14660	GABA permease
3.080401687	Afu8g05610	cell wall glucanase (Scw11)	6.28253	Afu5g00710	GABA permease
5.069338693	Afu2g05150	cell wall galactomannoprotein Mp2	6.7336819	Afu1g14700	allantoate transporter
**Carbohydrate catabolism**			**Metal ion transport/homeostasis**		
2.832926255	Afu6g14490	beta-glucosidase	4.9722279	Afu5g09360	calcineurin A
2.109492985	Afu3g00230	beta-glucosidase	5.7140897	Afu7g01030	Calcium-transporting ATPase 1 (PMC1)
7.40411592	Afu5g14190	beta-glucanase	3.30078	Afu3g10690	calcium-translocating P-type ATPase(PMCA-type)
3.165725818	Afu5g14550	beta-galactosidase	2.2461463	Afu3g08540	Ca2+ binding modulator protein (Alg2)
2.183493763	Afu1g14170	beta-galactosidase	2.7693449	Afu6g00470	plasma membrane zinc ion transporter
7.164853239	Afu7g06140	beta-D-glucoside glucohydrolase	4.2562039	Afu5g03550	plasma membrane H(+)ATPase
3.586205167	Afu6g08700	beta glucosidase	3.2581249	Afu1g02480	plasma membrane ATPase
3.25484732	Afu1g16700	beta galactosidase	4.1291169	Afu1g01550	high affinity zinc ion transporter
4.926922531	Afu1g16250	alpha-glucosidase B	3.103044	Afu8g01890	Na+/H+ exchanger family protein
3.825416885	Afu4g10150	alpha-glucosidase	3.3213401	Afu7g04570	Na/K ATPase alpha 1 subunit
4.628176816	Afu8g01130	alpha-galactosidase C	**Oxidative stress resistance**		
6.513162955	Afu1g01200	alpha-galactosidase	4.2704386	Afu1g14550	Mn superoxide dismutase MnSOD
2.86708433	Afu8g07300	alpha/beta hydrolase	−0.7096154	Afu4g11580	Mn superoxide dismutase (SodB)
4.46285962	Afu8g00570	alpha/beta hydrolase	2.3390424	Afu8g01670	bifunctional catalase-peroxidase Cat2
2.441133251	Afu8g00530	alpha/beta hydrolase			
2.041648909	Afu7g00830	alpha/beta hydrolase			
5.206583195	Afu3g01280	alpha/beta hydrolase			

Differentially regulated transcripts were defined as having log_2_(Cy5 – Cy3) greater than the arbitrary thresholds of plus and minus two.

### Lineage specificity and locational analyses of differentially expressed genes

**Figure 4:**
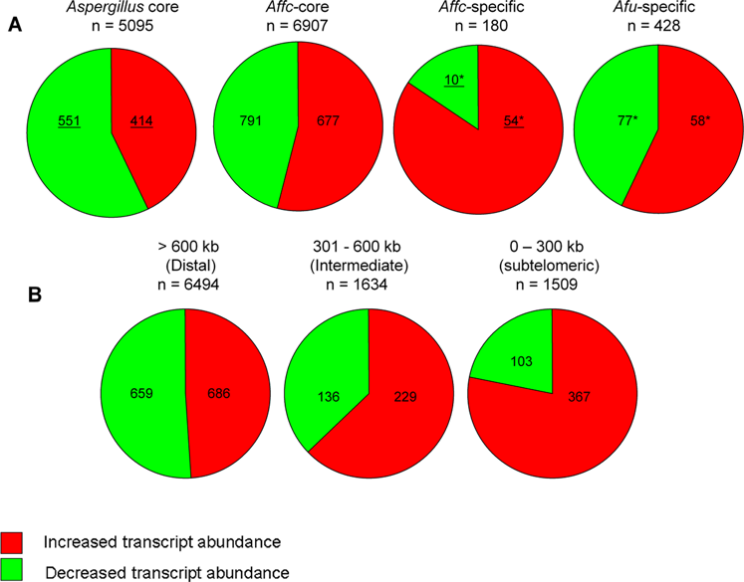
Distribution of lineage specific and telomere-proximal genes among differentially expressed host adaptation dataset. (A) Lineage specificity of *A. fumigatus* genes having altered transcript abundances, relative to laboratory culture, in the murine lung. The *Aspergillus*-core (Asp-core) set contains *A. fumigatus* Af293 proteins that have orthologues in *A. clavatus* (AAKD00000000), *N. fischeri* (AAKE00000000), *Aspergillus terreus* NIH2624 (AAJN01000000), *Aspergillus oryzae* RIB40[70], *A. nidulans* FGSC A4[71] and *Aspergillus niger* CBS 513.55[72] The Affc-core set were defined as *A. fumigatus* Af293 proteins that have ortholouges in *N. fischeri* and *A. clavatus*. The Affc-unique set is a sub-set of Affc-core proteins that do not have ortholouges in *A. terreus*, *A. oryzae*, *A. nidulans* or *A. niger*. Asterisks indicate gene sets which are listed in Table S5. Underlined values significantly deviate from the null hypothesis that an equal number of induced and repressed genes will occur in each cohort, as estimated by Chi-square analysis (Table 3). (B) Chromosomal distribution of *A. fumigatus* genes having altered transcript abundances, relative to laboratory culture, in the murine lung. Distances from telomeres (kb) are noted above pie charts. Asterisked gene sets are listed in Supplementary Table S5. Underlined values significantly deviate from the null hypothesis that an equal number of induced and repressed genes will occur in each cohort, as estimated by Chi-square analysis (Table 3).

From an evolutionary perspective, relative proportions of genes being over- and underrepresented in the analysis differed significantly within lineage-specific gene cohorts (Figure 4A and Table 3). Genes having increased transcript abundance during infection are significantly enriched (p<0.0001 by chi-square analysis, see Table 3) among differentially expressed genes (n = 64) unique to the *A. fumigatus* lineage. Thus 93.6% of *A. fumigatus* genes having orthologues restricted to two very closely related, but differentially virulent, species *Neosartorya fischeri* (anamorph of *Aspergillus fischerianus*) (AAKE00000000) and *Aspergillus clavatus* (AAKD00000000) are more abundantly represented during the initiation of infection (Table 3). In contrast only 8% of genes having orthologues in all six *Aspergillus* species sequenced to date (i.e. the *Aspergillus* ‘core’ genome) are more abundantly represented under these conditions (n = 5095). This invariable ‘core’ genome encodes many functions associated with information processing, central metabolism and cell growth, retention of which is most likely to be essential for cellular survival[28]. Narrowing the phylogenetic sampling to include only *A. fumigatus* and its relatives *N. fischeri* and *A. clavatus* distinguishes several subtelomeric ‘genomic islands’ upon which phenotypic variation between species, including differing pathogenicity, might depend[28]. Accordingly we found differentially expressed genes to be unevenly distributed amongst *A. fumigatus* chromosomes (Figure 4B and Table 3). Induced genes form a significantly increased proportion of differentially regulated functions in intermediate (p<0.001) and subtelomeric (p<0.001) regions of the chromosomes (Figure 4B and Table 3). While only 16% of the predicted *A. fumigatus* gene repertoire is housed within 300 kb of telomeres (classed as the subtelomeric gene repertoire in our analyses), 29% of transcripts having increased abundance, relative to laboratory culture, in the murine lung are located to such subtelomeric areas, compared to just 11% of down-regulated transcripts. Moreover, 28% of the entire subtelomeric gene repertoire is represented in the induced dataset compared to only 8% of subtelomeric genes represented among down-regulated functions (Table 3).

**Table 3:** Distribution of induced and repressed genes among lineage specificity cohorts and chromosomal locations.

	Number of genes	Differentially expressed	UP' (observed)	DOWN' (observed)	UP' (expected)	DOWN' (expected)	χ2	p
*Aspergillus* core	5095	965	414	551	482.5	482.5	19.45	<0.0001
Affc-core	6907	1468	791	677	734	734	8.853	0.0029
Affc-specific	180	64*	54	10	32	32	30.25	<0.0001
Afu-specific	428	135*	77	58	67.5	67.5	2.674	0.102

	Number of genes	Differentially expressed	UP' (observed)	DOWN' (observed)	UP' (expected)	DOWN' (expected)	χ2	p
0–300 kb	1326	470	367	103	235	235	148.289	<0.0001
301–600 kb	1504	365	229	136	182.5	182.5	23.696	<0.0001
>600	5779	1345	686	659	672.5	672.5	0.5	0.4795

Chi-square analysis (with one degree of freedom) was used to test the distribution of induced and repressed genes with respect to lineage specificity and sub-genomic locations, based upon the null hypothesis that equal numbers of induced and repressed genes occur in each cohort. Asterisked gene populations are listed in Supplementary Table S5.

### Clustering of induced genes

Regarding our *A. fumigatus* gene expression dataset as a function of chromosomal locus (Figure 3) we identified that many induced *A. fumigatus* genes are found in contiguous clusters. To investigate this further we generated a custom script to automate cluster identification which identified numerous genomic loci within which co-ordinate regulation of a minimum of 5 closely neighbouring genes can be observed (Figure 3 and Table S6). Co-ordinate expression of physically clustered genes is a prominent feature of the induced, but not repressed, gene set and we observe a large proportion (40%) of up-regulated physically clustered genes to reside within 300 kb of chromosome ends (Figure 3 and Table 4). The clusters are comprised of up to 34 co-ordinately expressed genes and include loci directing biosynthesis of siderophores (cluster 33) and two known secondary metabolites, pseurotin and gliotoxin (clusters 72 and 60, respectively). The gliotoxin biosynthetic cluster is not subtelomerically located being 700 kb from the telomere (as annotated by Perrin *et. al.* [16]). The pseurotin biosynthetic cluster, however, (as annotated by Maiya *et. al.* [29]) is contained within the fumitremorgen cluster (Afu8g00100–8g00720) at 100 kb from the telomere[16]. Pseurotin[29]–[31] is a neuritogenic, nematicidal quinone and gliotoxin an immunotoxin which supports *A. fumigatus* virulence in some murine models of invasive pulmonary aspergillosis[8]–[12]. We observed four other postulated, but uncharacterised, secondary metabolite gene clusters induced during early stage *A. fumigatus* infection, including a large proportion of genes on the left arm of chromosome 8, predicted to encode a fumitremorgen biosynthesis supercluster[32]. Thus, it would seem that selective expression of a subset of secondary metabolite loci facilitates initiation of mammalian infection. While gliotoxin biosynthesis is dispensable for virulence in some murine models, our analysis demonstrates that this host environment is nonetheless conducive to immunotoxin production, and further insights on virulence mechanisms relevant to neutropenia and/or corticosteroid therapy await comparative analyses of fungal gene expression in each of these strikingly different host settings, an analysis which is currently underway in our laboratory. A fully annotated table of clusters, indicating between-species synteny within cluster loci, and all accession numbers, can be viewed in Dataset S2.

**Table 4:** Comparative analysis of genetic distribution among genes having differential transcript abundances in microarray analyses using *A. fumigatus* RNA following exposure to *in vitro* or murine-adaptive stress, or *lae*A gene deletion.

Experimental condition	Total genes in dataset	Proximal	%	Intermediate	%	Distal	%	*A. fumigatus*-specific	%
Genome stats	9632	1509	16	1634	17	6494	67	2201	23
Clustered *in vivo* up	658	227	34	129	20	302	46	218	33
*ΔlaeA* up	415	102	32	59	14	254	61	122	29
*in vivo* up	1282	367	29	229	18	686	54	374	30
Nitrogen starvation up	626	180	29	115	18	331	53	33	33
*in vivo* up (no sec mets)	1196	327	27	223	19	646	54	355	30
Neutrophils 60 minutes up	312	83	27	81	26	148	47	80	26
Alkaline adaptation up	211	53	25	39	18	113	54	6	6
*ΔlaeA* up (no sec mets)	318	70	22	58	18	190	60	50	16
Acid stress up	83	18	22	21	25	44	53	43	52
Iron limitation up	28	4	14	4	15	20	71	8	30
*ΔlaeA* down	528	93	18	99	19	336	64	108	20
Oxidative stress up	54	9	17	9	17	36	67	22	54
*in vivo* down	898	103	11	136	15	659	73	126	14

Gene density in telomere-proximal (o-300 kb from telomeres), intermediate (300–600 kb from telomeres) and telomere-distal (>600 kb from telomeres) regions of the *A. fumigatus* genome are indicated (Genome stats). (no sec mets) indicates omission of secondary metabolite biosynthetic genes (identified as detailed in the Materials and Methods section) from the tested dataset.

### Comparative analyses of murine adaptation- and *in vitro* gene expression datasets

**Figure 5:**
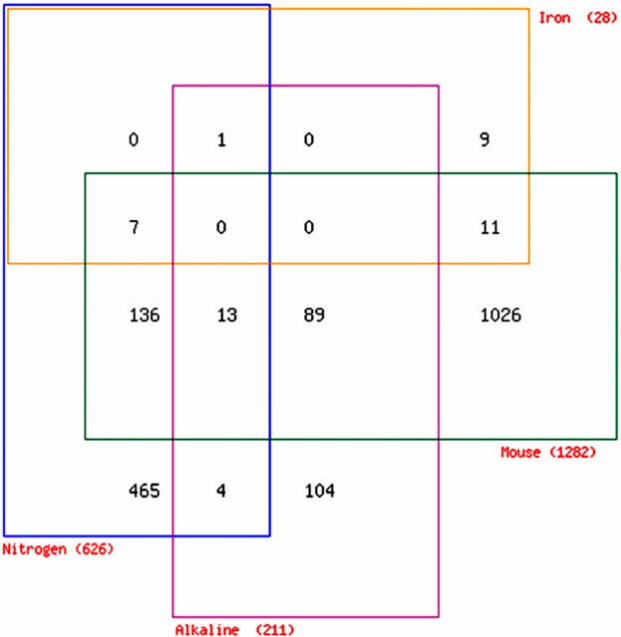
Overlap between murine adaptation and *in vitro* stress datasets. Venn diagrammatic representation of overlap between murine adaptation dataset and those of nitrogen starvation, iron starvation and alkaline shift. Genes are listed in Dataset S3.

**Figure 6:**
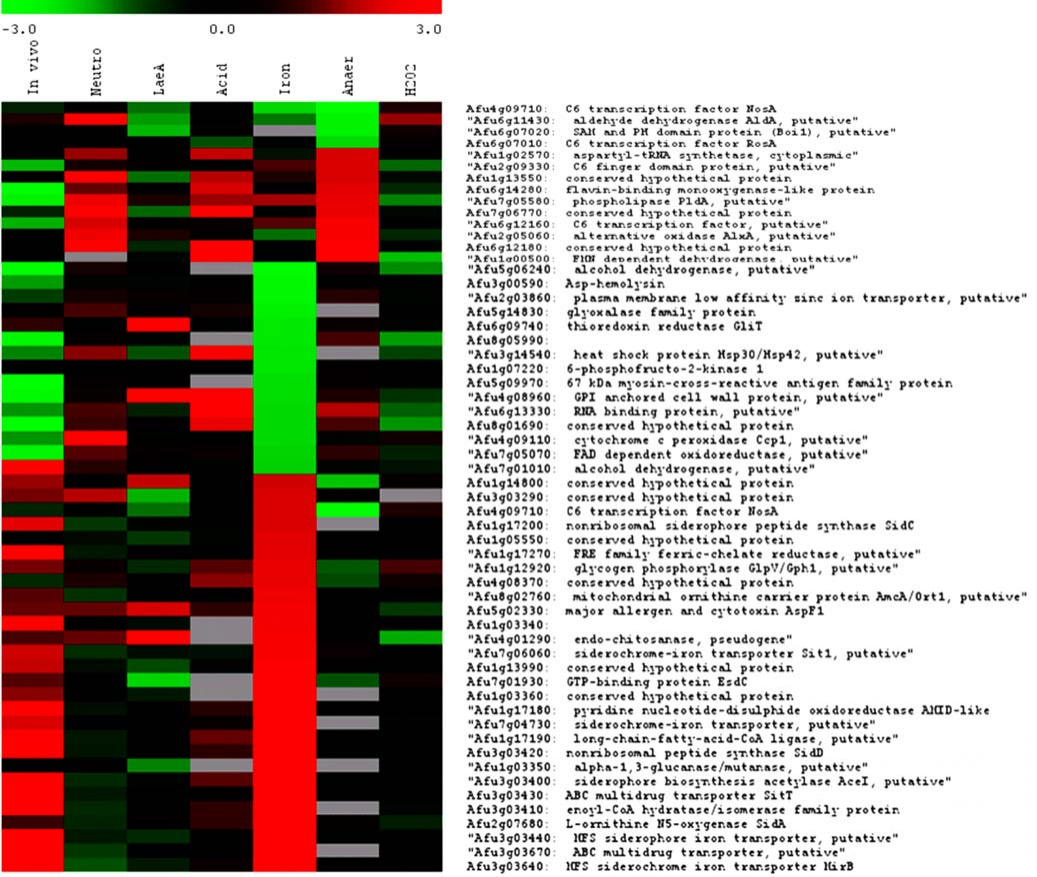
Comparative analysis of *A. fumigatus* gene expression datsets. A pan-experimental comparison of *A. fumigatus* gene expression aligning log_2_ ratios obtained during host adaptation (mice); exposure to neutrophils (neut), increased expression in parental strain versus Δ*lae*A mutant, acid shift (acid), iron starvation (iron), oxygen depletion (anaer) and oxidative stress (H_2_O_2_) for various genes. The colour bar indicates the range of log_2_ expression ratios, grey bars indicate genes from which signals were undetectable for technical reasons. Experimental conditions are described in Materials and Methods. LaeA dataset is taken from Perrin *et. al.* [16]. Comparative analyses were implemented in TM4 http://www.jcvi.org/cms/research/software/.

The complexity of the transcriptional signature derived from host adaptation analyses is likely to originate from convergence of multiple environmental cues, coupled with metabolic and morphologic effects. Although functional categorisation of differentially expressed functions (Tables 1 and S3) could identify metabolic and physiological trends during initiation of infection, environment-related signatures were less easy to discern. To ascertain physiologically relevant features of the host environment we compared the transcriptome of host-adapting germlings to those of *in vitro A. fumigatus* cultures exposed to iron limitation, nutrient limitation, alkaline stress, acid stress, neutrophils, oxidative stress or anaerobic stress. The resulting transcriptomic responses varied in magnitude (Table 4) but assignment of cut-off log_2_ ratio values of +2 and −2 across all of the analyses enabled us to distinguish several important aspects of the *A. fumigatus* host-adaptive response (Figure 5). The transcriptional signatures of paramount importance among those examined were alkaline adaptation, iron deprivation and nutrient starvation, which are remarkably prominent in the infection dataset. Using the aforementioned log_2_ cut-offs, 24 of 43 iron-regulated genes were identified as differentially expressed during host adaptation, of these 18 were more abundantly represented (Figure 5 and Dataset S3), and 6 less abundantly represented. Relaxing the cut-off criterion to encompass all differentially regulated genes in the host adaptation dataset allowed complete capture of the iron regulon (Figure 6). Among these genes are the siderophore biosynthetic genes *sid*A (Afu2g07680), *sid*D (Afu3g03420) and *sid*C (Afu1g17200), the latter two discerning biosynthesis of both intra- and extracellular siderophores during infection as substantiated by previous findings[18], and four siderochrome/siderophore transporter proteins Afu7g06060, Afu7g04730, Afu3g03440 and afu3g03640. Preferential gene expression following a 60 minute shift from acid to alkaline medium could also be strongly correlated with that observed during infection (Figure 5 and Dataset S3). Alkaline adaptive capability, previously found to be essential for *A. nidulans* virulence in neutropenic mice[33], is likely to be important for growth of *A. fumigatus* spores at physiological pH. Accordingly we identified 102 genes preferentially expressed during both murine infection and *in vitro* alkaline adaptation (Figure 5 and Dataset S3). Among them are 36 genes having unknown function, two sodium ATPases (Afu6g03690 and Afu4g09440), the plasma membrane zinc ion transporter (Afu6g00470) and an alkaline phosphatase (Afu3g14030). Interestingly, we found no concordance between iron starvation and alkaline adaptation (Figure 5). Since acidification of the macrophage phagolysosome is an essential step in ROS-mediated *A. fumigatus* killing we also assessed the transcriptome of Af293 germlings upon shift from pH7 to pH3, using a rich medium and hydrochloric acid. No concordance between the resulting dataset and that of host adaptation was evident, indeed (despite the magnitude of the murine infection dataset) most functions upregulated in response to an *in vitro* acid shift were less abundantly represented in our infection analyses (n = 18, Figure S2) thus we can confidently conclude that acid stress, at least within the context tested in this analysis (which can only approximate conditions encountered in the host) is not relevant during host adaptation. This agrees with our observation that alkaline adaptation is a physiological cue of primary importance in this murine model of infection (Figure 5).

An essential component of phagocyte defense against *A. fumigatus* conidia and hyphae is the NADPH oxidase-mediated respiratory burst which generates reactive oxygen species (ROS) required for fungal killing. To assess the *A. fumigatus* transcriptional response to oxidative stress, conidia were grown in rich medium at 37°C prior to shift into similar medium containing 17 mM hydrogen peroxide. Comparison of the resulting dataset to that obtained from host-adapting germlings revealed some concordance, this time revealing a subset of genes having decreased transcript abundance both *in vitro* and during murine infection (Figure S2). A common theme among this group of genes is ergosterol and heme biosynthesis, evidenced by common behaviour of both 14-alpha sterol demethylase Cyp51A-encoding genes (Afu4g06890 and Afu7g03740) and a coproporphyrinogen III oxidase homologue (Afu1g07480). Oxygen depletion was achieved by transfer of *A. fumigatus* hyphae, following 16 hour growth in rich medium, to anaerobic chambers containing two palladium catalysts. We were unable to correlate gene expression under these anaerobic stress conditions with gene expression during infection (Figure 6). Finally, we assessed germlings grown in 20 ml of RPMI1640 with L-glutamine, 25 mM HEPES and 5% fetal bovine serum for 7 hours at 37°C following exposure to human neutrophils at a multiplicity of infection of 1∶1 for 60 minutes, the reference sample for this analysis being germlings incubated in the absence of neutrophils. Of 57 *A. fumigatus* genes upregulated in response to neutrophil exposure *in vitro*, 18 (60%) were also more abundantly represented during initiation of murine infection (Figures 6 and S2). Interestingly these included the two major *A. fumigatus* antioxidant enzymes, Mn superoxide dismutase (Afu1g14550) and the bifunctional catalase-peroxidase Cat2 (Afu8g01670). Whether representation of these transcipts among those differentially expressed in both murine and laboratory culture is indeed neutrophil-specific remains to be determined.

### Nutrient limitation is a relevant physiological cue during *A. fumigatus* initiation of murine infection

**Figure 7:**
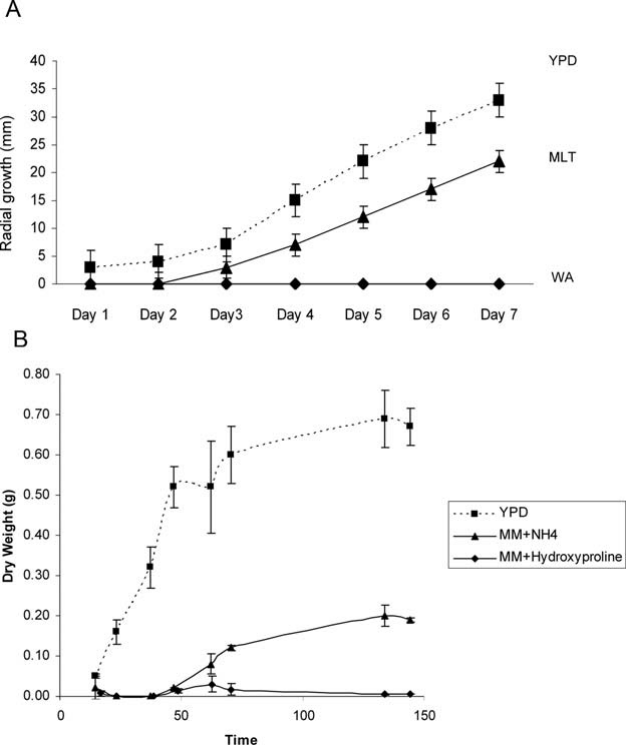
Characterisation of *A. fumigatus* growth, relative to YPD. (A) Comparative analysis of Af293 radial growth on YPD and synthetic murine lung tissue medium (MLT). Triplicated, spot-inoculated plates containing single 100 spore inocula were incubated at 37°C. (B) Growth curve analysis of Af293, performed in triplicate using liquid YPD, or AMM containing 1% glucose and either 5 mM ammonium tartrate or 5 mM hyroxyproline as nitrogen source. Cultures were inoculated to a final concentration of 5×10^6^ spores/ml and incubated under aerobic conditions at 37°C with shaking at 150 rpm. At selected timepoints mycelia were harvested on Miracloth, encased in Whatmann paper and dried at 37°C for 48 hours before weighing.

To investigate the relevance of nutrient starvation during host adaptation we made several growth and gene expression analyses. Hypothesising that YPD is nutritionally more robust than murine lung tissue we compared radial growth of *A. fumigatus*, in triplicate from an inoculum of 100 spores grown at an agar/air interface on Petri dishes, on YPD and on a synthesized murine lung tissue medium (MLT) composed of homogenised murine lung tissue (80%) and water/agar (20%), overlaid upon a water/agar baseplate. Growth of *A. fumigatus* was completely unsupported by water/agar base with no evidence of conidial germination after 7 days. Growth on YPD produced conidiating colonies averaging 66 mm (n = 3) in diameter after 7 days at 37°C, whereas MLT as growth medium supported significantly less growth, reaching a maximum colony diameter of 44 mm (Figure 7A). Any concern that reduced radial growth observed on MLT medium originates from iron deprivation can be allayed by reference to radial growth analysis of two independent wild type *A. fumigatus* isolates, CEA10 and ATTC46645[17], where equivalent growth is observed in the presence and absence of iron, and on blood agar medium. Thus, from this solid growth analysis, we conclude that MLT supports slower *A. fumigatus* colony growth than YPD under laboratory conditions. Our MLT analysis could not support the volume of liquid culture required to perform growth curve analyses, moreover, the viscosity of the medium would have hindered dry weight measurements. To support our conclusions on nutritive status of the host environment, within the context of the experimentation performed during our infection analyses, we assessed log_2_ ratios obtained from competitive microarray hybridisation, using doubly amplified *A. fumigatus* mRNA extracted from nitrogen starved germlings, and the same YPD reference sample used for the initial host adaptation analysis (samples F and G, respectively, Table S1). Nitrogen starvation was exerted in shaken liquid culture using minimal medium, with hydroxyproline as nitrogen source, for a period of five hours. Hydroxyproline is a rational candidate nitrogen source during initiation of mammalian pulmonary infection, being a widely used surrogate marker of lung injury whose concentration in bronchoalveolar lavage fluid permits quantitative assessment of collagen breakdown[34],[35]. Transcript levels of the proline permeases afu2g11220, Afu8g02200 and Afu7g01090 suggest induction of proline uptake during initiation of infection, and laboratory culture on solid medium confirms that hydroxyproline can support aconidial filamentous growth of *A. fumigatus* on minimal medium in the presence of a repressing carbon source such as glucose (data not shown). To confirm the inferiority of hydroxyproline as a nitrogen source (relative to YPD) we performed dry weight growth curve analyses (Figure 7B) including a widely used *Aspergillus* minimal medium (MM) for comparison.

Nitrogen starvation rendered 1047 genes subject to differential expression, relative to the doubly amplified YPD reference. Several notable features of the resultant dataset (Dataset S4) support our conclusion that, relative to YPD laboratory culture, initiation of murine infection occurs under nutrient stress. As with our analyses of host adaptation, over-represented Gene Ontology (GO) terms among differentially expressed genes identified ribosome biogenesis and assembly, and protein biosynthesis and processing as the most significantly down-regulated functional categories (Table S7). We also identified significant over-representation of cell cycle-related functions among genes preferentially expressed during growth in hydroxyproline, including cell cycle regulation, mitosis, nuclear migration, chromosome segregation and karyogamy (Table S7). This is a particularly satisfying finding since, supported by our growth curve analyses (Figure 7B), a clear distinction between *A. fumigatus* growth phase in the compared media is discernable. This was not a feature of the murine-YPD comparison which, importantly, lessens the probability that differences associated with cell cycle stage or growth rate preside over environmental cues within the host adaptation dataset. Direct comparisons of the murine and nitrogen starvation datasets identified an overlap of 280 differentially expressed genes common to both, of these 24 genes are also common to the TOR kinase nutrient limitation geneset, and are indicated in Table S4. 156 genes preferentially expressed under nitrogen starvation conditions (relative to YPD laboratory culture) were similarly favoured during host adaptation (Dataset S3). Assessing locational bias among the hydroxyproline dataset, 29% of the geneset was found to reside subtelomerically (Table 4) with 180 out of 634 preferentially expressed genes housed within 300 kb of telomeres. This is comparable to the level of subtelomeric gene expression identified during initiation of murine infection (Table 4) and indicates that, relative to growth in a nutrient-rich laboratory culture, adaptation to growth in a nutritionally challenging environment prompts expression of a subtelomeric gene repertoire. A number of physically linked coregulated genes came to prominence in the hydroxyproline starvation dataset (Table S8). 16 clusters conforming to the previously applied cluster algorithm were identified, 8 of which were subtelomeric and 3 of which encompass genes in secondary metabolite loci, as defined by bioinformatic analyses. Of the three latter loci one cluster (Afu6g03390–6g03490) subject to regulation by the LaeA methyltransferase[16], the product of which is currently unknown, is also expressed during murine infection. Beyond this, correlation between clustered gene regulation in murine and nitrogen starved growth was modest.

### The LaeA regulon is represented among genes preferentially expressed during intitation of murine infection

**Figure 8:**
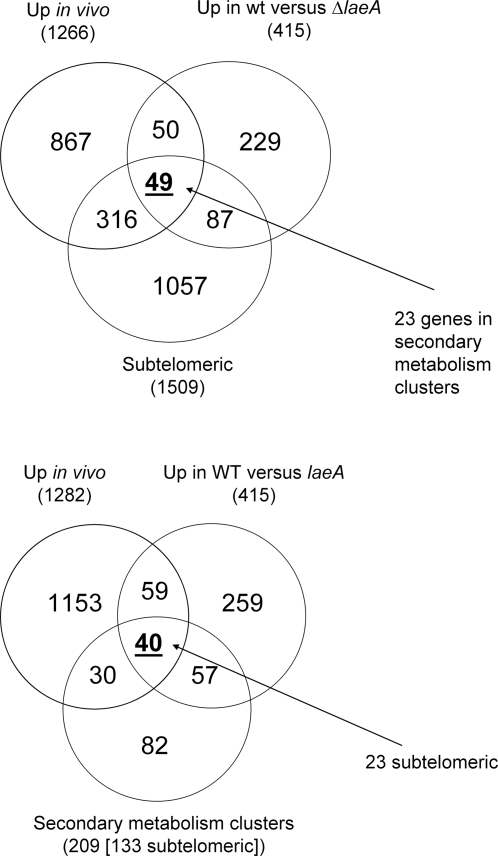
Expression of LaeA-regulated genes during initiation of murine infection. Venn diagram representation of overlap between genes repressed in ΔLaeA[16] and those having increased transcript abundance during murine infection, according to proportions having subtelomeric locations, and secondary metabolism functionality (on the basis of annotation).

Given the predominance of clustered and subtelomeric gene loci among host adaptation genes, we compared our dataset to that produced by Perrin *et. al.* [16] during study of *lae*A gene deletion. An *A. fumigatus* Δ*lae*A (1g14660) mutant, which has decreased virulence in both neutropenic and hydrocortisone treated mice[32],[36], demonstrates significantly lower expression of genes in 13 secondary metabolite biosynthetic clusters including that of gliotoxin. LaeA was found to influence expression of a subset of lineage- and species-specific genes therefore we tested the overlap between functions down-regulated in the absence of LaeA and functions having greater transcript abundance during murine infection, hoping to decipher a link between genes under LaeA control, and those active during initiation of murine infection. Out of 415 genes down-regulated in the absence of LaeA we identified 99 genes having increased abundance during initiation of murine infection (Figure 8). Functional categorisation of shared genes revealed that 40% (n = 40) were involved in secondary metabolite biosynthesis, among these we could identify three complete secondary metabolite clusters, those directing gliotoxin and pseurotin biosynthesis as well as the genetic locus mentioned above (Afu6g03390–6g03490) whose biosynthetic product is unknown. Perrin *et. al.* also found 54% of the LaeA-regulated gene clusters showing differential expression under laboratory conditions were located within 300 kb of the telomeres. We therefore extended our analysis still further, determining the proportions of subtelomeric and secondary metabolism cluster genes shared between the two datasets. This identified 49 and 40 genes, having subtelomeric locations and secondary metabolite biosynthetic functions respectively (Figure 8).

Finally we analysed all of the *in vitro* and *in vivo* datasets comparatively to assess the occurrence of subtelomeric bias among differentially expressed genes, aiming to determine whether the induction of genes at the telomeric extremities of chromosomes was a standard feature of adaptation to environmental alterations rather than a host adaptation phenomenon (Table 4). The analysis identified murine adaptation, *lae*A deletion, neutrophil exposure and nitrogen starvation as the conditions most enriching for transcript abundance among subtelomeric genes, where 29%, 32%, 27% and 29% of the respective cohorts were located. Interestingly, removal of secondary metabolism genes from the analysis dramatically reduced the LaeA regulated subtelomeric gene cohort to 22% while only minimally impacting representation of murine adaptation genes (Table 4).

Taken together these analyses indicate that a significant component of the LaeA regulon, comprised mainly of secondary metabolism genes, is represented among transcripts more abundant during infection. Furthermore the subtelomeric bias observed among differentially expressed murine adaptation genes extends beyond secondary metabolite biosynthesis and does not appear to be a general feature of adaptation to environmental change.

## Discussion

We present a methodology for *A. fumigatus* transcript profiling during initiation of murine infection and a comparative analysis of global transcriptional programming, in laboratory culture and the mammalian lung. We were able to optimize a technical and statistical framework sufficiently robust to reproducibly quantify relative transcript abundances using minute samplings of *A. fumigatus* germlings. The statistical analysis, though seemingly complex, utilizes standard statistical methods which are well documented in biological analyses, particularly in microarrays[37]. We chose to co-hybridize mock samples from the same amplification protocol (thereby determining the ‘true’ biological effect in a benchmark sample, in this case total RNA) and then, by comparison to cohybridisations using unamplified samples, indirectly estimate the systematic effect of the amplification in our analysis. The t-testing framework[23] then allowed us to identify genes where the log ratios are significantly different due to *one* factor i.e. the amplification protocol. This enabled an estimation of the proportion of genes showing amplification-dependent bias which we carried into the murine experiment, where comparison of unamplified versus amplified material is impossible. Clearly, we assume that this estimate can be reliably applied to similar experiments, in doing so we also assume that the amplification process depends only on the protocol adopted, and not the underlying gene expression dynamics. A further technical consideration in planning experiments comparing murine and laboratory samples is differential treatments. Given the nature of our analysis, differential treatment of the germling samples was necessary to avoid osmotic shock in either instance. Water lavage would impose an osmotic shift on germlings rescued from the lung and saline treatment would osmotically shock laboratory cultured germlings. Thus the technical limitations imposed by such comparative analysis must be accepted, however, in terms of maximally preserving transcript abundances within the context of the experiment, we believe our treatments of the samples to be appropriate.

To identify factors governing adaptation to the host niche we compared laboratory and murine lung samples aiming to find fungal attributes preferentially employed during infection. Therefore our findings document genes having increased transcript abundance in the *A. fumigatus* sample of murine origin, relative to that derived from laboratory cultured fungus. We performed our comparative analysis using doubly amplified mRNA from developmentally matched *A. fumigatus* germlings following laboratory culture, or growth in neutropenic murine lungs. In total we identified 2164 genes having altered transcript abundance. Functional analysis of the dataset flagged certain putatively relevant physiological cues which we then pursued by additional *in vitro* analyses.

Careful interpretation of the dataset with respect to specific nutrient acquisition mechanisms, and within the context of the comparison performed here, can lend powerful insight into accessible nutrients in the mammalian niche. Nitrate assimilation (which is strongly inducible by nitrate in the absence of preferred nitrogen sources ammonium and glutamine)[38] is not preferentially employed in the murine host as evidenced by co-ordinate down-regulation of *crn*A (Afu1g12850), *nia*D (Afu12830) and *nii*A (Afu1g12840). This may be due to a) equivalent nutritional status of YPD and murine lung or b) to the absence of nitrate as a nitrogen source during initiation of infection, or both. We were able to confirm, by radial growth analyses *in vitro* using YPD and a synthetic lung tissue medium MLT, that YPD is nutritionally superior to MLT based upon the rate of radial growth supported (Figure 7A). Coupled with the observation that the nitrogen metabolite repression gene product AreA is required for full virulence[19] a likely explanation for slowed growth and repression of nitrogen assimilation, is the utilisation of alternative non-preferred nitrogen sources during establishment of disease, such as amino acids. Strong support for this conclusion is provided by high level induction of the *are*A-dependent nitrogen-scavenging enzyme L-amino acid oxidase LaoA (Afu7g06810) which enables *Aspergilli* to catabolise a broad spectrum of amino acids in nitrogen starvation conditions[39]. Notably a by-product of such catabolism is ammonium. However the likliehood that sufficient ammonium is produced by these reactions to prevent starvation is diminished by the observed general starvation response in our dataset. Catabolism of amino acids during initiation of infection is also evidenced by induction of the methyl citrate synthase enzyme (Afu6g03590) which acts to detoxify the intermediates of propionyl-coA generating carbon sources[40], such as cysteine, isoleucine and methionine. An essential role for methylcitrate synthase in murine aspergillosis has recently been demonstrated[41] thereby demonstrating the value of our approach in generating physiological information on virulence mechanisms within the context of murine infection.

Strong themes among genes having lowered transcript abundance during murine infection are ribosome biogenesis and assembly, and protein bisoynthesis and folding (Table 1). Such signatures are commonly observed among microbes under stress, and in this instance might indicate a slowing of growth in the murine lung, relative to laboratory culture. We reasoned that a mechanism possibly linking such transcriptional profiles to nutrient starvation is TOR mediated ribosomal gene regulation, which tightly couples protein synthesis and cellular growth to availability of nutrients and physiological status to balance the opposing forces of protein synthesis and degradation. This is pivotal for cellular fate determination in many organisms[42],[43], propelling cells towards *either* proliferation (through the cell cycle) or vegetative growth (increase in size). Comparison of our dataset to that generated following rapamycin-mediated TOR kinase inhibition in *S. cerevisiae* revealed a very marked overlap (n = 125) in differentially regulated homologous genes (Table S4). This is in keeping with defined roles for TOR kinase function in *S. cerevisiae*, which includes the regulation of transcription in response to nutrients[26]. A strong correlation between developmental programming and microbial secondary metabolite biosynthesis has been well-documented[14] and while plasticity of nitrogen metabolism demonstrably supports *A. fumigatus* virulence[20] the role of the single, and likely essential, *A. fumigatus* TOR kinase homologue, TorA, (Afu2g10270) in this process remains untested. Intriguingly, however, a link between TOR kinase function and secondary metabolite production, partially through AreA, has recently been established in the rice pathogen *Fusarium fujikuroi* [44] where, in addition to the target genes in common with yeast and other eukaryotes, the AreA-regulated giberellin and bikaverin biosyntheis genes are also under the control of TOR. This raises the possibility of a relatively limited investment in secondary metabolism, within the context of our analysis, at the tested timepoint of murine infection.

Genes expressed during nitrogen starvation are also expressed during virulence in *Magneporthe grisea* [45], where two wide-range regulators of nitrogen catabolism genes, *NPR1* and *NPR2* [46] are required for virulence. Contrary to our analysis nitrate and nitrite reductase activities were found to be relevant for *M. grisea* during rice infection[45], however, a number of amino acid transporting proteins predominated among induced functions during both *in vitro* nitrogen starvation and infection. Common to that analysis, and ours, was increased abundance of proline oxidase (Afu3g02300) and proline permease (Afu2g11220, Afu7g10190, Afu8g02200) proteins, which, in the context of our experiment might have special relevance given the high hydroxyproline of collagen tissue, and the notable proline requirement of an attenuated *A. fumigatus* deletion mutant lacking the Ras-related protein RhbA[47]. To test the effect of nitrogen limitation on gene expression, in the context of the host adaptation study performed, we returned to the YPD reference sample employed for the initial analysis (sample F, Table S1), this time performing competitive hybridisations with doubly-amplified RNA obtained from a nitrogen-starved laboratory culture (sample G, Table S1). Given the predominance of hydroxyproline among collagen amino acids content, and its release into bronchoalveolar lavage fluid upon tissue injury[34] we adopted hydroxyproline as sole source of nitrogen for the starvation experiment. Hydroxyproline supported markedly slowed growth of *A. fumigatus* in liquid culture (Figure 7B) relative to YPD. We identified a significant overlap between the two datasets amounting to 280 genes. Functional anlaysis of the resulting datasets revealed that, in keeping with the murine adaptation dataset, ribosome biogenesis and protein biosynthesis were markedly down regulated, moreover, functions associated with mitosis and cell cycle were more abundantly represented among the categories favoured under nitrogen starvation, relative to rich laboratory growth. Given the differences between the tested media in terms of ability to support *A. fumigatus* growth (Figure 7B) it is pleasing to see that such a theme was not apparent from the murine adaptation dataset where the degree of nutrient limitation is unlikely to be as severe as that imposed in the nitrogen starvation conditions we used here. Interestingly 29% of the genes preferentially expressed during nitrogen starvation were subtelomerically located, and a degree of clustered gene expression was observable. Thus induction of the subtelomeric gene repertoire becomes important during nutrient deprivation, a trend which was not observable in response to any of the other *in vitro* stresses, other than neutrophil exposure the physiological relevance of which requires further investigation.

The uptake and catabolism of other amino acids released by proteolytic digestion of murine lung parenchyma might provide a source of nitrogen during *A. fumigatus* infection, which is supported in our analyses by increased transcript abundance of various secreted proteases (Table 2). Extracellular proteases are implicated as virulence actors in invasive aspergillosis[1]. An elastolytic protease, produced when *A. fumigatus* is cultured on the insoluble matrix from bovine lung is also produced during spore germination in infected lungs of neutropenic mice, as judged by immunogold cytochemical localization[48], and mutationally-derived mutants unable to produce this protease were deficient for virulence in the murine model. An elastinolytic metalloprotease, characterized by the same group was similarly visualized during murine lung infection[49]. Many species of human pathogenic fungi secrete proteases *in vitro* or during infection. Full virulence associated with *A. fumigatus* protease mutants [1],[50] is presumed to be due to redundancy among the many enzymes produced by this organism which may degrade the lung parenchyma to release utilisable carbon and nitrogen during infection. A directed analysis of the role of such proteins in virulence might now be possible on the strength of our data. Importantly, however, any conclusions reached on the basis of this study reflect the comparative nature of the anlaysis, thus transcripts equally abundant under both conditions tested will not have been be identified. Time course analyses of *A. fumigatus* growth during murine infection will reveal stage-specific gene expression in the absence of confounding sample treatments. These analyses are underway in our laboratories.

Fungal oxygen-sensing mechanisms have been linked to cell membrane sterol levels in *Schizosaccharomyces pombe* and *Cryptococcus neoformans* where homologues of the mammalian Sterol Regulatory Element Binding Protein (SREBP) transcription factors, in complex with SREBP cleavage-activating protein (SCAP) partners, undergo cellular translocation (from the endoplasmic reticulum to the golgi) prior to proteolytic SREBP activation[51],[52]. The *C. neoformans* SREBP homologue, Sre1p, plays an important role in low oxygen adaptation and infection[52]. Biosynthesis of sterols and unsaturated fatty acids is an aerobic process in *Saccharomyces cerevisiae* and in *C. neoformans*, the hypoxia-mimicking agent cobalt chloride and oxygen limitation target sterol biosynthetic gene expression. This was intriguing to us given the broadly observed reduction in transcript abundance among ergosterol biosynthetic genes, relative to laboratory culture (Table 2). Our initial hypothesis attributed this effect to oxygen limitation in the murine lung environment since airway obstruction, intraalveolar exudates and inflammation, or damage to alveolar capillaries (all observed in our murine modelling of pulmonary aspergillosis) pose a significant barrier to proper oxygenation in human lungs[53]. Oxygen deprivation in mammals leads to a transcriptional induction of genes for adaptation to hypoxia[54] and efforts to characterise the transcription profile of murine immune responses to *A. fumigatus* infection indicate that hypoxia is relevant in the neutropenic murine lung at 24 hours post-infection (Turnbull, personal communication). Co-hybridisation of murine lung cDNAs, derived from infected immunocompetent and immunocompromised mice with a murine immunology array set identified upregulation of murine ARNT (log_2_ ratio = 2.09) the obligate heterodimeric binding partner for the hypoxia induced factor HIF-1α[54]. However, on comparing the *A. fumigatus* murine adaptation gene expression signature to that observed following exposure to anaerobic stress *in vitro* (Figure 5A) no support for this hypothesis could be gleaned. Rather, repression of the two *A. fumigatus* 14-alpha sterol demethylase genes (Afu4g06890 and Afu Afu03740), representing a critical step in ergosterol biosynthesis, was observed following hydrogen peroxide-mediated oxygen stress. Therefore, it would seem that this component of the H_2_O_2_-mediated oxidative stress response is relevant *in vivo*. This was found to be distinct from antioxidative action of the *A. fumigatus* Mn superoxide dismutase (Afu1g14550) and the bifunctional catalase-peroxidase Cat2 (Afu8g01670), both of which were more abundant following murine lung (Table 2) or neutrophil exposure (Figures 6 and S2), suggesting multiple modes of oxidative stress encountered during murine infection. Interestingly H_2_O_2_ gradients are detectable across the *Saccharomyces cerevisiae* plasma membrane upon H_2_O_2_ exposure, suggesting a mechanism other than diffusion for H_2_O_2_ entry into cells. This, coupled with the observation that *S. cerevisiae* mutants *erg3Δ and erg6Δ*, having increased ergosterol biosynthesis, show increased permeability to H_2_O_2_ might suggest down-regulation of ergosterol biosynthesis as a protective response against oxidative stress[55].

All of the previously characterised components of the *A. fumigatus* siderophore biosynthetic pathway[17],[18] were more abundantly represented at transcript level during murine infection. Comparison of the murine dataset with that generated during *in vitro* iron limitation confirmed the importance of this environmental deficit during murine adaptation (Figure 6). With respect to extracellular iron mobilization fungal siderophores bind ferric iron with a high affinity, delivering the ferric chelate to specific receptors at the cell surface for translocation into the cytoplasm. Existing evidence supports an essential role for this uptake mechanism during infection[17],[18]. Reductive iron assimilation (RIA) is dispensable for murine virulence[17] but may assist in iron acquisition during infection since RIA inhibition in the absence of extracellular siderophore biosynthesis prevents growth *in vitro* [18]. This hypothesis is supported by our finding that numerous RIA components are induced during initiation of infection (Table 2) in addition to siderophore biosynthetic genes. We were also able to correlate differential gene expression following *in vitro* alkaline shift to the host adaptation transcriptome (Figure 5). This was to be expected given the broad requirement for fungal pH adaptation during mammalian pathogenesis[56] but is nonetheless pleasing to observe particularly given that the *in vitro* acid stress transcriptome showed an opposite trend (Figure S2) and since pleiotropic activity of the virulence-directing family of PacC/Rim101 pH sensing transcription factors[56] does not permit virulence defects to be soley, or thus far absolutely, correlated with pH growth phenotypes.

Fungal secondary metabolite biosynthesis is mediated at the level of transcription, from clusters of physically linked, co-ordinately regulated genes and is profoundly affected by environmental factors such as pH and nutrient availability[14]. For the most part, defined cues prompting gene expression from such clusters remain unknown, as do the functions of the molecules produced by them in the natural environment, but popular theory regards microbial secondary metabolites as chemical determinants of selective advantage[57]. Noting that clusters of physically linked, co-ordinately regulated genes were a prominent feature of our dataset we applied a custom script to systematically identify them. To limit the identification of false negatives from our analysis (due, for example, to poor spot quality or absence on array) we allowed for ‘gaps’ of up to a maximum of 4 non-up- or down- regulated genes permissable per cluster. Gap size variation influenced the architecture of clusters detectable in the dataset, further widening the extremities of pre-existing clusters rather than creating new ones (a complete breakdown of the cluster anlaysis is available as Dataset S2). Thus a gap size of zero identified 14 upregulated gene clusters and a gap size of one identified 38. The maximum gap size applied (gap size = 4) identified 65 groups of physically linked genes having increased transcript abundance during murine infection (Figure 3). A large proportion (34%) of up-regulated, physically clustered, genes was found to reside within 300 kb of chromosome ends (Figure 3 and Table 4). The clusters are comprised of up to 34 co-ordinately expressed genes and include loci directing biosynthesis of siderophores (cluster 33) and two known secondary metabolites, pseurotin and gliotoxin (clusters 70 and 59, respectively). The importance of fungal gene clusters in virulence has recently been characterised in the fungal plant pathogen, *Ustilago maydis* where co-induction of physically linked, secreted protein-encoding genes is seen during infection[58]. We were unable to find evidence of clustered secreted protein genes from our analyses of *A. fumigatus* (data not shown) however, from an evolutionary perspective, clusters 8, 40, 53, 68 (down-regulated), 69 and 70 (Table S6) deviate significantly from the least virulent sequenced species considered here, *N. fischeri*, and therefore merit further analysis. *N. fischeri* is extremely rarely identified as a human pathogen[59]–[61], while prolonged exposure to *A. clavatus* spores can cause extrinsic allergic alveolitis known as malt worker's lung[62]. From a functional perspective some relevant deductions regarding the clusters identifiedby our analyses might be possible using present genome annotations. However, closer scrutinization of clusters is required to address the likelihood that *A. fumigatus* genes located in them encode functions required for virulence. We anticipate that the inference of shared functionality among neighbouring co-regulated genes in our dataset will empower the functional annotation of the *A. fumigatus* genome; moreover, it significantly raises the profile of such gene-regulatory paradigms within the context of fungal pathogenicity.

Our observations of biased localization among genes overrepresented during growth in the murine lung (Figures 3 and 4, Table 3) prompted comparison of our dataset to that obtained by Perrin *et. al.* [16] who identified genes under control of the global seconday metabolism regulator, LaeA. Significant overlap is observable, notably among secondary metabolism genes, most specifically between genes in the gliotoxin and pseurotin biosynthetic gene clusters. Hypovirulence of a Δ*lae*A deletion mutant cannot be explained solely on the basis of a lack of gliotoxin biosynthesis, and no investigation of the role of other secondary metabolite clusters, with respect to virulence, has been undertaken in *A. fumigatus*, therefore at this time it is not possible to reach any conclusions on the contribution made to virulence by the molecules whose synthesis is directed by these loci. With regard to our comparison of functions under LaeA control and those important during murine infection, differing experimental conditions (Perrin study[16] performed at 25°C in liquid shaking culture and glucose minimal medium[63] for 60 hours) and methodologies pose a contentious issue. A truly illuminating analysis of LaeA activity during murine pathogenesis might be forthcoming from time course analyses in neutropenic mice, which we are currently attempting. DNA sequences in subtelomeric regions undergo ectopic recombination at a much higher rate than expected for homologous recombination[64] allowing the expansion and diversification of gene families located at chromosome ends. For some organisms gene expression is governed by subtelomeric localisation, for example two *A. nidulans* secondary metabolism gene clusters, directing penicillin and sterigmatocystin biosynthesis, are activated following deletion of the *hda*A histone deacetylase gene[65]. In other organisms subtelomeres provide an ideal setting for genes involved in antigenic variation, such as in the parasites *Plasmodium falciparum* and *Trypanosoma brucei* [66] and in cytoadhesion, such as the EPA family of *Candida glabrata* adhesins[67]. Thus the importance of sub-telomeric chromosomal regions within the context of eukaryotic pathogenesis is gaining significance in this post-genomic era of microbial studies.

These analyses of transcript abundance, drawn from minute quantities of fungal material convey a programme of *A. fumigatus* cellular regulation directly from the site of pulmonary infection. From a fungal physiological perspective the mammalian host restricts iron, and likely various nutrients, as well as exerting multiple degrees of oxidative stress. Regarded as a function of the genomic landscape the observed transcriptional changes reveal genome organisation and subtelomeric diversity as effectors of the remarkable versatility of *A. fumigatus* with respect to the niches it successfully inhabits, one of which is the neutropenic human lung.

## Materials and Methods

Full details of the methods used are available through Array Express (http://www.ebi.ac.uk/microarray-as/aer/#ae-main0) Accession number E-TABM-327. The sequenced *A. fumigatus* isolate Af293 has been previously described[68] and was used for all analysis reported in this study.

### *A. fumigatus* strains and growth conditions

*A. fumigatus* Af293 *in vitro* isolates for murine infection experiments were grown in shaken liquid culture at 37°C in YPD medium. For oxidative stress Af293 conidia were inoculated into *Aspergillus* complete medium (CM)[69] and grown for 16 hours with shaking at 37°C. The resultant hyphal culture was transferred to CM containing 17 mM H_2_O_2_ and aliquots were harvested for RNA isolation upon transfer (T_0_) and at 60 minutes after the initiation of H_2_O_2_ exposure. For acid stress Af293 conidia were incubated in liquid CM medium with shaking for 6 hours at 37°C. At that time the culture was split, one portion of the culture being adjusted to pH3 using HCl (time T_0_). Aliquots of each were taken at time 60 mins for RNA purification and microarray expression analysis. pH 3 was verified at T_0_ and at the end of the time course. For alkaline stress *A. fumigatus* germlings were cultured for 16 hours in shaken liquid AMM containing 100 mM glycolic acid pH5.0, 10 ml/L vitamin solution[7], 5 mM ammonium tartrate and 1% w/v glucose. Germlings were filtered using Miracloth and shifted to similar, prewarmed medium containing 100 mM Tris-HCl pH8.0. After 1 hour incubation at 37°C, mycelia were washed with cold AMM pH5.0 and RNA extraction was performed immediately. For anaerobic stress hyphae were grown in CM (10^7^ conidia in 20 ml in 100 mm Petri dishes without agitation) for 16 hours at 37°C. Three plates were placed in anaerobic chambers and anaerobic conditions were established using 2 palladium catalysts (GasPak Plus, Becton Dickinson). A control plate was placed in a chamber without palladium catalysts and all plates were returned to a 37°C incubator for the duration of the experiment. Plates placed in anaerobic chambers were removed at 60 minutes, the mycelium rapidly harvested on Miracloth, rinsed with ice-cold water and rapidly frozen in liquid nitrogen. The RNA was extracted and used in competitive hybridization with the 16 hour control RNA sample. For neutrophil exposure 10^7^ germlings grown in 20 ml of RPMI1640 with L-glutamine, 25 mM Hepes, and 5% (v/v) fetal bovine serum for 7 hours at 37°C in 100 mm Petri dishes were exposed to 10^7^ human neutrophils. At 60 minutes of exposure to neutrophils, RNA was prepared. For iron limitation *A. fumigatus* isolate ATCC46645 was grown for 15 hours at 37°C in –Fe *Aspergillus* minimal medium (AMM, iron depleted conditions) according to Pontecorvo[63] containing 1% (wt/vol) glucose as carbon source, 20 mM glutamine as nitrogen source. After ths time, 10 µM FeSO_4_ was added to the medium and germlings harvested after a further 60 minutes. Growth curve analyses were performed in triplicate using liquid YPD, or AMM containing 1% glucose and either 5 mM ammonium tartrate or 5 mM hyroxyproline as nitrogen source. Cultures were inoculated to a final concentration of 5×10^6^ spores/ml and incubated under aerobic conditions at 37°C with shaking at 150 rpm. At selected timepoints mycelia were harvested on Miracloth, encased in Whatmann paper and dried at 37°C for 48 hours before weighing.

### Murine infections

Groups of 24 outbred male mice (strain CD1, 18–22 g, Harlan Ortech) were housed in individually vented cages and allowed free access to food and water. Mice received cyclophosphamide (150 mg/kg, ENDOXANA, Asta Medica) by intraperitoneal injection on days −3 and −1. A single dose of hydrocortisone acetate (112.5 mg/kg, HYDROCORTISTAB, Sovereign Medical) was administered subcutaneously on day −1. All mice received tetracycline hydrochloride 1 mg/l and ciprofloxacin 64 mg/l in drinking water as prophylaxis against bacterial infection. *Aspergillus* spores for inoculations were grown on solid ACM, containing 5 mM ammonium (+)-tartrate and 1% (w/v) Oxoid Agar Number 3 for 5 days prior to infection. Conidia were freshly harvested using sterile saline (Baxter Healthcare Ltd. England) and filtered through MIRACLOTH (Calbiochem). Conidial suspensions were spun for 5 minutes at 3000 *g*, washed twice with sterile saline, counted using a hemocytometer and re-suspended at a concentration of 2.5×10^10^ colony forming units (c.f.u)/ml. Viable counts from administered inocula were determined following serial dilution by plating on *Aspergillus* complete medium and growth at 37 °C. Mice were anesthetized by halothane inhalation and infected by intranasal instillation of 10^8^ conidia in 40 µl of saline. Groups of infected mice were culled and processed collectively during a 2 hour window corresponding to a time point 12–14 hours post-infection. Bronchoalveolar lavage was performed immediately after culling using three 0.5 ml aliquots of pre-warmed sterile saline. BALFs were snap frozen immediately following harvest using liquid nitrogen.

### RNA extraction and amplification

Prior to RNA extraction snap-frozen BALF samples were centrifuged, washed with 1 ml of ice-cold sterile water to lyse contaminating host cells and pooled. Following a further cycle of snap freezing pelleted pooled product was homogenized using a pestle and mortar and liquid nitrogen. RNA extraction was performed immediately using the ‘filamentous fungi’ protocol of the RNeasy mini kit (Qiagen). RNA concentration and integrity was measured by Nanodrop. Reference RNA was prepared from snap frozen ground mycelium. RNA from reference samples was prepared from snap-frozen homogenized germlings or young mycelium.

### Estimation of fidelity of transcript abundance following mRNA amplification

The corrected, multiple t-testing methodology of Nygaard[23] formed the basis of our investigation. However, for computational ease it was modified to use the limma linear modelling framework for the R statistical environment.

Spots were filtered as previously described and systematic spatial effects were normalized within arrays using the print-tip lowess method (limma package for R); no between-array normalization was performed in order to preserve the amplification-protocol variation existent between arrays. Least squares regression was used to fit the average log ratio, and error term of each transcript in each of the amplification protocols. The resulting coefficient vectors contained the mean relative abundance of each transcript (T0 v T60) in one of the three amplification protocols: aRNA_r1_, aRNA_r2_ or totRNA. The amplification protocol effects and their associated Benjamini-Hochberg corrected *p*-values (contrast_1_, contrast_2_ and contrast_3_ in Figure S1) were extracted by fitting a contrast matrix to the original linear model. We defined the null hypothesis H_0_ as no difference in log ratios due to the amplification protocol, and an alternative hypothesis H_1_ as loss of log ratio fidelity due to the amplification protocol. Spots were partitioned into the three sets based on the Benjamini-Hochberg adjusted p-values associated with each of the fitted contrasts. The rejected, undetermined and conservative sets were populated by genes whose adjusted p-values were 0<*p*<0.01, 0.01<*p*<0.1 and ≫0.1<*p*<1 respectively. This approach rejected 2325 (8.49%) of the spots in the aRNA_1_ - totRNA comparison, i.e. the genes whose log ratios are significantly altered as a result of 1 round of mRNA amplification. The analogous rejected sets for the aRNA_2_ – totRNA and aRNA_1_ – aRNA_2_ comparisons contained 2604 (9.52%) and 73 (0.27%) rejected spots respectively. Comparison of the rejection sets showed that 80.69% of the spots in the aRNA_1_- totRNA rejection set were also present in the aRNA_2_- totRNA rejection set. This finding, in addition to the observed small number of genes in the aRNA_1_ – aRNA_2_ rejection set, suggests that most of the amplification specific noise is introduced in the first round.

### Microarray hybridisations

All experiments used Af293 DNA amplicon microarrays[68]. Labelling reactions with RNA and hybridisations were conducted as described in the TIGR standard operating procedures found at http://atarrays.tigr.org. Independent verification of selected log_2_ ratios was achieved using quantitative RT-PCR (See Figure S3 and Table S9).

Hybridised slides were scanned using the Axon GenePix 4000B microarray scanner and the TIFF images generated were analyzed using TIGR Spotfinder (http://www.tigr.org/software/) to identify poor quality spots. mRNA underwent two rounds of linear amplification by T7 promoter-directed transcription. Data processing and analysis was performed in the R statistical framework, using the limma, multcomp and fdrtool packages (http://www.bioconductor.org). The intensity data retained after filtering (91.5% of the total spots) were normalized within (print-tip lowess) and between (scale) arrays. Least squares regression (lmFit) was then used to estimate a vector of regression coefficients (representing the mean log2 (Cy5/Cy3) across the 5 biological replicates for the *in vivo* effect), the associated residuals and unadjusted *p*-values. Differentially regulated transcripts (Benjamini-Hochberg corrected *p*-value<0.05) were further categorized by the magnitude and direction of the fitted log ratios. Up-regulated transcripts were defined as having log2 (Cy5/Cy3) greater than an arbitrary threshold of plus, or minus, two. Accuracy of microarray data was independently verified by quantitative RT-PCR on selected transcripts (See Figure S3). Oligonucleotides used for this analysis are detailed in Table S9).

For exposure to *in vitro* stresses microarray experiments were performed as previously described[68].

### Automated biological theme determinations

To identify over-represented Gene Ontology terms, loci showing significantly different expression were further analyzed by the Expression Analysis Systematic Explorer (EASE) (PMID:14519205), which is implemented in MEV within the TIGR TM4 microarray data analysis suite (http://TM4.org). Numbers of genes in the indicated Gene Ontology categories were subjected to statistical analysis by EASE[25] to identify categories overrepresented compared with the whole genome data set. Only categories with Fisher's exact test *p*-values<0.05 were included.

### Core and lineage-specific gene sets

Orthologous proteins in the genomes were identified using a reciprocal-best-BLAST-hit (RBH) approach with a cut-off of 1e-05. The orthologous clusters, as well as synteny visualization and comparative analysis tools can be also found in the *Aspergillus* Comparative Database at http://www.tigr.org/sybil/asp.

### Gene cluster identifications

Clusters of co-regulated neighbouring genes were defined using a simple, two parameter algorithm. The first parameter, minimum_block_size (mbs), specifies the minimum number of up- or down- regulated genes that a cluster must contain. The second parameter, maximum_gap_size (mgs), specifies the maximum number of adjacent non-up- or down- regulated genes permissable per cluster. Four analyses were performed corresponding to mgs values of n = 0, 1, 2, 3 and 4. Figure 3 shows data from mgs = 4. Assigned cluster numbers are based on chromosomal position. More details on the clustering algorithm are available at http://sybil.sourceforge.net/documentation.html#algorithms. Secondary metabolite biosynthesis genes were identified using SMURF, a web-based software tool (http://www.tigr.org/software/genefinding.shtml - temporarily hosted at http://binf.gmu.edu/fseifudd/smurf/index1.html). Density of the SM genes was estimated as the exon length per kb.

## Supporting Information

Figure S1 Schematic representation of hybridisations for assessing maintenance of relative transcript abundance during *A. fumigatus* mRNA amplification. Boxes represent the biological source material from which the RNA was isolated. The open and shaded arrows depict dye-swapped hybridisations for all pairs of samples. For all comparisons Af293-derived cDNA sampled at media shift (t0) was cohybridised with Af-derived cDNA sampled at 60 minutes post-shift (t60). Horizontally aligned boxes depict cohybridisation of samples subjected to one (aRNA1), two (aRNA2) or zero (totRNA) rounds of linear mRNA amplification. Amplification protocols were compared indirectly by fitting a contrast matrix (contrast1, contrast2, contrast3).
(0.03 MB JPG)

Figure S2 Comparative analysis of *A. fumigatus* gene expression datsets. A pan-experimental comparison of *A. fumigatus* gene expression aligning log2 ratios obtained during host adaptation (mice); exposure to neutrophils (neut), increased expression in parental strain versus Δ*lae*A mutant, acid shift (acid), iron starvation (iron), oxygen depletion (anaer) and oxidative stress (H2O2) for various genes. The colour bar indicates the range of log2 expression ratios, grey bars indicate genes from which signals were undetectable for technical reasons. Experimental conditions are described in Materials and Methods. LaeA dataset is taken from Perrin et. al. [16]. Comparative analyses were implemented in TM4 http://www.jcvi.org/cms/research/software/
(70.39 MB TIF)

Figure S3 Concordance between estimates of relative abundance from triplicate qPCR measurements (yellow) and microarray experiments (grey) for selected *A. fumigatus* ORFs. Oligo sequences are given in Table S9.
(1.00 MB TIF)

Table S1 RNA samples
(0.05 MB DOC)

Table S2 Filtered genes
(0.03 MB DOC)

Table S3 GO analysis
(0.85 MB DOC)

Table S4 TOR analysis
(0.37 MB DOC)

Table S5 Asterisked gene lists
(0.34 MB DOC)

Table S6 Cluster overview
(1.33 MB DOC)

Table S7 Nitrogen starvation GO
(0.40 MB XLS)

Table S8 Nitrogen starvation gene clusters
(0.03 MB XLS)

Table S9 Oligo sequences for RT-PCR
(0.04 MB DOC)

Dataset S1 (0.58 MB XLS)

Dataset S2 Gene clusters fully annotated
(0.19 MB XLS)

Dataset S3 Lists of genes common to several analyses
(0.04 MB XLS)

Dataset S4 (0.93 MB XLS)
