# Supplementary figures and images for: Sub-Telomere Directed Gene Expression during Initiation of Invasive Aspergillosis
Source: PLoS Pathog. 2008 Sep 12;4(9):e1000154. doi: 10.1371/journal.ppat.1000154 (PMC2526178; doi:10.1371/journal.ppat.1000154)

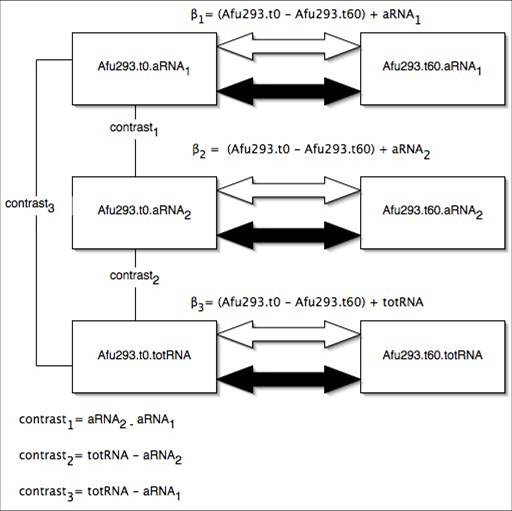

Supplement: Figure S1 — Schematic representation of hybridisations for assessing maintenance of relative transcript abundance during A. fumigatus mRNA amplification. Boxes represent the biological source material from which the RNA was isolated. The open and shaded arrows depict dye-swapped hybridisations for all pairs of samples. For all comparisons Af293-derived cDNA sampled at media shift (t0) was cohybridised with Af-derived cDNA sampled at 60 minutes post-shift (t60). Horizontally aligned boxes depict cohybridisation of samples subjected to one (aRNA1), two (aRNA2) or zero (totRNA) rounds of linear mRNA amplification. Amplification protocols were compared indirectly by fitting a contrast matrix (contrast1, contrast2, contrast3). (0.03 MB JPG) [file ppat.1000154.s001.jpg]

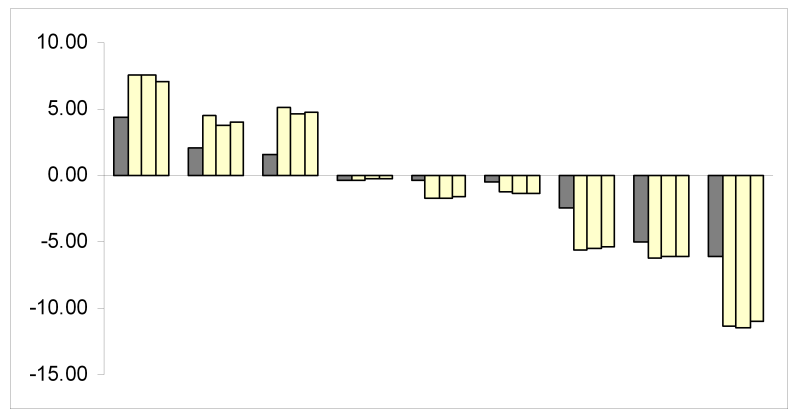

Supplement: Figure S3 — Concordance between estimates of relative abundance from triplicate qPCR measurements (yellow) and microarray experiments (grey) for selected A. fumigatus ORFs. Oligo sequences are given in Table S9. (1.00 MB TIF) [file ppat.1000154.s003.tif]
