# Supplementary material for: Sub-Telomere Directed Gene Expression during Initiation of Invasive Aspergillosis
Source: PLoS Pathog. 2008 Sep 12;4(9):e1000154. doi: 10.1371/journal.ppat.1000154 (PMC2526178; doi:10.1371/journal.ppat.1000154)
Supplement: Table S1 — RNA samples (0.05 MB DOC) [file ppat.1000154.s004.doc]

| Sample ID | *A. fumigatus* strain | Total RNA (ng) | ~ mRNA (ng)  (2% total) | aRNA (µg)  1^st^ round | aRNA (µg)  2^nd^ round  (Using 1 µg from round 1) | Amplification factor |
| --- | --- | --- | --- | --- | --- | --- |
| T0 | Overnight broth culture | 1000 | 20 | 25.95 | 184.76 | 9.2 x 10^3^ |
| T60 | Overnight broth plus media shift for 60 minutes | 1000 | 20 | 87.33 | 174.6 | 8.7 x 10^3^ |
| A | (*in vivo*)_1_ | 322.8 | 12.9 | 11.08 | 251.14 | 2 x 10^4^ |
| B | (*in vivo*)_2_ | 130 | 2.6 | 6.25 | 164.02 | 6.3 x 10^4^ |
| C | (*in vivo*)_3_ | 240 | 9.6 | 9.02 | 172.63 | 1.8 x 10^4^ |
| D | (*in vivo*)_4_ | 800 | 64 | 19.27 | 177.11 | 2.8 x 10^3^ |
| E | (*in vivo*)_5_ | 110 | 2.2 | 12.27 | 258.39 | 1.2 x 10^5^ |
| F | (*in vitro*) | 1136 | 22.72 | 3.72 | 186.96 | 8.2 x 10^3^ |
| G | (*in vitro*)  hydroxyproline | 1000 | 20 | 56 | 566 | 2.8 x 10^4^ |

**Table S1**

**Estimates of RNA quantities and resulting aRNA yields for samples employed**

**this study.** Calculation of the amplification factor is based upon the assumption that mRNA represents 2% of total RNA. T0 and T60 were prepared from *A. fumigatus* Af293 mycelium grown in shaken liquid YPD culture at 37°C for 16 hours. T0 was harvested and snap frozen. T60 was shifted into fresh YPD medium and cultured for a further 60 minutes prior to mycelial harvest. Samples A to E represent the five biological replicates employed for this study (*in vivo*)_1-5_. Sample F represents the reference RNA sample for the infection and nitrogen starvation experiments (Figure 1, Panel C) and was extracted from germlings cultured in YPD for 12 hours at 37°C in shaken liquid YPD culture. Sample G represents RNA extracted from nitrogen-starved germlings.
