# Supplementary material for: Sub-Telomere Directed Gene Expression during Initiation of Invasive Aspergillosis
Source: PLoS Pathog. 2008 Sep 12;4(9):e1000154. doi: 10.1371/journal.ppat.1000154 (PMC2526178; doi:10.1371/journal.ppat.1000154)
Supplement: Table S2 — Filtered genes (0.03 MB DOC) [file ppat.1000154.s005.doc]

**Table S2**

Filtering per array in the amplification control study.

Filtering criterion A refers to spots flagged by the TIGR spotfinder software. Filtering criterion B excludes spots whose foreground intensity is less than twice its background intensity in either the Cy5 or Cy3 channel.

| Array Number | 1 | 2 | 3 | 4 | 5 | 6 |
| --- | --- | --- | --- | --- | --- | --- |
| Criterion A | 1895 | 2080 | 1867 | 3630 | 8079 | 7151 |
|  | 6.33% | 6.94% | 6.23% | 12.11% | 26.97% | 23.90% |
| Criterion B | 2147 | 2008 | 1687 | 1827 | 6584 | 6197 |
|  | 7.17% | 6.70% | 5.63% | 6.10% | 21.98% | 20.69% |
| Sum | 4042 | 4088 | 3554 | 5457 | 14663 | 13348 |
|  | 13.49% | 13.65% | 11.87% | 18.22% | 48.95% | 44.56% |
