# Supplementary material for: Sub-Telomere Directed Gene Expression during Initiation of Invasive Aspergillosis
Source: PLoS Pathog. 2008 Sep 12;4(9):e1000154. doi: 10.1371/journal.ppat.1000154 (PMC2526178; doi:10.1371/journal.ppat.1000154)
Supplement: Table S3 — GO analysis (0.85 MB DOC) [file ppat.1000154.s006.doc]

**Table S3**

**Over-represented Gene Ontology (GO) terms amongst genes having increased transcript abundance.**

| **GO ID** | **GO Term** | **List hits** | **List size** | **Pop.hits** | **Pop. Size** | ***p value*** |
| --- | --- | --- | --- | --- | --- | --- |
| **Biological process** | |  |  |  |  |  |
| GO:0019538 | protein metabolism | 174 | 561 | 781 | 4219 | 7.35E-15 |
| GO:0044267 | cellular protein metabolism | 159 | 561 | 695 | 4219 | 1.52E-14 |
| GO:0044260 | cellular macromolecule metabolism | 168 | 561 | 775 | 4219 | 4.09E-13 |
| GO:0043170 | macromolecule metabolism | 254 | 561 | 1383 | 4219 | 2.27E-11 |
| GO:0042254 | ribosome biogenesis and assembly | 51 | 561 | 161 | 4219 | 5.56E-10 |
| GO:0007028 | cytoplasm organization and biogenesis | 51 | 561 | 161 | 4219 | 5.56E-10 |
| GO:0044237 | cellular metabolism | 366 | 561 | 2251 | 4219 | 6.51E-10 |
| GO:0007046 | ribosome biogenesis | 46 | 561 | 140 | 4219 | 1.08E-09 |
| GO:0016072 | rRNA metabolism | 41 | 561 | 118 | 4219 | 1.37E-09 |
| GO:0006364 | rRNA processing | 40 | 561 | 115 | 4219 | 2.12E-09 |
| GO:0044238 | primary metabolism | 334 | 561 | 2028 | 4219 | 3.30E-09 |
| GO:0006457 | protein folding | 23 | 561 | 50 | 4219 | 1.48E-08 |
| GO:0006412 | protein biosynthesis | 72 | 561 | 302 | 4219 | 1.63E-07 |
| GO:0008152 | metabolism | 394 | 561 | 2570 | 4219 | 5.28E-07 |
| GO:0043037 | translation | 30 | 561 | 96 | 4219 | 3.12E-06 |
| GO:0016070 | RNA metabolism | 58 | 561 | 247 | 4219 | 4.91E-06 |
| GO:0009059 | macromolecule biosynthesis | 74 | 561 | 346 | 4219 | 9.30E-06 |
| GO:0006365 | 35S primary transcript processing | 18 | 561 | 46 | 4219 | 9.78E-06 |
| GO:0006413 | translational initiation | 17 | 561 | 42 | 4219 | 1.01E-05 |
| 166 | RNA processing | 43 | 561 | 175 | 4219 | 2.76E-05 |
| GO:0030490 | processing of 20S pre-rRNA | 15 | 561 | 37 | 4219 | 3.32E-05 |
| GO:0006996 | organelle organization and biogenesis | 100 | 561 | 522 | 4219 | 3.55E-05 |
| GO:0009058 | biosynthesis | 123 | 561 | 696 | 4219 | 1.91E-04 |
|  | mitochondrial electron transport, ubiquinol to cytochrome c | 5 | 561 | 6 | 4219 | 2.19E-04 |
| GO:0001302 | replicative cell aging | 6 | 561 | 9 | 4219 | 3.17E-04 |
| GO:0000027 | ribosomal large subunit assembly and maintenance | 11 | 561 | 28 | 4219 | 5.25E-04 |
| GO:0007569 | cell aging | 6 | 561 | 11 | 4219 | 1.38E-03 |
| GO:0006359 | regulation of transcription from RNA polymerase III promoter | 4 | 561 | 5 | 4219 | 1.39E-03 |
| GO:0006360 | transcription from RNA polymerase I promoter | 7 | 561 | 15 | 4219 | 1.73E-03 |
| GO:0009987 | cellular process | 461 | 561 | 3265 | 4219 | 1.78E-03 |
| GO:0043283 | biopolymer metabolism | 151 | 561 | 930 | 4219 | 1.96E-03 |
| GO:0042257 | ribosomal subunit assembly | 12 | 561 | 37 | 4219 | 2.13E-03 |
| GO:0000128 | flocculation | 3 | 561 | 3 | 4219 | 2.34E-03 |
| GO:0006356 | regulation of transcription from RNA polymerase I promoter | 3 | 561 | 3 | 4219 | 2.34E-03 |
| GO:0044249 | cellular biosynthesis | 105 | 561 | 616 | 4219 | 2.34E-03 |
| GO:0042255 | ribosome assembly | 13 | 561 | 43 | 4219 | 2.85E-03 |
| GO:0043412 | biopolymer modification | 73 | 561 | 406 | 4219 | 2.95E-03 |
| GO:0050875 | cellular physiological process | 454 | 561 | 3222 | 4219 | 3.23E-03 |
| GO:0007568 | aging | 6 | 561 | 13 | 4219 | 4.03E-03 |
| GO:0006783 | heme biosynthesis | 6 | 561 | 13 | 4219 | 4.03E-03 |
| GO:0042168 | heme metabolism | 6 | 561 | 13 | 4219 | 4.03E-03 |
| GO:0000154 | rRNA modification | 6 | 561 | 13 | 4219 | 4.03E-03 |
| GO:0006628 | mitochondrial translocation | 7 | 561 | 17 | 4219 | 4.12E-03 |
| GO:0006508 | proteolysis | 27 | 561 | 125 | 4219 | 6.25E-03 |
| GO:0006778 | porphyrin metabolism | 6 | 561 | 14 | 4219 | 6.28E-03 |
| GO:0006779 | porphyrin biosynthesis | 6 | 561 | 14 | 4219 | 6.28E-03 |
| GO:0006626 | protein targeting to mitochondrion | 6 | 561 | 14 | 4219 | 6.28E-03 |
| GO:0006461 | protein complex assembly | 19 | 561 | 79 | 4219 | 6.30E-03 |
| GO:0016337 | cell-cell adhesion | 4 | 561 | 7 | 4219 | 7.77E-03 |
| GO:0009060 | aerobic respiration | 13 | 561 | 48 | 4219 | 8.02E-03 |
| GO:0045333 | cellular respiration | 13 | 561 | 48 | 4219 | 8.02E-03 |
| GO:0016043 | cell organization and biogenesis | 135 | 561 | 849 | 4219 | 8.05E-03 |
| GO:0006696 | ergosterol biosynthesis | 11 | 561 | 38 | 4219 | 8.45E-03 |
| GO:0008204 | ergosterol metabolism | 11 | 561 | 38 | 4219 | 8.45E-03 |
| GO:0007155 | cell adhesion | 5 | 561 | 11 | 4219 | 9.46E-03 |
| GO:0042273 | ribosomal large subunit biogenesis | 5 | 561 | 11 | 4219 | 9.46E-03 |
| GO:0018193 | peptidyl-amino acid modification | 5 | 561 | 11 | 4219 | 9.46E-03 |
| GO:0006091 | generation of precursor metabolites and energy | 32 | 561 | 161 | 4219 | 1.11E-02 |
| GO:0006464 | protein modification | 64 | 561 | 373 | 4219 | 1.52E-02 |
|  | mitochondrial electron transport, succinate to ubiquinone | 2 | 561 | 2 | 4219 | 1.77E-02 |
| GO:0042126 | nitrate metabolism | 2 | 561 | 2 | 4219 | 1.77E-02 |
| GO:0042128 | nitrate assimilation | 2 | 561 | 2 | 4219 | 1.77E-02 |
| GO:0044257 | cellular protein catabolism | 20 | 561 | 93 | 4219 | 1.81E-02 |
| GO:0046148 | pigment biosynthesis | 7 | 561 | 22 | 4219 | 1.99E-02 |
| GO:0042440 | pigment metabolism | 7 | 561 | 22 | 4219 | 1.99E-02 |
| GO:0009451 | RNA modification | 9 | 561 | 32 | 4219 | 2.01E-02 |
| GO:0051603 | proteolysis during cellular protein catabolism | 19 | 561 | 88 | 4219 | 2.01E-02 |
| GO:0016126 | sterol biosynthesis | 11 | 561 | 43 | 4219 | 2.18E-02 |
| GO:0015980 | energy derivation by oxidation of organic compounds | 24 | 561 | 120 | 4219 | 2.41E-02 |
| GO:0006383 | transcription from RNA polymerase III promoter | 7 | 561 | 24 | 4219 | 3.20E-02 |
| GO:0019941 | modification-dependent protein catabolism | 18 | 561 | 87 | 4219 | 3.47E-02 |
| GO:0043632 | modification-dependent macromolecule catabolism | 18 | 561 | 87 | 4219 | 3.47E-02 |
| GO:0000002 | mitochondrial genome maintenance | 5 | 561 | 15 | 4219 | 3.91E-02 |
| GO:0000283 | establishment of cell polarity (sensu Saccharomyces) | 5 | 561 | 15 | 4219 | 3.91E-02 |
| GO:0016125 | sterol metabolism | 11 | 561 | 47 | 4219 | 4.04E-02 |
| GO:0006139 | nucleobase, nucleoside, nucleotide and nucleic acid metabolism | 127 | 561 | 839 | 4219 | 4.63E-02 |
| GO:0006694 | steroid biosynthesis | 11 | 561 | 48 | 4219 | 4.64E-02 |
| GO:0030163 | protein catabolism | 21 | 561 | 109 | 4219 | 4.80E-02 |
| GO:0042798 | protein neddylation during NEDD8 class-dependent protein catabolism | 2 | 561 | 3 | 4219 | 4.83E-02 |
| GO:0045041 | protein import into mitochondrial intermembrane space | 2 | 561 | 3 | 4219 | 4.83E-02 |
| GO:0018195 | peptidyl-arginine modification | 2 | 561 | 3 | 4219 | 4.83E-02 |
| GO:0019942 | NEDD8 class-dependent protein catabolism | 2 | 561 | 3 | 4219 | 4.83E-02 |
| GO:0006534 | cysteine metabolism | 2 | 561 | 3 | 4219 | 4.83E-02 |
| GO:0016574 | histone ubiquitination | 2 | 561 | 3 | 4219 | 4.83E-02 |
| GO:0006549 | isoleucine metabolism | 2 | 561 | 3 | 4219 | 4.83E-02 |
| GO:0006839 | mitochondrial transport | 6 | 561 | 21 | 4219 | 5.04E-02 |
| GO:0006512 | ubiquitin cycle | 22 | 561 | 116 | 4219 | 5.09E-02 |
| GO:0006511 | ubiquitin-dependent protein catabolism | 17 | 561 | 85 | 4219 | 5.23E-02 |
| GO:0043285 | biopolymer catabolism | 31 | 561 | 177 | 4219 | 6.14E-02 |
| 137 | tRNA aminoacylation | 6 | 561 | 22 | 4219 | 6.18E-02 |
| GO:0006418 | tRNA aminoacylation for protein translation | 6 | 561 | 22 | 4219 | 6.18E-02 |
| GO:0006403 | RNA localization | 8 | 561 | 33 | 4219 | 6.25E-02 |
| GO:0046112 | nucleobase biosynthesis | 4 | 561 | 12 | 4219 | 6.38E-02 |
| GO:0006399 | tRNA metabolism | 11 | 561 | 52 | 4219 | 7.62E-02 |
| GO:0008202 | steroid metabolism | 11 | 561 | 52 | 4219 | 7.62E-02 |
| GO:0044265 | cellular macromolecule catabolism | 31 | 561 | 181 | 4219 | 7.83E-02 |
| GO:0019856 | pyrimidine base biosynthesis | 3 | 561 | 8 | 4219 | 7.83E-02 |
| GO:0006743 | ubiquinone metabolism | 3 | 561 | 8 | 4219 | 7.83E-02 |
| GO:0016571 | histone methylation | 3 | 561 | 8 | 4219 | 7.83E-02 |
| GO:0043414 | biopolymer methylation | 5 | 561 | 18 | 4219 | 7.99E-02 |
| GO:0032259 | methylation | 5 | 561 | 18 | 4219 | 7.99E-02 |
| GO:0006090 | pyruvate metabolism | 7 | 561 | 29 | 4219 | 8.05E-02 |
| GO:0006730 | one-carbon compound metabolism | 7 | 561 | 29 | 4219 | 8.05E-02 |
| GO:0008151 | cell growth and/or maintenance | 4 | 561 | 13 | 4219 | 8.28E-02 |
| GO:0015931 | nucleobase, nucleoside, nucleotide and nucleic acid transport | 8 | 561 | 35 | 4219 | 8.38E-02 |
| GO:0007005 | mitochondrion organization and biogenesis | 9 | 561 | 41 | 4219 | 8.53E-02 |
| GO:0006080 | mannan metabolism | 2 | 561 | 4 | 4219 | 8.81E-02 |
| GO:0006097 | glyoxylate cycle | 2 | 561 | 4 | 4219 | 8.81E-02 |
| GO:0016074 | snoRNA metabolism | 2 | 561 | 4 | 4219 | 8.81E-02 |
| GO:0006835 | dicarboxylic acid transport | 2 | 561 | 4 | 4219 | 8.81E-02 |
| GO:0046487 | glyoxylate metabolism | 2 | 561 | 4 | 4219 | 8.81E-02 |
|  | deadenylation-dependent decapping | 2 | 561 | 4 | 4219 | 8.81E-02 |
| **Molecular Function** | |  |  |  |  |  |
| GO:0003754 | chaperone activity | 17 | 568 | 35 | 4324 | 3.76E-07 |
| GO:0003743 | translation initiation factor activity | 16 | 568 | 34 | 4324 | 1.43E-06 |
| GO:0008135 | translation factor activity, nucleic acid binding | 20 | 568 | 50 | 4324 | 1.71E-06 |
| GO:0045182 | translation regulator activity | 21 | 568 | 55 | 4324 | 2.32E-06 |
| GO:0016627 | oxidoreductase activity, acting on the CH-CH group of donors | 11 | 568 | 19 | 4324 | 5.11E-06 |
| GO:0030515 | snoRNA binding | 11 | 568 | 21 | 4324 | 1.86E-05 |
| GO:0003723 | RNA binding | 27 | 568 | 100 | 4324 | 1.39E-04 |
| GO:0003735 | structural constituent of ribosome | 28 | 568 | 112 | 4324 | 4.28E-04 |
| GO:0003724 | RNA helicase activity | 13 | 568 | 39 | 4324 | 9.36E-04 |
| GO:0016628 | oxidoreductase activity, acting on the CH-CH group of donors, NAD or NADP as acceptor | 4 | 568 | 5 | 4324 | 1.32E-03 |
| GO:0004680 | casein kinase activity | 4 | 568 | 5 | 4324 | 1.32E-03 |
| GO:0004004 | ATP-dependent RNA helicase activity | 10 | 568 | 27 | 4324 | 1.46E-03 |
| GO:0008186 | RNA-dependent ATPase activity | 10 | 568 | 27 | 4324 | 1.46E-03 |
| GO:0004682 | protein kinase CK2 activity | 3 | 568 | 3 | 4324 | 2.26E-03 |
| GO:0016274 | protein-arginine N-methyltransferase activity | 3 | 568 | 4 | 4324 | 8.14E-03 |
| GO:0016273 | arginine N-methyltransferase activity | 3 | 568 | 4 | 4324 | 8.14E-03 |
| GO:0004176 | ATP-dependent peptidase activity | 3 | 568 | 4 | 4324 | 8.14E-03 |
| GO:0008026 | ATP-dependent helicase activity | 11 | 568 | 40 | 4324 | 1.16E-02 |
| GO:0016876 | ligase activity, forming aminoacyl-tRNA and related compounds | 10 | 568 | 35 | 4324 | 1.20E-02 |
| GO:0016875 | ligase activity, forming carbon-oxygen bonds | 10 | 568 | 35 | 4324 | 1.20E-02 |
| GO:0004812 | aminoacyl-tRNA ligase activity | 10 | 568 | 35 | 4324 | 1.20E-02 |
| GO:0003773 | heat shock protein activity | 4 | 568 | 8 | 4324 | 1.33E-02 |
| GO:0008369 | obsolete molecular function | 38 | 568 | 204 | 4324 | 1.43E-02 |
| GO:0004175 | endopeptidase activity | 13 | 568 | 52 | 4324 | 1.46E-02 |
| GO:0004386 | helicase activity | 16 | 568 | 69 | 4324 | 1.49E-02 |
| GO:0008565 | protein transporter activity | 9 | 568 | 31 | 4324 | 1.51E-02 |
| GO:0004691 | cAMP-dependent protein kinase activity | 2 | 568 | 2 | 4324 | 1.72E-02 |
| GO:0004690 | cyclic nucleotide-dependent protein kinase activity | 2 | 568 | 2 | 4324 | 1.72E-02 |
| GO:0008398 | sterol 14-demethylase activity | 2 | 568 | 2 | 4324 | 1.72E-02 |
| GO:0008177 | succinate dehydrogenase (ubiquinone) activity | 2 | 568 | 2 | 4324 | 1.72E-02 |
| GO:0008097 | 5S rRNA binding | 2 | 568 | 2 | 4324 | 1.72E-02 |
| GO:0004739 | pyruvate dehydrogenase (acetyl-transferring) activity | 2 | 568 | 2 | 4324 | 1.72E-02 |
| GO:0016635 | oxidoreductase activity, acting on the CH-CH group of donors, quinone or related compound as acceptor | 2 | 568 | 2 | 4324 | 1.72E-02 |
| GO:0000246 | delta24(24-1) sterol reductase activity | 2 | 568 | 2 | 4324 | 1.72E-02 |
| GO:0004827 | proline-tRNA ligase activity | 2 | 568 | 2 | 4324 | 1.72E-02 |
| GO:0000339 | RNA cap binding | 2 | 568 | 2 | 4324 | 1.72E-02 |
| GO:0004738 | pyruvate dehydrogenase activity | 2 | 568 | 2 | 4324 | 1.72E-02 |
| GO:0008121 | ubiquinol-cytochrome-c reductase activity | 3 | 568 | 5 | 4324 | 1.84E-02 |
| GO:0016681 | oxidoreductase activity, acting on diphenols and related substances as donors, cytochrome as acceptor | 3 | 568 | 5 | 4324 | 1.84E-02 |
| GO:0003925 | small monomeric GTPase activity | 3 | 568 | 5 | 4324 | 1.84E-02 |
| GO:0016679 | oxidoreductase activity, acting on diphenols and related substances as donors | 3 | 568 | 5 | 4324 | 1.84E-02 |
| GO:0015078 | hydrogen ion transporter activity | 5 | 568 | 13 | 4324 | 2.00E-02 |
| GO:0004722 | protein serine/threonine phosphatase activity | 6 | 568 | 18 | 4324 | 2.30E-02 |
| GO:0016705 | oxidoreductase activity, acting on paired donors, with incorporation or reduction of molecular oxygen | 6 | 568 | 18 | 4324 | 2.30E-02 |
| GO:0008170 | N-methyltransferase activity | 6 | 568 | 18 | 4324 | 2.30E-02 |
| GO:0003676 | nucleic acid binding | 73 | 568 | 448 | 4324 | 2.40E-02 |
| GO:0005198 | structural molecule activity | 31 | 568 | 168 | 4324 | 2.86E-02 |
| GO:0016709 | oxidoreductase activity, acting on paired donors, with incorporation or reduction of molecular oxygen, NAD or NADH as one donor, and incorporation of one atom of oxygen | 3 | 568 | 6 | 4324 | 3.32E-02 |
| GO:0008233 | peptidase activity | 23 | 568 | 119 | 4324 | 3.41E-02 |
| GO:0008757 | S-adenosylmethionine-dependent methyltransferase activity | 12 | 568 | 53 | 4324 | 3.86E-02 |
| GO:0016251 | general RNA polymerase II transcription factor activity | 9 | 568 | 36 | 4324 | 3.89E-02 |
| GO:0019202 | amino acid kinase activity | 2 | 568 | 3 | 4324 | 4.72E-02 |
| GO:0000248 | C-5 sterol desaturase activity | 2 | 568 | 3 | 4324 | 4.72E-02 |
| GO:0016661 | oxidoreductase activity, acting on other nitrogenous compounds as donors | 2 | 568 | 3 | 4324 | 4.72E-02 |
| GO:0000171 | ribonuclease MRP activity | 2 | 568 | 3 | 4324 | 4.72E-02 |
| GO:0047956 | glycerol dehydrogenase (NADP+) activity | 2 | 568 | 3 | 4324 | 4.72E-02 |
| GO:0005294 | neutral L-amino acid porter activity | 2 | 568 | 3 | 4324 | 4.72E-02 |
| GO:0000102 | L-methionine porter activity | 2 | 568 | 3 | 4324 | 4.72E-02 |
| GO:0019206 | nucleoside kinase activity | 2 | 568 | 3 | 4324 | 4.72E-02 |
| GO:0004526 | ribonuclease P activity | 2 | 568 | 3 | 4324 | 4.72E-02 |
| GO:0000049 | tRNA binding | 2 | 568 | 3 | 4324 | 4.72E-02 |
| GO:0016624 | oxidoreductase activity, acting on the aldehyde or oxo group of donors, disulfide as acceptor | 2 | 568 | 3 | 4324 | 4.72E-02 |
| GO:0016741 | transferase activity, transferring one-carbon groups | 15 | 568 | 73 | 4324 | 4.91E-02 |
| GO:0004674 | protein serine/threonine kinase activity | 11 | 568 | 49 | 4324 | 4.91E-02 |
| GO:0016455 | RNA polymerase II transcription mediator activity | 3 | 568 | 7 | 4324 | 5.25E-02 |
| GO:0015077 | monovalent inorganic cation transporter activity | 5 | 568 | 17 | 4324 | 6.17E-02 |
| GO:0003674 | molecular_function | 549 | 568 | 4124 | 4324 | 6.91E-02 |
| GO:0008168 | methyltransferase activity | 14 | 568 | 71 | 4324 | 7.50E-02 |
| GO:0042054 | histone methyltransferase activity | 3 | 568 | 8 | 4324 | 7.60E-02 |
| GO:0019205 | nucleobase, nucleoside, nucleotide kinase activity | 3 | 568 | 8 | 4324 | 7.60E-02 |
| GO:0016491 | oxidoreductase activity | 68 | 568 | 441 | 4324 | 7.92E-02 |
| GO:0016215 | CoA desaturase activity | 2 | 568 | 4 | 4324 | 8.62E-02 |
| GO:0015191 | L-methionine transporter activity | 2 | 568 | 4 | 4324 | 8.62E-02 |
| GO:0004768 | stearoyl-CoA 9-desaturase activity | 2 | 568 | 4 | 4324 | 8.62E-02 |
| GO:0030276 | clathrin binding | 2 | 568 | 4 | 4324 | 8.62E-02 |
| GO:0005310 | dicarboxylic acid transporter activity | 2 | 568 | 4 | 4324 | 8.62E-02 |
| GO:0004128 | cytochrome-b5 reductase activity | 2 | 568 | 4 | 4324 | 8.62E-02 |
| GO:0004721 | phosphoprotein phosphatase activity | 7 | 568 | 30 | 4324 | 8.89E-02 |
| GO:0016830 | carbon-carbon lyase activity | 8 | 568 | 36 | 4324 | 9.07E-02 |
| GO:0004497 | monooxygenase activity | 6 | 568 | 25 | 4324 | 9.98E-02 |
| **Cellular component** | |  |  |  |  |  |
| GO:0044429 | mitochondrial part | 65 | 526 | 222 | 3787 | 5.61E-10 |
| GO:0031974 | membrane-enclosed lumen | 96 | 526 | 395 | 3787 | 2.80E-09 |
| GO:0043233 | organelle lumen | 96 | 526 | 395 | 3787 | 2.80E-09 |
| GO:0043234 | protein complex | 149 | 526 | 711 | 3787 | 4.92E-09 |
| GO:0005730 | nucleolus | 40 | 526 | 130 | 3787 | 3.45E-07 |
| GO:0005739 | mitochondrion | 95 | 526 | 426 | 3787 | 3.46E-07 |
| GO:0030529 | ribonucleoprotein complex | 62 | 526 | 243 | 3787 | 4.41E-07 |
| GO:0043228 | non-membrane-bound organelle | 96 | 526 | 442 | 3787 | 1.10E-06 |
| GO:0043232 | intracellular non-membrane-bound organelle | 96 | 526 | 442 | 3787 | 1.10E-06 |
| GO:0005759 | mitochondrial matrix | 31 | 526 | 93 | 3787 | 1.12E-06 |
| GO:0031980 | mitochondrial lumen | 31 | 526 | 93 | 3787 | 1.12E-06 |
| GO:0005852 | eukaryotic translation initiation factor 3 complex | 9 | 526 | 12 | 3787 | 2.69E-06 |
| GO:0031966 | mitochondrial membrane | 31 | 526 | 97 | 3787 | 3.10E-06 |
| GO:0044455 | mitochondrial membrane part | 13 | 526 | 24 | 3787 | 3.58E-06 |
| GO:0005622 | intracellular | 441 | 526 | 2891 | 3787 | 4.20E-06 |
| GO:0044424 | intracellular part | 438 | 526 | 2868 | 3787 | 4.65E-06 |
| GO:0044452 | nucleolar part | 17 | 526 | 39 | 3787 | 5.45E-06 |
| GO:0005761 | mitochondrial ribosome | 17 | 526 | 41 | 3787 | 1.23E-05 |
| GO:0005840 | ribosome | 40 | 526 | 151 | 3787 | 2.18E-05 |
| GO:0000313 | organellar ribosome | 17 | 526 | 43 | 3787 | 2.62E-05 |
| GO:0005746 | mitochondrial electron transport chain | 8 | 526 | 12 | 3787 | 3.90E-05 |
| GO:0005743 | mitochondrial inner membrane | 25 | 526 | 80 | 3787 | 4.36E-05 |
| GO:0019866 | organelle inner membrane | 25 | 526 | 82 | 3787 | 6.90E-05 |
| GO:0031981 | nuclear lumen | 60 | 526 | 270 | 3787 | 7.50E-05 |
| GO:0005740 | mitochondrial envelope | 34 | 526 | 129 | 3787 | 1.02E-04 |
| GO:0005732 | small nucleolar ribonucleoprotein complex | 10 | 526 | 23 | 3787 | 5.12E-04 |
| GO:0016282 | eukaryotic 43S preinitiation complex | 15 | 526 | 47 | 3787 | 1.17E-03 |
| GO:0005844 | polysome | 4 | 526 | 5 | 3787 | 1.64E-03 |
| GO:0045285 | ubiquinol-cytochrome-c reductase complex | 4 | 526 | 5 | 3787 | 1.64E-03 |
| GO:0005750 | respiratory chain complex III (sensu Eukaryota) | 4 | 526 | 5 | 3787 | 1.64E-03 |
| GO:0045275 | respiratory chain complex III | 4 | 526 | 5 | 3787 | 1.64E-03 |
|  | proteasome regulatory particle, lid subcomplex (sensu Eukaryota) | 5 | 526 | 8 | 3787 | 1.98E-03 |
| GO:0031967 | organelle envelope | 37 | 526 | 167 | 3787 | 1.99E-03 |
| GO:0031975 | envelope | 37 | 526 | 167 | 3787 | 1.99E-03 |
| GO:0005956 | protein kinase CK2 complex | 3 | 526 | 3 | 3787 | 2.67E-03 |
| GO:0005762 | mitochondrial large ribosomal subunit | 9 | 526 | 24 | 3787 | 3.37E-03 |
| GO:0044422 | organelle part | 177 | 526 | 1086 | 3787 | 4.19E-03 |
| GO:0044446 | intracellular organelle part | 177 | 526 | 1086 | 3787 | 4.19E-03 |
| GO:0000315 | organellar large ribosomal subunit | 9 | 526 | 25 | 3787 | 4.63E-03 |
| GO:0005737 | cytoplasm | 306 | 526 | 2010 | 3787 | 6.51E-03 |
| GO:0005763 | mitochondrial small ribosomal subunit | 6 | 526 | 14 | 3787 | 7.79E-03 |
| GO:0043229 | intracellular organelle | 335 | 526 | 2228 | 3787 | 8.16E-03 |
| GO:0043226 | organelle | 335 | 526 | 2228 | 3787 | 8.16E-03 |
| GO:0045254 | pyruvate dehydrogenase complex | 3 | 526 | 4 | 3787 | 9.56E-03 |
| GO:0000314 | organellar small ribosomal subunit | 6 | 526 | 15 | 3787 | 1.15E-02 |
| GO:0015934 | large ribosomal subunit | 16 | 526 | 65 | 3787 | 1.39E-02 |
| GO:0005845 | mRNA cap complex | 2 | 526 | 2 | 3787 | 1.93E-02 |
| GO:0005749 | respiratory chain complex II (sensu Eukaryota) | 2 | 526 | 2 | 3787 | 1.93E-02 |
| GO:0045283 | fumarate reductase complex | 2 | 526 | 2 | 3787 | 1.93E-02 |
| GO:0045273 | respiratory chain complex II | 2 | 526 | 2 | 3787 | 1.93E-02 |
| GO:0045257 | succinate dehydrogenase complex (ubiquinone) | 2 | 526 | 2 | 3787 | 1.93E-02 |
| GO:0045281 | succinate dehydrogenase complex | 2 | 526 | 2 | 3787 | 1.93E-02 |
| GO:0044464 | cell part | 490 | 526 | 3436 | 3787 | 2.07E-02 |
| GO:0005742 | mitochondrial outer membrane translocase complex | 3 | 526 | 5 | 3787 | 2.14E-02 |
| GO:0005741 | mitochondrial outer membrane | 6 | 526 | 17 | 3787 | 2.23E-02 |
| GO:0044444 | cytoplasmic part | 194 | 526 | 1254 | 3787 | 2.75E-02 |
| GO:0031968 | organelle outer membrane | 6 | 526 | 18 | 3787 | 2.96E-02 |
| GO:0019867 | outer membrane | 6 | 526 | 18 | 3787 | 2.96E-02 |
| GO:0005623 | cell | 490 | 526 | 3444 | 3787 | 3.10E-02 |
| GO:0015935 | small ribosomal subunit | 12 | 526 | 50 | 3787 | 3.71E-02 |
|  | proteasome core complex, beta-subunit complex (sensu Eukaryota) | 3 | 526 | 6 | 3787 | 3.85E-02 |
| GO:0030663 | COPI coated vesicle membrane | 3 | 526 | 6 | 3787 | 3.85E-02 |
| GO:0030126 | COPI vesicle coat | 3 | 526 | 6 | 3787 | 3.85E-02 |
| GO:0005736 | DNA-directed RNA polymerase I complex | 4 | 526 | 10 | 3787 | 3.87E-02 |
| GO:0000502 | proteasome complex (sensu Eukaryota) | 8 | 526 | 29 | 3787 | 3.91E-02 |
| GO:0030677 | ribonuclease P complex | 2 | 526 | 3 | 3787 | 5.25E-02 |
| GO:0000172 | ribonuclease MRP complex | 2 | 526 | 3 | 3787 | 5.25E-02 |
|  | proteasome regulatory particle, base subcomplex (sensu Eukaryota) | 2 | 526 | 3 | 3787 | 5.25E-02 |
| GO:0005655 | nucleolar ribonuclease P complex | 2 | 526 | 3 | 3787 | 5.25E-02 |
| GO:0012506 | vesicle membrane | 6 | 526 | 22 | 3787 | 7.36E-02 |
| GO:0030120 | vesicle coat | 6 | 526 | 22 | 3787 | 7.36E-02 |
| GO:0030659 | cytoplasmic vesicle membrane | 6 | 526 | 22 | 3787 | 7.36E-02 |
| GO:0030662 | coated vesicle membrane | 6 | 526 | 22 | 3787 | 7.36E-02 |
| GO:0043231 | intracellular membrane-bound organelle | 301 | 526 | 2061 | 3787 | 8.95E-02 |
| GO:0043227 | membrane-bound organelle | 301 | 526 | 2061 | 3787 | 8.95E-02 |
| GO:0031090 | organelle membrane | 51 | 526 | 307 | 3787 | 9.01E-02 |
| GO:0005839 | proteasome core complex (sensu Eukaryota) | 4 | 526 | 13 | 3787 | 9.42E-02 |
| GO:0019897 | extrinsic to plasma membrane | 4 | 526 | 13 | 3787 | 9.42E-02 |
| GO:0012510 | trans-Golgi network transport vesicle membrane | 2 | 526 | 4 | 3787 | 9.53E-02 |
| GO:0030130 | clathrin coat of trans-Golgi network vesicle | 2 | 526 | 4 | 3787 | 9.53E-02 |
| GO:0030121 | AP-1 adaptor complex | 2 | 526 | 4 | 3787 | 9.53E-02 |

Over-represented Gene Ontology (GO) terms amongst genes having decreased transcript abundance.

| **GO ID** | **GO Term** | **List hits** | **List size** | **Pop.hits** | **Pop. Size** | ***p value*** |
| --- | --- | --- | --- | --- | --- | --- |
| **Biological process** | |  |  |  |  |  |
| GO:0019538 | protein metabolism | 174 | 561 | 781 | 4219 | 7.35E-15 |
| GO:0044267 | cellular protein metabolism | 159 | 561 | 695 | 4219 | 1.52E-14 |
| GO:0044260 | cellular macromolecule metabolism | 168 | 561 | 775 | 4219 | 4.09E-13 |
| GO:0043170 | macromolecule metabolism | 254 | 561 | 1383 | 4219 | 2.27E-11 |
| GO:0042254 | ribosome biogenesis and assembly | 51 | 561 | 161 | 4219 | 5.56E-10 |
| GO:0007028 | cytoplasm organization and biogenesis | 51 | 561 | 161 | 4219 | 5.56E-10 |
| GO:0044237 | cellular metabolism | 366 | 561 | 2251 | 4219 | 6.51E-10 |
| GO:0007046 | ribosome biogenesis | 46 | 561 | 140 | 4219 | 1.08E-09 |
| GO:0016072 | rRNA metabolism | 41 | 561 | 118 | 4219 | 1.37E-09 |
| GO:0006364 | rRNA processing | 40 | 561 | 115 | 4219 | 2.12E-09 |
| GO:0044238 | primary metabolism | 334 | 561 | 2028 | 4219 | 3.30E-09 |
| GO:0006457 | protein folding | 23 | 561 | 50 | 4219 | 1.48E-08 |
| GO:0006412 | protein biosynthesis | 72 | 561 | 302 | 4219 | 1.63E-07 |
| GO:0008152 | metabolism | 394 | 561 | 2570 | 4219 | 5.28E-07 |
| GO:0043037 | translation | 30 | 561 | 96 | 4219 | 3.12E-06 |
| GO:0016070 | RNA metabolism | 58 | 561 | 247 | 4219 | 4.91E-06 |
| GO:0009059 | macromolecule biosynthesis | 74 | 561 | 346 | 4219 | 9.30E-06 |
| GO:0006365 | 35S primary transcript processing | 18 | 561 | 46 | 4219 | 9.78E-06 |
| GO:0006413 | translational initiation | 17 | 561 | 42 | 4219 | 1.01E-05 |
| 166 | RNA processing | 43 | 561 | 175 | 4219 | 2.76E-05 |
| GO:0030490 | processing of 20S pre-rRNA | 15 | 561 | 37 | 4219 | 3.32E-05 |
| GO:0006996 | organelle organization and biogenesis | 100 | 561 | 522 | 4219 | 3.55E-05 |
| GO:0009058 | biosynthesis | 123 | 561 | 696 | 4219 | 1.91E-04 |
|  | mitochondrial electron transport, ubiquinol to cytochrome c | 5 | 561 | 6 | 4219 | 2.19E-04 |
| GO:0001302 | replicative cell aging | 6 | 561 | 9 | 4219 | 3.17E-04 |
| GO:0000027 | ribosomal large subunit assembly and maintenance | 11 | 561 | 28 | 4219 | 5.25E-04 |
| GO:0007569 | cell aging | 6 | 561 | 11 | 4219 | 1.38E-03 |
| GO:0006359 | regulation of transcription from RNA polymerase III promoter | 4 | 561 | 5 | 4219 | 1.39E-03 |
| GO:0006360 | transcription from RNA polymerase I promoter | 7 | 561 | 15 | 4219 | 1.73E-03 |
| GO:0009987 | cellular process | 461 | 561 | 3265 | 4219 | 1.78E-03 |
| GO:0043283 | biopolymer metabolism | 151 | 561 | 930 | 4219 | 1.96E-03 |
| GO:0042257 | ribosomal subunit assembly | 12 | 561 | 37 | 4219 | 2.13E-03 |
| GO:0000128 | flocculation | 3 | 561 | 3 | 4219 | 2.34E-03 |
| GO:0006356 | regulation of transcription from RNA polymerase I promoter | 3 | 561 | 3 | 4219 | 2.34E-03 |
| GO:0044249 | cellular biosynthesis | 105 | 561 | 616 | 4219 | 2.34E-03 |
| GO:0042255 | ribosome assembly | 13 | 561 | 43 | 4219 | 2.85E-03 |
| GO:0043412 | biopolymer modification | 73 | 561 | 406 | 4219 | 2.95E-03 |
| GO:0050875 | cellular physiological process | 454 | 561 | 3222 | 4219 | 3.23E-03 |
| GO:0007568 | aging | 6 | 561 | 13 | 4219 | 4.03E-03 |
| GO:0006783 | heme biosynthesis | 6 | 561 | 13 | 4219 | 4.03E-03 |
| GO:0042168 | heme metabolism | 6 | 561 | 13 | 4219 | 4.03E-03 |
| GO:0000154 | rRNA modification | 6 | 561 | 13 | 4219 | 4.03E-03 |
| GO:0006628 | mitochondrial translocation | 7 | 561 | 17 | 4219 | 4.12E-03 |
| GO:0006508 | proteolysis | 27 | 561 | 125 | 4219 | 6.25E-03 |
| GO:0006778 | porphyrin metabolism | 6 | 561 | 14 | 4219 | 6.28E-03 |
| GO:0006779 | porphyrin biosynthesis | 6 | 561 | 14 | 4219 | 6.28E-03 |
| GO:0006626 | protein targeting to mitochondrion | 6 | 561 | 14 | 4219 | 6.28E-03 |
| GO:0006461 | protein complex assembly | 19 | 561 | 79 | 4219 | 6.30E-03 |
| GO:0016337 | cell-cell adhesion | 4 | 561 | 7 | 4219 | 7.77E-03 |
| GO:0009060 | aerobic respiration | 13 | 561 | 48 | 4219 | 8.02E-03 |
| GO:0045333 | cellular respiration | 13 | 561 | 48 | 4219 | 8.02E-03 |
| GO:0016043 | cell organization and biogenesis | 135 | 561 | 849 | 4219 | 8.05E-03 |
| GO:0006696 | ergosterol biosynthesis | 11 | 561 | 38 | 4219 | 8.45E-03 |
| GO:0008204 | ergosterol metabolism | 11 | 561 | 38 | 4219 | 8.45E-03 |
| GO:0007155 | cell adhesion | 5 | 561 | 11 | 4219 | 9.46E-03 |
| GO:0042273 | ribosomal large subunit biogenesis | 5 | 561 | 11 | 4219 | 9.46E-03 |
| GO:0018193 | peptidyl-amino acid modification | 5 | 561 | 11 | 4219 | 9.46E-03 |
| GO:0006091 | generation of precursor metabolites and energy | 32 | 561 | 161 | 4219 | 1.11E-02 |
| GO:0006464 | protein modification | 64 | 561 | 373 | 4219 | 1.52E-02 |
|  | mitochondrial electron transport, succinate to ubiquinone | 2 | 561 | 2 | 4219 | 1.77E-02 |
| GO:0042126 | nitrate metabolism | 2 | 561 | 2 | 4219 | 1.77E-02 |
| GO:0042128 | nitrate assimilation | 2 | 561 | 2 | 4219 | 1.77E-02 |
| GO:0044257 | cellular protein catabolism | 20 | 561 | 93 | 4219 | 1.81E-02 |
| GO:0046148 | pigment biosynthesis | 7 | 561 | 22 | 4219 | 1.99E-02 |
| GO:0042440 | pigment metabolism | 7 | 561 | 22 | 4219 | 1.99E-02 |
| GO:0009451 | RNA modification | 9 | 561 | 32 | 4219 | 2.01E-02 |
| GO:0051603 | proteolysis during cellular protein catabolism | 19 | 561 | 88 | 4219 | 2.01E-02 |
| GO:0016126 | sterol biosynthesis | 11 | 561 | 43 | 4219 | 2.18E-02 |
| GO:0015980 | energy derivation by oxidation of organic compounds | 24 | 561 | 120 | 4219 | 2.41E-02 |
| GO:0006383 | transcription from RNA polymerase III promoter | 7 | 561 | 24 | 4219 | 3.20E-02 |
| GO:0019941 | modification-dependent protein catabolism | 18 | 561 | 87 | 4219 | 3.47E-02 |
| GO:0043632 | modification-dependent macromolecule catabolism | 18 | 561 | 87 | 4219 | 3.47E-02 |
| GO:0000002 | mitochondrial genome maintenance | 5 | 561 | 15 | 4219 | 3.91E-02 |
| GO:0000283 | establishment of cell polarity (sensu Saccharomyces) | 5 | 561 | 15 | 4219 | 3.91E-02 |
| GO:0016125 | sterol metabolism | 11 | 561 | 47 | 4219 | 4.04E-02 |
| GO:0006139 | nucleobase, nucleoside, nucleotide and nucleic acid metabolism | 127 | 561 | 839 | 4219 | 4.63E-02 |
| GO:0006694 | steroid biosynthesis | 11 | 561 | 48 | 4219 | 4.64E-02 |
| GO:0030163 | protein catabolism | 21 | 561 | 109 | 4219 | 4.80E-02 |
| GO:0042798 | protein neddylation during NEDD8 class-dependent protein catabolism | 2 | 561 | 3 | 4219 | 4.83E-02 |
| GO:0045041 | protein import into mitochondrial intermembrane space | 2 | 561 | 3 | 4219 | 4.83E-02 |
| GO:0018195 | peptidyl-arginine modification | 2 | 561 | 3 | 4219 | 4.83E-02 |
| GO:0019942 | NEDD8 class-dependent protein catabolism | 2 | 561 | 3 | 4219 | 4.83E-02 |
| GO:0006534 | cysteine metabolism | 2 | 561 | 3 | 4219 | 4.83E-02 |
| GO:0016574 | histone ubiquitination | 2 | 561 | 3 | 4219 | 4.83E-02 |
| GO:0006549 | isoleucine metabolism | 2 | 561 | 3 | 4219 | 4.83E-02 |
| GO:0006839 | mitochondrial transport | 6 | 561 | 21 | 4219 | 5.04E-02 |
| GO:0006512 | ubiquitin cycle | 22 | 561 | 116 | 4219 | 5.09E-02 |
| GO:0006511 | ubiquitin-dependent protein catabolism | 17 | 561 | 85 | 4219 | 5.23E-02 |
| GO:0043285 | biopolymer catabolism | 31 | 561 | 177 | 4219 | 6.14E-02 |
| 137 | tRNA aminoacylation | 6 | 561 | 22 | 4219 | 6.18E-02 |
| GO:0006418 | tRNA aminoacylation for protein translation | 6 | 561 | 22 | 4219 | 6.18E-02 |
| GO:0006403 | RNA localization | 8 | 561 | 33 | 4219 | 6.25E-02 |
| GO:0046112 | nucleobase biosynthesis | 4 | 561 | 12 | 4219 | 6.38E-02 |
| GO:0006399 | tRNA metabolism | 11 | 561 | 52 | 4219 | 7.62E-02 |
| GO:0008202 | steroid metabolism | 11 | 561 | 52 | 4219 | 7.62E-02 |
| GO:0044265 | cellular macromolecule catabolism | 31 | 561 | 181 | 4219 | 7.83E-02 |
| GO:0019856 | pyrimidine base biosynthesis | 3 | 561 | 8 | 4219 | 7.83E-02 |
| GO:0006743 | ubiquinone metabolism | 3 | 561 | 8 | 4219 | 7.83E-02 |
| GO:0016571 | histone methylation | 3 | 561 | 8 | 4219 | 7.83E-02 |
| GO:0043414 | biopolymer methylation | 5 | 561 | 18 | 4219 | 7.99E-02 |
| GO:0032259 | methylation | 5 | 561 | 18 | 4219 | 7.99E-02 |
| GO:0006090 | pyruvate metabolism | 7 | 561 | 29 | 4219 | 8.05E-02 |
| GO:0006730 | one-carbon compound metabolism | 7 | 561 | 29 | 4219 | 8.05E-02 |
| GO:0008151 | cell growth and/or maintenance | 4 | 561 | 13 | 4219 | 8.28E-02 |
| GO:0015931 | nucleobase, nucleoside, nucleotide and nucleic acid transport | 8 | 561 | 35 | 4219 | 8.38E-02 |
| GO:0007005 | mitochondrion organization and biogenesis | 9 | 561 | 41 | 4219 | 8.53E-02 |
| GO:0006080 | mannan metabolism | 2 | 561 | 4 | 4219 | 8.81E-02 |
| GO:0006097 | glyoxylate cycle | 2 | 561 | 4 | 4219 | 8.81E-02 |
| GO:0016074 | snoRNA metabolism | 2 | 561 | 4 | 4219 | 8.81E-02 |
| GO:0006835 | dicarboxylic acid transport | 2 | 561 | 4 | 4219 | 8.81E-02 |
| GO:0046487 | glyoxylate metabolism | 2 | 561 | 4 | 4219 | 8.81E-02 |
|  | deadenylation-dependent decapping | 2 | 561 | 4 | 4219 | 8.81E-02 |
| **Molecular Function** | |  |  |  |  |  |
| GO:0003754 | chaperone activity | 17 | 568 | 35 | 4324 | 3.76E-07 |
| GO:0003743 | translation initiation factor activity | 16 | 568 | 34 | 4324 | 1.43E-06 |
| GO:0008135 | translation factor activity, nucleic acid binding | 20 | 568 | 50 | 4324 | 1.71E-06 |
| GO:0045182 | translation regulator activity | 21 | 568 | 55 | 4324 | 2.32E-06 |
| GO:0016627 | oxidoreductase activity, acting on the CH-CH group of donors | 11 | 568 | 19 | 4324 | 5.11E-06 |
| GO:0030515 | snoRNA binding | 11 | 568 | 21 | 4324 | 1.86E-05 |
| GO:0003723 | RNA binding | 27 | 568 | 100 | 4324 | 1.39E-04 |
| GO:0003735 | structural constituent of ribosome | 28 | 568 | 112 | 4324 | 4.28E-04 |
| GO:0003724 | RNA helicase activity | 13 | 568 | 39 | 4324 | 9.36E-04 |
| GO:0016628 | oxidoreductase activity, acting on the CH-CH group of donors, NAD or NADP as acceptor | 4 | 568 | 5 | 4324 | 1.32E-03 |
| GO:0004680 | casein kinase activity | 4 | 568 | 5 | 4324 | 1.32E-03 |
| GO:0004004 | ATP-dependent RNA helicase activity | 10 | 568 | 27 | 4324 | 1.46E-03 |
| GO:0008186 | RNA-dependent ATPase activity | 10 | 568 | 27 | 4324 | 1.46E-03 |
| GO:0004682 | protein kinase CK2 activity | 3 | 568 | 3 | 4324 | 2.26E-03 |
| GO:0016274 | protein-arginine N-methyltransferase activity | 3 | 568 | 4 | 4324 | 8.14E-03 |
| GO:0016273 | arginine N-methyltransferase activity | 3 | 568 | 4 | 4324 | 8.14E-03 |
| GO:0004176 | ATP-dependent peptidase activity | 3 | 568 | 4 | 4324 | 8.14E-03 |
| GO:0008026 | ATP-dependent helicase activity | 11 | 568 | 40 | 4324 | 1.16E-02 |
| GO:0016876 | ligase activity, forming aminoacyl-tRNA and related compounds | 10 | 568 | 35 | 4324 | 1.20E-02 |
| GO:0016875 | ligase activity, forming carbon-oxygen bonds | 10 | 568 | 35 | 4324 | 1.20E-02 |
| GO:0004812 | aminoacyl-tRNA ligase activity | 10 | 568 | 35 | 4324 | 1.20E-02 |
| GO:0003773 | heat shock protein activity | 4 | 568 | 8 | 4324 | 1.33E-02 |
| GO:0008369 | obsolete molecular function | 38 | 568 | 204 | 4324 | 1.43E-02 |
| GO:0004175 | endopeptidase activity | 13 | 568 | 52 | 4324 | 1.46E-02 |
| GO:0004386 | helicase activity | 16 | 568 | 69 | 4324 | 1.49E-02 |
| GO:0008565 | protein transporter activity | 9 | 568 | 31 | 4324 | 1.51E-02 |
| GO:0004691 | cAMP-dependent protein kinase activity | 2 | 568 | 2 | 4324 | 1.72E-02 |
| GO:0004690 | cyclic nucleotide-dependent protein kinase activity | 2 | 568 | 2 | 4324 | 1.72E-02 |
| GO:0008398 | sterol 14-demethylase activity | 2 | 568 | 2 | 4324 | 1.72E-02 |
| GO:0008177 | succinate dehydrogenase (ubiquinone) activity | 2 | 568 | 2 | 4324 | 1.72E-02 |
| GO:0008097 | 5S rRNA binding | 2 | 568 | 2 | 4324 | 1.72E-02 |
| GO:0004739 | pyruvate dehydrogenase (acetyl-transferring) activity | 2 | 568 | 2 | 4324 | 1.72E-02 |
| GO:0016635 | oxidoreductase activity, acting on the CH-CH group of donors, quinone or related compound as acceptor | 2 | 568 | 2 | 4324 | 1.72E-02 |
| GO:0000246 | delta24(24-1) sterol reductase activity | 2 | 568 | 2 | 4324 | 1.72E-02 |
| GO:0004827 | proline-tRNA ligase activity | 2 | 568 | 2 | 4324 | 1.72E-02 |
| GO:0000339 | RNA cap binding | 2 | 568 | 2 | 4324 | 1.72E-02 |
| GO:0004738 | pyruvate dehydrogenase activity | 2 | 568 | 2 | 4324 | 1.72E-02 |
| GO:0008121 | ubiquinol-cytochrome-c reductase activity | 3 | 568 | 5 | 4324 | 1.84E-02 |
| GO:0016681 | oxidoreductase activity, acting on diphenols and related substances as donors, cytochrome as acceptor | 3 | 568 | 5 | 4324 | 1.84E-02 |
| GO:0003925 | small monomeric GTPase activity | 3 | 568 | 5 | 4324 | 1.84E-02 |
| GO:0016679 | oxidoreductase activity, acting on diphenols and related substances as donors | 3 | 568 | 5 | 4324 | 1.84E-02 |
| GO:0015078 | hydrogen ion transporter activity | 5 | 568 | 13 | 4324 | 2.00E-02 |
| GO:0004722 | protein serine/threonine phosphatase activity | 6 | 568 | 18 | 4324 | 2.30E-02 |
| GO:0016705 | oxidoreductase activity, acting on paired donors, with incorporation or reduction of molecular oxygen | 6 | 568 | 18 | 4324 | 2.30E-02 |
| GO:0008170 | N-methyltransferase activity | 6 | 568 | 18 | 4324 | 2.30E-02 |
| GO:0003676 | nucleic acid binding | 73 | 568 | 448 | 4324 | 2.40E-02 |
| GO:0005198 | structural molecule activity | 31 | 568 | 168 | 4324 | 2.86E-02 |
| GO:0016709 | oxidoreductase activity, acting on paired donors, with incorporation or reduction of molecular oxygen, NAD or NADH as one donor, and incorporation of one atom of oxygen | 3 | 568 | 6 | 4324 | 3.32E-02 |
| GO:0008233 | peptidase activity | 23 | 568 | 119 | 4324 | 3.41E-02 |
| GO:0008757 | S-adenosylmethionine-dependent methyltransferase activity | 12 | 568 | 53 | 4324 | 3.86E-02 |
| GO:0016251 | general RNA polymerase II transcription factor activity | 9 | 568 | 36 | 4324 | 3.89E-02 |
| GO:0019202 | amino acid kinase activity | 2 | 568 | 3 | 4324 | 4.72E-02 |
| GO:0000248 | C-5 sterol desaturase activity | 2 | 568 | 3 | 4324 | 4.72E-02 |
| GO:0016661 | oxidoreductase activity, acting on other nitrogenous compounds as donors | 2 | 568 | 3 | 4324 | 4.72E-02 |
| GO:0000171 | ribonuclease MRP activity | 2 | 568 | 3 | 4324 | 4.72E-02 |
| GO:0047956 | glycerol dehydrogenase (NADP+) activity | 2 | 568 | 3 | 4324 | 4.72E-02 |
| GO:0005294 | neutral L-amino acid porter activity | 2 | 568 | 3 | 4324 | 4.72E-02 |
| GO:0000102 | L-methionine porter activity | 2 | 568 | 3 | 4324 | 4.72E-02 |
| GO:0019206 | nucleoside kinase activity | 2 | 568 | 3 | 4324 | 4.72E-02 |
| GO:0004526 | ribonuclease P activity | 2 | 568 | 3 | 4324 | 4.72E-02 |
| GO:0000049 | tRNA binding | 2 | 568 | 3 | 4324 | 4.72E-02 |
| GO:0016624 | oxidoreductase activity, acting on the aldehyde or oxo group of donors, disulfide as acceptor | 2 | 568 | 3 | 4324 | 4.72E-02 |
| GO:0016741 | transferase activity, transferring one-carbon groups | 15 | 568 | 73 | 4324 | 4.91E-02 |
| GO:0004674 | protein serine/threonine kinase activity | 11 | 568 | 49 | 4324 | 4.91E-02 |
| GO:0016455 | RNA polymerase II transcription mediator activity | 3 | 568 | 7 | 4324 | 5.25E-02 |
| GO:0015077 | monovalent inorganic cation transporter activity | 5 | 568 | 17 | 4324 | 6.17E-02 |
| GO:0003674 | molecular_function | 549 | 568 | 4124 | 4324 | 6.91E-02 |
| GO:0008168 | methyltransferase activity | 14 | 568 | 71 | 4324 | 7.50E-02 |
| GO:0042054 | histone methyltransferase activity | 3 | 568 | 8 | 4324 | 7.60E-02 |
| GO:0019205 | nucleobase, nucleoside, nucleotide kinase activity | 3 | 568 | 8 | 4324 | 7.60E-02 |
| GO:0016491 | oxidoreductase activity | 68 | 568 | 441 | 4324 | 7.92E-02 |
| GO:0016215 | CoA desaturase activity | 2 | 568 | 4 | 4324 | 8.62E-02 |
| GO:0015191 | L-methionine transporter activity | 2 | 568 | 4 | 4324 | 8.62E-02 |
| GO:0004768 | stearoyl-CoA 9-desaturase activity | 2 | 568 | 4 | 4324 | 8.62E-02 |
| GO:0030276 | clathrin binding | 2 | 568 | 4 | 4324 | 8.62E-02 |
| GO:0005310 | dicarboxylic acid transporter activity | 2 | 568 | 4 | 4324 | 8.62E-02 |
| GO:0004128 | cytochrome-b5 reductase activity | 2 | 568 | 4 | 4324 | 8.62E-02 |
| GO:0004721 | phosphoprotein phosphatase activity | 7 | 568 | 30 | 4324 | 8.89E-02 |
| GO:0016830 | carbon-carbon lyase activity | 8 | 568 | 36 | 4324 | 9.07E-02 |
| GO:0004497 | monooxygenase activity | 6 | 568 | 25 | 4324 | 9.98E-02 |
| **Cellular component** | |  |  |  |  |  |
| GO:0044429 | mitochondrial part | 65 | 526 | 222 | 3787 | 5.61E-10 |
| GO:0031974 | membrane-enclosed lumen | 96 | 526 | 395 | 3787 | 2.80E-09 |
| GO:0043233 | organelle lumen | 96 | 526 | 395 | 3787 | 2.80E-09 |
| GO:0043234 | protein complex | 149 | 526 | 711 | 3787 | 4.92E-09 |
| GO:0005730 | nucleolus | 40 | 526 | 130 | 3787 | 3.45E-07 |
| GO:0005739 | mitochondrion | 95 | 526 | 426 | 3787 | 3.46E-07 |
| GO:0030529 | ribonucleoprotein complex | 62 | 526 | 243 | 3787 | 4.41E-07 |
| GO:0043228 | non-membrane-bound organelle | 96 | 526 | 442 | 3787 | 1.10E-06 |
| GO:0043232 | intracellular non-membrane-bound organelle | 96 | 526 | 442 | 3787 | 1.10E-06 |
| GO:0005759 | mitochondrial matrix | 31 | 526 | 93 | 3787 | 1.12E-06 |
| GO:0031980 | mitochondrial lumen | 31 | 526 | 93 | 3787 | 1.12E-06 |
| GO:0005852 | eukaryotic translation initiation factor 3 complex | 9 | 526 | 12 | 3787 | 2.69E-06 |
| GO:0031966 | mitochondrial membrane | 31 | 526 | 97 | 3787 | 3.10E-06 |
| GO:0044455 | mitochondrial membrane part | 13 | 526 | 24 | 3787 | 3.58E-06 |
| GO:0005622 | intracellular | 441 | 526 | 2891 | 3787 | 4.20E-06 |
| GO:0044424 | intracellular part | 438 | 526 | 2868 | 3787 | 4.65E-06 |
| GO:0044452 | nucleolar part | 17 | 526 | 39 | 3787 | 5.45E-06 |
| GO:0005761 | mitochondrial ribosome | 17 | 526 | 41 | 3787 | 1.23E-05 |
| GO:0005840 | ribosome | 40 | 526 | 151 | 3787 | 2.18E-05 |
| GO:0000313 | organellar ribosome | 17 | 526 | 43 | 3787 | 2.62E-05 |
| GO:0005746 | mitochondrial electron transport chain | 8 | 526 | 12 | 3787 | 3.90E-05 |
| GO:0005743 | mitochondrial inner membrane | 25 | 526 | 80 | 3787 | 4.36E-05 |
| GO:0019866 | organelle inner membrane | 25 | 526 | 82 | 3787 | 6.90E-05 |
| GO:0031981 | nuclear lumen | 60 | 526 | 270 | 3787 | 7.50E-05 |
| GO:0005740 | mitochondrial envelope | 34 | 526 | 129 | 3787 | 1.02E-04 |
| GO:0005732 | small nucleolar ribonucleoprotein complex | 10 | 526 | 23 | 3787 | 5.12E-04 |
| GO:0016282 | eukaryotic 43S preinitiation complex | 15 | 526 | 47 | 3787 | 1.17E-03 |
| GO:0005844 | polysome | 4 | 526 | 5 | 3787 | 1.64E-03 |
| GO:0045285 | ubiquinol-cytochrome-c reductase complex | 4 | 526 | 5 | 3787 | 1.64E-03 |
| GO:0005750 | respiratory chain complex III (sensu Eukaryota) | 4 | 526 | 5 | 3787 | 1.64E-03 |
| GO:0045275 | respiratory chain complex III | 4 | 526 | 5 | 3787 | 1.64E-03 |
|  | proteasome regulatory particle, lid subcomplex (sensu Eukaryota) | 5 | 526 | 8 | 3787 | 1.98E-03 |
| GO:0031967 | organelle envelope | 37 | 526 | 167 | 3787 | 1.99E-03 |
| GO:0031975 | envelope | 37 | 526 | 167 | 3787 | 1.99E-03 |
| GO:0005956 | protein kinase CK2 complex | 3 | 526 | 3 | 3787 | 2.67E-03 |
| GO:0005762 | mitochondrial large ribosomal subunit | 9 | 526 | 24 | 3787 | 3.37E-03 |
| GO:0044422 | organelle part | 177 | 526 | 1086 | 3787 | 4.19E-03 |
| GO:0044446 | intracellular organelle part | 177 | 526 | 1086 | 3787 | 4.19E-03 |
| GO:0000315 | organellar large ribosomal subunit | 9 | 526 | 25 | 3787 | 4.63E-03 |
| GO:0005737 | cytoplasm | 306 | 526 | 2010 | 3787 | 6.51E-03 |
| GO:0005763 | mitochondrial small ribosomal subunit | 6 | 526 | 14 | 3787 | 7.79E-03 |
| GO:0043229 | intracellular organelle | 335 | 526 | 2228 | 3787 | 8.16E-03 |
| GO:0043226 | organelle | 335 | 526 | 2228 | 3787 | 8.16E-03 |
| GO:0045254 | pyruvate dehydrogenase complex | 3 | 526 | 4 | 3787 | 9.56E-03 |
| GO:0000314 | organellar small ribosomal subunit | 6 | 526 | 15 | 3787 | 1.15E-02 |
| GO:0015934 | large ribosomal subunit | 16 | 526 | 65 | 3787 | 1.39E-02 |
| GO:0005845 | mRNA cap complex | 2 | 526 | 2 | 3787 | 1.93E-02 |
| GO:0005749 | respiratory chain complex II (sensu Eukaryota) | 2 | 526 | 2 | 3787 | 1.93E-02 |
| GO:0045283 | fumarate reductase complex | 2 | 526 | 2 | 3787 | 1.93E-02 |
| GO:0045273 | respiratory chain complex II | 2 | 526 | 2 | 3787 | 1.93E-02 |
| GO:0045257 | succinate dehydrogenase complex (ubiquinone) | 2 | 526 | 2 | 3787 | 1.93E-02 |
| GO:0045281 | succinate dehydrogenase complex | 2 | 526 | 2 | 3787 | 1.93E-02 |
| GO:0044464 | cell part | 490 | 526 | 3436 | 3787 | 2.07E-02 |
| GO:0005742 | mitochondrial outer membrane translocase complex | 3 | 526 | 5 | 3787 | 2.14E-02 |
| GO:0005741 | mitochondrial outer membrane | 6 | 526 | 17 | 3787 | 2.23E-02 |
| GO:0044444 | cytoplasmic part | 194 | 526 | 1254 | 3787 | 2.75E-02 |
| GO:0031968 | organelle outer membrane | 6 | 526 | 18 | 3787 | 2.96E-02 |
| GO:0019867 | outer membrane | 6 | 526 | 18 | 3787 | 2.96E-02 |
| GO:0005623 | cell | 490 | 526 | 3444 | 3787 | 3.10E-02 |
| GO:0015935 | small ribosomal subunit | 12 | 526 | 50 | 3787 | 3.71E-02 |
|  | proteasome core complex, beta-subunit complex (sensu Eukaryota) | 3 | 526 | 6 | 3787 | 3.85E-02 |
| GO:0030663 | COPI coated vesicle membrane | 3 | 526 | 6 | 3787 | 3.85E-02 |
| GO:0030126 | COPI vesicle coat | 3 | 526 | 6 | 3787 | 3.85E-02 |
| GO:0005736 | DNA-directed RNA polymerase I complex | 4 | 526 | 10 | 3787 | 3.87E-02 |
| GO:0000502 | proteasome complex (sensu Eukaryota) | 8 | 526 | 29 | 3787 | 3.91E-02 |
| GO:0030677 | ribonuclease P complex | 2 | 526 | 3 | 3787 | 5.25E-02 |
| GO:0000172 | ribonuclease MRP complex | 2 | 526 | 3 | 3787 | 5.25E-02 |
|  | proteasome regulatory particle, base subcomplex (sensu Eukaryota) | 2 | 526 | 3 | 3787 | 5.25E-02 |
| GO:0005655 | nucleolar ribonuclease P complex | 2 | 526 | 3 | 3787 | 5.25E-02 |
| GO:0012506 | vesicle membrane | 6 | 526 | 22 | 3787 | 7.36E-02 |
| GO:0030120 | vesicle coat | 6 | 526 | 22 | 3787 | 7.36E-02 |
| GO:0030659 | cytoplasmic vesicle membrane | 6 | 526 | 22 | 3787 | 7.36E-02 |
| GO:0030662 | coated vesicle membrane | 6 | 526 | 22 | 3787 | 7.36E-02 |
| GO:0043231 | intracellular membrane-bound organelle | 301 | 526 | 2061 | 3787 | 8.95E-02 |
| GO:0043227 | membrane-bound organelle | 301 | 526 | 2061 | 3787 | 8.95E-02 |
| GO:0031090 | organelle membrane | 51 | 526 | 307 | 3787 | 9.01E-02 |
| GO:0005839 | proteasome core complex (sensu Eukaryota) | 4 | 526 | 13 | 3787 | 9.42E-02 |
| GO:0019897 | extrinsic to plasma membrane | 4 | 526 | 13 | 3787 | 9.42E-02 |
| GO:0012510 | trans-Golgi network transport vesicle membrane | 2 | 526 | 4 | 3787 | 9.53E-02 |
| GO:0030130 | clathrin coat of trans-Golgi network vesicle | 2 | 526 | 4 | 3787 | 9.53E-02 |
| GO:0030121 | AP-1 adaptor complex | 2 | 526 | 4 | 3787 | 9.53E-02 |
