# Supplementary material for: Sub-Telomere Directed Gene Expression during Initiation of Invasive Aspergillosis
Source: PLoS Pathog. 2008 Sep 12;4(9):e1000154. doi: 10.1371/journal.ppat.1000154 (PMC2526178; doi:10.1371/journal.ppat.1000154)
Supplement: Table S4 — TOR analysis (0.37 MB DOC) [file ppat.1000154.s007.doc]

**Table S3**

***A. fumigatus* genes having increased transcript abundance, relative to laboratory culture, in the murine lung and *S. cerevisiae* orthologs regulated by rapamycin-mediated TOR kinase inhibition**

| **Accession** | **Yeast ortholog** | **Yeast ortholog description** | | |  |  |  |  |  |  |  |  |  |  |  |  |  |  |  |  |  |  |  |  |  |  |  |  |  |  |  |
| --- | --- | --- | --- | --- | --- | --- | --- | --- | --- | --- | --- | --- | --- | --- | --- | --- | --- | --- | --- | --- | --- | --- | --- | --- | --- | --- | --- | --- | --- | --- | --- |
| **Afu4g13660** | **YBR043C** | **Multidrug transporter required for resistance to quinidine, barban, cisplatin, and bleomycin** | | | | | | | | | | | | | | |  |  |  |  |  |  |  |  |  |  |  |  |  |  |  |
| Afu1g15520 | YBR208C | Urea amidolyase, contains both urea carboxylase and allophanate hydrolase activities, degrades urea to CO2 and NH3; expression sensitive to nitrogen catabolite repression. | | | | | | | | | | | | | | | | | | |  |  |  |  |  |  |  |  |  |  |  |
| Afu7g06380 | YBR299W | Maltase (alpha-D-glucosidase), inducible protein involved in maltose catabolism; encoded in the MAL3 complex locus; functional in genomic reference strain S288C | | | | | | | | | | | | |  |  |  |  |  |  |  |  |  |  |  |  |  |  |  |  |  |
| Afu1g06150 | YCL064C | Catabolic L-serine (L-threonine) deaminase, catalyzes the degradation of both L-serine and L-threonine; required to use serine or threonine as the sole nitrogen source. | | | | | | | | | | | | | | | | |  |  |  |  |  |  |  |  |  |  |  |  |  |
| Afu1g10780 | YDR019C | T subunit of the mitochondrial glycine decarboxylase complex, required for the catabolism of glycine to 5,10-methylene-THF. | | | | | | | | | | | | | | | |  |  |  |  |  |  |  |  |  |  |  |  |  |  |
| **Afu2g04260** | **YDR421W** | **Zinc finger transcriptional activator of the Zn2Cys6 family; activates transcription of aromatic amino acid catabolic genes in the presence of aromatic amino acids** | | | | | | | | | | | | |  |  |  |  |  |  |  |  |  |  |  |  |  |  |  |  |  |
| Afu7g06060 | YEL065W | Ferrioxamine B transporter, member of the ARN family of transporters that specifically recognize siderophore-iron chelates; transcription is induced during iron deprivation. | | | | | | | | | | | | | | | | | |  |  |  |  |  |  |  |  |  |  |  |  |
| Afu1g17370 | YFL014W | Plasma membrane localized protein that protects membranes from desiccation; induced by heat shock, oxidative stress, osmostress, stationary phase entry, glucose depletion. | | | | | | | | | | | | | | | | | | |  |  |  |  |  |  |  |  |  |  |  |
| Afu5g08090 | YFL059W | Member of a stationary phase-induced gene family; transcription of SNZ2 is induced prior to diauxic shift, and also in the absence of thiamin in a Thi2p-dependent manner;. | | | | | | | | | | | | | | | | |  |  |  |  |  |  |  |  |  |  |  |  |  |
| Afu1g17150 | YGR288W | MAL-activator protein, part of complex locus MAL1; nonfunctional in genomic reference strain S288C | | | | | | | |  |  |  |  |  |  |  |  |  |  |  |  |  |  |  |  |  |  |  |  |  |  |
| Afu3g07850 | YHR028C | Dipeptidyl aminopeptidase, synthesized as a glycosylated precursor; localizes to the vacuolar membrane; similar to Ste13p | | | | | | | | | |  |  |  |  |  |  |  |  |  |  |  |  |  |  |  |  |  |  |  |  |
| Afu5g13810 | YHR112C | Putative protein of unknown function; green fluorescent protein (GFP)-fusion protein localizes to the cytoplasm | | | | | | | | |  |  |  |  |  |  |  |  |  |  |  |  |  |  |  |  |  |  |  |  |  |
| Afu5g01520 | YIL121W | Multidrug transporter required for resistance to quinidine, barban, cisplatin, and bleomycin; may have a role in potassium uptake; member of the major facilitator superfamily. | | | | | | | | | | | | | | | | | |  |  |  |  |  |  |  |  |  |  |  |  |
| Afu6g03540 | YIR031C | Malate synthase, role in allantoin degradation unknown; expression sensitive to nitrogen catabolite repression and induced by allophanate, an intermediate in allantoin degradation | | | | | | | | | | | | | |  |  |  |  |  |  |  |  |  |  |  |  |  |  |  |  |
| Afu8g04760 | YIR032C | Ureidoglycolate hydrolase, converts ureidoglycolate to glyoxylate and urea in the third step of allantoin degradation; expression sensitive to nitrogen catabolite repression | | | | | | | | | | | | |  |  |  |  |  |  |  |  |  |  |  |  |  |  |  |  |  |
| Afu2g17300 | YIR038C | ER associated glutathione S-transferase capable of homodimerization; expression induced during the diauxic shift and throughout stationary phase. | | | | | | | | | | | | | | |  |  |  |  |  |  |  |  |  |  |  |  |  |  |  |
| Afu7g00850 | YIR042C | Putative protein of unknown function; YIR042C is a non-essential gene | | | | | |  |  |  |  |  |  |  |  |  |  |  |  |  |  |  |  |  |  |  |  |  |  |  |  |
| Afu1g09690 | YJL087C | tRNA ligase, required for tRNA splicing; composed of three essential domains containing the phosphodiesterase, polynucleotide kinase, and ligase activities. | | | | | | | | | | | | | | | | | |  |  |  |  |  |  |  |  |  |  |  |  |
| **Afu7g04290** | **YKR039W** | **General amino acid permease; localization to the plasma membrane is regulated by nitrogen source** | | | | | | | |  |  |  |  |  |  |  |  |  |  |  |  |  |  |  |  |  |  |  |  |  |  |
| Afu6g07720 | YKR097W | Phosphoenolpyruvate carboxykinase, key enzyme in gluconeogenesis, catalyzes early reaction in carbohydrate biosynthesis, glucose represses transcription. | | | | | | | | | | | | | | | | | | |  |  |  |  |  |  |  |  |  |  |  |
| Afu1g12620 | YKR105C | Putative transporter of the Major Facilitator Superfamily (MFS) | | | | |  |  |  |  |  |  |  |  |  |  |  |  |  |  |  |  |  |  |  |  |  |  |  |  |  |
| Afu3g02300 | YLR142W | Proline oxidase, nuclear-encoded mitochondrial protein involved in utilization of proline as sole nitrogen source; PUT1 transcription is induced by Put3p in the presence of proline | | | | | | | | | | | | | | | | |  |  |  |  |  |  |  |  |  |  |  |  |  |
| Afu7g04970 | YLR214W | Ferric reductase and cupric reductase, reduces siderophore-bound iron and oxidized copper prior to uptake by transporters; expression induced by low copper and iron levels | | | | | | | | | | | | | |  |  |  |  |  |  |  |  |  |  |  |  |  |  |  |  |
| Afu4g14640 | YMR319C | Low-affinity Fe(II) transporter of the plasma membrane | | | | |  |  |  |  |  |  |  |  |  |  |  |  |  |  |  |  |  |  |  |  |  |  |  |  |  |
| Afu7g04710 | YNL023C | Protein that binds to Fpr1p (FKBP12), conferring rapamycin resistance by competing with rapamycin for Fpr1p binding; has similarity to putative transcription factors. | | | | | | | | | | | | | | | | |  |  |  |  |  |  |  |  |  |  |  |  |  |
| Afu7g00830 | YNR064C | Epoxide hydrolase, member of the alpha/beta hydrolase fold family; may have a role in detoxification of epoxides | | | | | | | | |  |  |  |  |  |  |  |  |  |  |  |  |  |  |  |  |  |  |  |  |  |
| Afu2g02480 | YOL048C | Putative protein of unknown function | | |  |  |  |  |  |  |  |  |  |  |  |  |  |  |  |  |  |  |  |  |  |  |  |  |  |  |  |
| Afu6g08570 | YOR346W | Deoxycytidyl transferase, forms a complex with the subunits of DNA polymerase zeta, Rev3p and Rev7p; involved in repair of abasic sites in damaged DNA | | | | | | | | | | | |  |  |  |  |  |  |  |  |  |  |  |  |  |  |  |  |  |  |
| Afu7g01090 | YOR348C | Proline permease, required for high-affinity transport of proline; also transports the toxic proline analog azetidine-2-carboxylate (AzC);. | | | | | | | | | | | | | | |  |  |  |  |  |  |  |  |  |  |  |  |  |  |  |
| Afu7g01000 | YOR374W | Mitochondrial aldehyde dehydrogenase, required for growth on ethanol and conversion of acetaldehyde to acetate; activity is K+ dependent. | | | | | | | | | | | | | | | | |  |  |  |  |  |  |  |  |  |  |  |  |  |
| Afu1g01600 | YOR386W | DNA photolyase involved in photoreactivation, repairs pyrimidine dimers in the presence of visible light; induced by DNA damage; regulated by transcriptional repressor Rph1p | | | | | | | | | | | | | |  |  |  |  |  |  |  |  |  |  |  |  |  |  |  |  |
| Afu1g04780 | YPL147W | Subunit of a heterodimeric peroxisomal ATP-binding cassette transporter complex (Pxa1p-Pxa2p), required for import of long-chain fatty acids into peroxisomes. | | | | | | | | | | | | | | | | | | |  |  |  |  |  |  |  |  |  |  |  |
| Afu2g08800 | YPL265W | Dicarboxylic amino acid permease, mediates high-affinity and high-capacity transport of L-glutamate and L-aspartate; also a transporter for Gln, Asn, Ser, Ala, and Gly | | | | | | | | | | | | |  |  |  |  |  |  |  |  |  |  |  |  |  |  |  |  |  |
| Afu6g03730 | YPR002W | Mitochondrial protein that participates in respiration, induced by diauxic shift; homologous to E. coli PrpD, may take part in the conversion of 2-methylcitrate to 2-methylisocitrate | | | | | | | | | | | | | |  |  |  |  |  |  |  |  |  |  |  |  |  |  |  |  |
| Afu3g02280 | YPR026W | Acid trehalase required for utilization of extracellular trehalose | | | | |  |  |  |  |  |  |  |  |  |  |  |  |  |  |  |  |  |  |  |  |  |  |  |  |  |
|  |  |  |  |  |  |  |  |  |  |  |  |  |  |  |  |  |  |  |  |  |  |  |  |  |  |  |  |  |  |  |  |

***A. fumigatus* genes having decreased transcript abundance, relative to laboratory culture, in the murine lung and *S. cerevisiae* orthologs regulated by rapamycin-mediated TOR kinase inhibition**

| Afu1g05560 | YAL036C | Member of the DRG family of GTP-binding proteins; interacts with translating ribosomes and with Tma46p | | | | | | | | |  | | | |  |  |  |  |  |  |  |  |  |  |
| --- | --- | --- | --- | --- | --- | --- | --- | --- | --- | --- | --- | --- | --- | --- | --- | --- | --- | --- | --- | --- | --- | --- | --- | --- |
| Afu8g05580 | YBL015W | Acetyl-coA hydrolase, primarily localized to mitochondria; required for acetate utilization and for diploid pseudohyphal growth | | | | | | | | | | | | | |  |  |  |  |  |  |  |  |  |
| Afu1g07470 | YBL078C | Protein required for autophagy; modified by the serial action of Atg4p, Atg7p, and Atg3p, and conjugated at the C terminus with phosphatidylethanolamine,. | | | | | | | | | | | | | | | | | | | | |  |  |
| **Afu1g06190** | **YBR034C** | **Nuclear SAM-dependent mono- and asymmetric arginine dimethylating methyltransferase that modifies hnRNPs, including Npl3p and Hrp1p.** | | | | | | | | | | | | | | | | | | | | |  |  |
| Afu1g14110 | YBR154C | RNA polymerase subunit ABC27, common to RNA polymerases I, II, and III; contacts DNA and affects transactivation | | | | | | | | | | | | |  |  |  |  |  |  |  |  |  |  |
| **Afu6g13600** | **YBR155W** | **TPR-containing co-chaperone; binds both Hsp82p (Hsp90) and Ssa1p (Hsp70) and stimulates the ATPase activity of SSA1, ts mutants reduce Hsp82p function.** | | | | | | | | | | | | | | | | | | | | | | |
| Afu1g09770 | YCL037C | Cytoplasmic RNA-binding protein that associates with translating ribosomes; involved in heme regulation of Hap1p as a component of the HMC complex. | | | | | | | | | | | | | | | | | | | | |  |  |
| Afu5g02760 | YCR034W | Fatty acid elongase, involved in sphingolipid biosynthesis; acts on fatty acids of up to 24 carbons in length; mutations have regulatory effects on 1,3-beta-glucan synthase. | | | | | | | | | | | | | | | | | | | | |  |  |
| Afu5g05610 | YCR063W | Protein involved in bud-site selection; analysis of integrated high-throughput datasets predicts an involvement in RNA splicing. | | | | | | | | | | | | | | | | | | | | |  |  |
| **Afu1g14220** | **YDL014W** | **Nucleolar protein, component of the small subunit processome complex, which is required for processing of pre-18S rRNA; has similarity to mammalia fibrillarin** | | | | | | | | | | | | | | | | |  |  |  |  |  |  |
| Afu1g11170 | YDL154W | Protein of the MutS family, forms a dimer with Msh4p that facilitates crossovers between homologs during meiosis; msh5-Y823H mutation confers tolerance to DNA alkylating agents. | | | | | | | | | | | | | | | | | | | | | |  |
| Afu5g02160 | YDL167C | Protein of unknown function, rich in asparagine residues | |  | |  | | |  | | | |  | |  |  |  |  |  |  |  |  |  |  |
| Afu5g02410 | YDR021W | Nucleolar protein required for maturation of 18S rRNA, member of the eIF4A subfamily of DEAD-box ATP-dependent RNA helicases | | | | | | | | | | | | | |  |  |  |  |  |  |  |  |  |
| Afu1g10310 | YDR091C | Essential iron-sulfur protein required for ribosome biogenesis and translation initiation;. | | | | | | | | | | | | | | | | | | | | | |  |
| Afu2g16820 | YDR101C | Shuttling pre-60S factor; involved in the biogenesis of ribosomal large subunit biogenesis; interacts directly with Alb1; responsible for Tif6 recycling defects in absence of Rei1. | | | | | | | | | | | | | | | | | | | | |  |  |
| Afu6g13630 | YDR124W | Putative protein of unknown function; non-essential gene; expression is strongly induced by alpha factor | | | | | | | | |  | | | |  |  |  |  |  |  |  |  |  |  |
| Afu1g05070 | YDR165W | Subunit of a tRNA methyltransferase complex composed of Trm8p and Trm82p that catalyzes 7-methylguanosine modification of tRNA | | | | | | | | | | | | | | |  |  |  |  |  |  |  |  |
| Afu2g03930 | YDR324C | Nucleolar protein, component of the small subunit (SSU) processome containing the U3 snoRNA that is involved in processing of pre-18S rRNA | | | | | | | | | | | | | | |  |  |  |  |  |  |  |  |
| **Afu5g10540** | **YEL011W** | **Glycogen branching enzyme, involved in glycogen accumulation; green fluorescent protein (GFP)-fusion protein localizes to the cytoplasm in a punctate pattern** | | | | | | | | | | | | | | | | |  |  |  |  |  |  |
| Afu6g09160 | YEL012W | Ubiquitin-conjugating enzyme that negatively regulates gluconeogenesis by mediating the glucose-induced ubiquitination of fructose-1,6-bisphosphatase (FBPase). | | | | | | | | | | | | | | | | | | | | | |  |
| Afu2g08360 | YEL021W | Orotidine-5'-phosphate (OMP) decarboxylase, catalyzes the sixth enzymatic step in the de novo biosynthesis of pyrimidines, converting OMP into uridine monophosphate (UMP) | | | | | | | | | | | | | | | | | | | | | |  |
| Afu4g07580 | YER025W | Gamma subunit of the translation initiation factor eIF2, involved in the identification of the start codon; binds GTP when forming the ternary complex with GTP and tRNAi-Met | | | | | | | | | | | | | | | | |  |  |  |  |  |  |
| Afu5g07020 | YER036C | ATPase of the ATP-binding cassette (ABC) family involved in 40S and 60S ribosome biogenesis, has similarity to Gcn20p. | | | | | | | | | | | | | | | | | | |  |  |  |  |
| **Afu3g13280** | **YFL002C** | **Putative ATP-dependent RNA helicase, nucleolar protein required for synthesis of 60S ribosomal subunits at a late step in the pathway; sediments with 66S pre-ribosomes in sucrose gradients** | | | | | | | | | | | | | | | | | | |  |  |  |  |
| Afu7g05920 | YGL055W | Fatty acid desaturase, required for monounsaturated fatty acid synthesis and for normal distribution of mitochondria | | | | | | | | | | | | |  |  |  |  |  |  |  |  |  |  |
| **Afu2g06310** | **YGL078C** | **Putative ATP-dependent RNA helicase of the DEAD-box family involved in ribosomal biogenesis** | | | | | | | | |  | | | |  |  |  |  |  |  |  |  |  |  |
| Afu5g06700 | YGR123C | Protein serine/threonine phosphatase with similarity to human phosphatase PP5; present in both the nucleus and cytoplasm; expressed during logarithmic growth | | | | | | | | | | | | | | | | |  |  |  |  |  |  |
| Afu2g04780 | YGR145W | Essential nucleolar protein of unknown function; contains WD repeats, interacts with Mpp10p and Bfr2p, and has homology to Spb1p | | | | | | | | | | | | | | |  |  |  |  |  |  |  |  |
| Afu6g11800 | YGR213C | Protein involved in 7-aminocholesterol resistance; has seven potential membrane-spanning regions | | | | | | | | |  | | | |  |  |  |  |  |  |  |  |  |  |
| Afu3g13320 | YGR214W | Protein component of the small (40S) ribosomal subunit, nearly identical to Rps0Bp; required for maturation of 18S rRNA along with Rps0Bp. | | | | | | | | | | | | | | | | | | | | |  |  |
| Afu6g07880 | YHR016C | Protein involved in the organization of the actin cytoskeleton; contains SH3 domain similar to Rvs167p | | | | | | | | |  | | | |  |  |  |  |  |  |  |  |  |  |
| Afu2g16010 | YHR020W | Protein of unknown function that may interact with ribosomes, based on co-purification experiments; has similarity to proline-tRNA ligase; YHR020W is an essential gene | | | | | | | | | | | | | | | | |  |  |  |  |  |  |
| **Afu8g04820** | **YHR062C** | **Subunit of both RNase MRP, which cleaves pre-rRNA, and nuclear RNase P, which cleaves tRNA precursors to generate mature 5' ends** | | | | | | | | | | | | | | |  |  |  |  |  |  |  |  |
| Afu5g13480 | YIL061C | Component of U1 snRNP required for mRNA splicing via spliceosome; may interact with poly(A) polymerase to regulate polyadenylation; homolog of human U1 70K protein | | | | | | | | | | | | | | | | |  |  |  |  |  |  |
| Afu2g12440 | YIL103W | Protein required, along with Dph2p, Kti11p, Jjj3p, and Dph5p, for synthesis of diphthamide, which is a modified histidine residue of translation elongation factor 2 (Eft1p or Eft2p). | | | | | | | | | | | | | | | | | | | | |  |  |
| Afu4g07080 | YIR026C | Protein phosphatase involved in vegetative growth at low temperatures, sporulation, and glycogen accumulation; transcription induced by low temperature and nitrogen starvation;. | | | | | | | | | | | | | | | | | | | | | | |
| **Afu6g11070** | **YJL033W** | **Putative nucleolar DEAD box RNA helicase; high-copy number suppression of a U14 snoRNA processing mutant suggests an involvement in 18S rRNA synthesis** | | | | | | | | | | | | | | | | |  |  |  |  |  |  |
| Afu6g13370 | YJL109C | Nucleolar protein, component of the small subunit (SSU) processome containing the U3 snoRNA that is involved in processing of pre-18S rRNA | | | | | | | | | | | | | | |  |  |  |  |  |  |  |  |
| **Afu6g11310** | **YJL130C** | **Bifunctional carbamoylphosphate synthetase (CPSase)-aspartate transcarbamylase (ATCase), catalyzes the first two enzymatic steps in the de novo biosynthesis of pyrimidines** | | | | | | | | | | | | | | | | | | | | | |  |
| Afu1g06810 | YJL200C | Putative mitochondrial aconitase isozyme; similarity to Aco1p, an aconitase required for the TCA cycle; expression induced during growth on glucose, by amino acid starvation via Gcn4p | | | | | | | | | | | | | | | | | | | | |  |  |
| Afu3g13480 | YJR007W | Alpha subunit of the translation initiation factor eIF2, involved in the identification of the start codon; phosphorylation of Ser51 is required for regulation of translation | | | | | | | | | | | | | | | | | | | |  |  |  |
| Afu1g04070 | YJR047C | Translation initiation factor eIF-5A, promotes formation of the first peptide bond; similar to and functionally redundant with Hyp2. | | | | | | | | | | | | | | | | | | | | |  |  |
| Afu8g01970 | YJR153W | Endo-polygalacturonase, pectolytic enzyme that hydrolyzes the alpha-1,4-glycosidic bonds in the rhamnogalacturonan chains in pectins | | | | | | | | | | | | | | |  |  |  |  |  |  |  |  |
| **Afu5g13470** | **YKL009W** | **Protein involved in mRNA turnover and ribosome assembly, localizes to the nucleolus** | | | | | |  | | | |  | | |  |  |  |  |  |  |  |  |  |  |
| Afu7g02420 | YKL029C | Mitochondrial malic enzyme, catalyzes the oxidative decarboxylation of malate to pyruvate, which is a key intermediate in sugar metabolism and a precursor for synthesis of several amino acids | | | | | | | | | | | | | | | | | | |  |  |  |  |
| Afu6g04570 | YKL081W | Translation elongation factor EF-1 gamma |  | |  | |  | | |  | | | |  |  |  |  |  |  |  |  |  |  |  |
| Afu6g02150 | YKL100C | Putative protein of unknown function with similarity to a human minor histocompatibility antigen; YKL100C is not an essential gene | | | | | | | | | | | | | |  |  |  |  |  |  |  |  |  |
| Afu5g09850 | YKL205W | Nuclear pore protein involved in nuclear export of pre-tRNA | |  | |  | | |  | | | |  | |  |  |  |  |  |  |  |  |  |  |
| **Afu2g02190** | **YLL035W** | **Protein of unknown function, required for cell growth and possibly involved in rRNA processing; mRNA is cell cycle regulated** | | | | | | | | | | | | | |  |  |  |  |  |  |  |  |  |
| **Afu3g12300** | **YLR061W** | **Protein component of the large (60S) ribosomal subunit, has similarity to Rpl22Bp and to rat L22 ribosomal protein** | | | | | | | | | | | | |  |  |  |  |  |  |  |  |  |  |
| Afu1g03930 | YLR067C | Specific translational activator for the COX1 mRNA, also influences stability of intron-containing COX1 primary transcripts; located in the mitochondrial inner membrane | | | | | | | | | | | | | | | | |  |  |  |  |  |  |
| **Afu2g12150** | **YLR106C** | **Huge dynein-related AAA-type ATPase (midasin), forms extended pre-60S particle with the Rix1 complex (Rix1p-Ipi1p-Ipi3p), may mediate ATP-dependent remodeling of 60S subunits** | | | | | | | | | | | | | | | | | | | | | |  |
| Afu3g06010 | YLR186W | Protein required for the maturation of the 18S rRNA and for 40S ribosome production; associated with spindle/microtubules; nuclear localization depends on physical interaction with Nop14p | | | | | | | | | | | | | | | | | | | |  |  |  |
| **Afu2g13980** | **YLR276C** | **ATP-dependent RNA helicase of the DEAD-box family involved in biogenesis of the 60S ribosomal subunit** | | | | | | | | |  | | | |  |  |  |  |  |  |  |  |  |  |
| Afu6g12930 | YLR304C | Aconitase, required for the tricarboxylic acid (TCA) cycle and also independently required for mitochondrial genome maintenance; component of the mitochondrial nucleoid. | | | | | | | | | | | | | | | | | | | |  |  |  |
| **Afu2g13020** | **YLR348C** | **Mitochondrial dicarboxylate carrier, integral membrane protein, catalyzes a dicarboxylate-phosphate exchange across the inner mitochondrial membrane.** | | | | | | | | | | | | | | | | | | | | |  |  |
| Afu3g04310 | YLR409C | Possible U3 snoRNP protein involved in maturation of pre-18S rRNA, based on computational analysis of large-scale protein-protein interaction data | | | | | | | | | | | | | | | |  |  |  |  |  |  |  |
| Afu6g02060 | YLR430W | Putative helicase required for RNA polymerase II transcription termination and processing of RNAs; homolog of Senataxin which causes Ataxia-Oculomotor Apraxia 2. | | | | | | | | | | | | | | | | | | | | |  |  |
| Afu4g09140 | YLR438W | L-ornithine transaminase (OTAse), catalyzes the second step of arginine degradation, expression is dually-regulated by allophanate induction and a specific arginine induction process. | | | | | | | | | | | | | | | | | | | | | |  |
| Afu5g05450 | YML063W | Ribosomal protein 10 (rp10) of the small (40S) subunit; nearly identical to Rps1Ap and has similarity to rat S3a ribosomal protein | | | | | | | | | | | | | |  |  |  |  |  |  |  |  |  |
| Afu4g12480 | YML096W | Putative protein of unknown function with similarity to asparagine synthetases; green fluorescent protein (GFP)-fusion protein localizes to the cytoplasm;. | | | | | | | | | | | | | | | | | | | | | |  |
| Afu3g08640 | YMR146C | Subunit of the core complex of translation initiation factor 3(eIF3), which is essential for translation | | | | | | | | |  | | | |  |  |  |  |  |  |  |  |  |  |
| **Afu2g16040** | **YMR229C** | **Protein required for the synthesis of both 18S and 5.8S rRNA; C-terminal region is crucial for the formation of 18S rRNA and N-terminal region is required for the 5.8S rRNA.** | | | | | | | | | | | | | | | | | | | | | |  |
| Afu6g13490 | YMR250W | Glutamate decarboxylase, converts glutamate into gamma-aminobutyric acid (GABA) during glutamate catabolism; involved in response to oxidative stress | | | | | | | | | | | | | | | |  |  |  |  |  |  |  |
| Afu6g02520 | YMR260C | Translation initiation factor eIF1A, essential protein that forms a complex with Sui1p (eIF1) and the 40S ribosomal subunit and scans for the start codon. | | | | | | | | | | | | | | | | | | | | | |  |
| Afu2g02120 | YMR278W | Putative protein of unknown function; green fluorescent protein (GFP)-fusion protein localizes to the cytoplasm and nucleus; YMR278W is not an essential gene | | | | | | | | | | | | | | | | |  |  |  |  |  |  |
| **Afu3g05840** | **YNL175C** | **Protein of unknown function, localizes to the nucleolus and nucleoplasm; contains an RNA recognition motif (RRM) and has similarity to Nop12p.** | | | | | | | | | | | | | | | | | | |  |  |  |  |
| Afu3g06580 | YNL182C | Essential component of the Rix1 complex (Rix1p, Ipi1p, Ipi3p) that is required for processing of ITS2 sequences from 35S pre-rRNA; highly conserved and contains WD40 motifs. | | | | | | | | | | | | | | | | | | | | | | |
| **Afu8g03930** | **YNL209W** | **Cytoplasmic ATPase that is a ribosome-associated molecular chaperone, functions with J-protein partner Zuo1p; may be involved in the folding of newly-synthesized polypeptide chains.** | | | | | | | | | | | | | | | | | | | | | |  |
| **Afu3g05320** | **YNL227C** | **Co-chaperone that stimulates the ATPase activity of Ssa1p, required for a late step of ribosome biogenesis; associated with the cytosolic large ribosomal subunit.** | | | | | | | | | | | | | | | | | | | | | |  |
| Afu5g04240 | YNL251C | RNA-binding protein that interacts with the C-terminal domain of the RNA polymerase II large subunit (Rpo21p), required for transcription termination. | | | | | | | | | | | | | | | | | | |  |  |  |  |
| **Afu1g07630** | **YNL255C** | **Protein with seven cysteine-rich CCHC zinc-finger motifs, similar to human CNBP, proposed to be involved in the RAS/cAMP signaling pathway** | | | | | | | | | | | | | | |  |  |  |  |  |  |  |  |
| Afu5g12310 | YNL292W | Pseudouridine synthase, catalyzes only the formation of pseudouridine-55 (Psi55), a highly conserved tRNA modification, in mitochondrial and cytoplasmic tRNAs;. | | | | | | | | | | | | | | | | | | | | | | |
| Afu7g04130 | YNL299W | Poly (A) polymerase involved in nuclear RNA quality control based on: homology with Trf4p, genetic interactions with TRF4 mutants, physical interaction with Mtr4p (TRAMP subunit). | | | | | | | | | | | | | | | | | | | | | | |
| Afu2g05430 | YNR012W | Uridine/cytidine kinase, component of the pyrimidine ribonucleotide salvage pathway that converts uridine into UMP and cytidine into CMP. | | | | | | | | | | | | | | | | | | | | | |  |
| **Afu4g08930** | **YNR053C** | **Putative GTPase that associates with pre-60S ribosomal subunits in the nucleolus and is required for their nuclear export and maturation** | | | | | | | | | | | | | | |  |  |  |  |  |  |  |  |
| Afu1g06690 | YOR006C | Putative protein of unknown function; green fluorescent protein (GFP)-fusion protein localizes to both the cytoplasm and the nucleus | | | | | | | | | | | | | | |  |  |  |  |  |  |  |  |
| Afu1g16730 | YOR243C | Pseudouridine synthase, catalyzes pseudouridylation at position 35 in U2 snRNA, position 13 in cytoplasmic tRNAs, and position 35 in pre-tRNA(Tyr). | | | | | | | | | | | | | | | | | | | | | | |
| **Afu4g07630** | **YOR272W** | **Constituent of 66S pre-ribosomal particles, required for maturation of the large ribosomal subunit** | | | | | | | | |  | | | |  |  |  |  |  |  |  |  |  |  |
| Afu1g02030 | YOR361C | Subunit of the core complex of translation initiation factor 3(eIF3), essential for translation. | | | | | | | | | | | | | | | | | | |  |  |  |  |
| **Afu2g11810** | **YPL012W** | **Protein required for export of the ribosomal subunits; associates with the RNA components of the pre-ribosomes; contains HEAT-repeats** | | | | | | | | | | | | | | |  |  |  |  |  |  |  |  |
| Afu3g05570 | YPL054W | Zinc-finger protein of unknown function |  | |  | |  | | |  | | | |  |  |  |  |  |  |  |  |  |  |  |
| Afu7g02610 | YPL126W | U3 snoRNP protein, component of the small (ribosomal) subunit (SSU) processosome containing U3 snoRNA; required for the biogenesis of18S rRNA | | | | | | | | | | | | | | | |  |  |  |  |  |  |  |
| **Afu6g07490** | **YPL183C** | **Cytoplasmic protein of unknown function** |  | |  | |  | | |  | | | |  |  |  |  |  |  |  |  |  |  |  |
| Afu6g11510 | YPL207W | Protein required for the synthesis of wybutosine, a modified guanosine found at the 3'-position adjacent to the anticodon of phenylalanine tRNA. | | | | | | | | | | | | | | | | | | | | | |  |
| Afu2g08780 | YPR095C | Guanine nucleotide exchange factor (GEF) for Arf proteins; involved in vesicular transport; suppressor of ypt3 mutations; member of the Sec7-domain family | | | | | | | | | | | | | | | |  |  |  |  |  |  |  |
| Afu4g07500 | YPR137W | Protein involved in pre-rRNA processing, associated with U3 snRNP; component of small ribosomal subunit (SSU) processosome; ortholog of the human U3-55k protein | | | | | | | | | | | | | | | | |  |  |  |  |  |  |
| Afu8g05430 | YPR144C | Nucleolar protein, forms a complex with Nop14p that mediates maturation and nuclear export of 40S ribosomal subunits | | | | | | | | | | | | | |  |  |  |  |  |  |  |  |  |
| Afu3g06540 | YPR167C | 3'-phosphoadenylsulfate reductase, reduces 3'-phosphoadenylyl sulfate to adenosine-3',5'-bisphosphate and free sulfite using reduced thioredoxin as cosubstrate. | | | | | | | | | | | | | | | | | | | | |  |  |

Orthologous proteins in the genomes were identified using a reciprocal-best-BLAST-hit (RBH) approach with a cut-off of 1e-05. Genes represented among both host-adaptation and nitrogen starvation datasets are indicated in bold underlined text.
