# Supplementary material for: Sub-Telomere Directed Gene Expression during Initiation of Invasive Aspergillosis
Source: PLoS Pathog. 2008 Sep 12;4(9):e1000154. doi: 10.1371/journal.ppat.1000154 (PMC2526178; doi:10.1371/journal.ppat.1000154)
Supplement: Table S5 — Asterisked gene lists (0.34 MB DOC) [file ppat.1000154.s008.doc]

**Table S5**

***A. fumigatus*- and Affc-specific genes having differential transcript abundance in the murine lung, relative to laboratory culture**

The Affc-core set were defined as *A. fumigatus* Af293 proteins that have orthologs in *N. fischeri* and *A. clavatus*. The Affc-unique set is a sub-set of Affc-core proteins that do not have orthologs in *A. terreus*, *A. oryzae*, *A. nidulans* or *A. niger.*

**Af-specific genes having increased transcript abundance**

| **Accession** | **Old accession** | **Protein name** | **Location** | **SignalP** | **Process** |
| --- | --- | --- | --- | --- | --- |
| AFUA_1G00100 | Afu1g00100 | MFS monocarboxylate transporter, putative | subtelomeric | SignalP | transport |
| AFUA_1G00450 | Afu1g00450 | N-acetylglucosamine-6-phosphate deacetylase (NagA), putative | subtelomeric |  | metabolism |
| AFUA_1G01580 | Afu1g01580 | hypothetical protein |  |  | unknown |
| AFUA_1G05400 | Afu1g05400 | hypothetical protein |  |  | unknown |
| AFUA_1G11030 | Afu1g11030 | xylitol dehydrogenase |  |  | metabolism |
| AFUA_1G11240 | Afu1g11240 | hypothetical protein |  |  | unknown |
| AFUA_1G15190 | Afu1g15190 | hypothetical protein |  |  | unknown |
| AFUA_2G00490 | Afu2g00490 | glycosyl hydrolase, family 31 | subtelomeric |  | metabolism |
| AFUA_2G00550 | Afu2g00550 | conserved hypothetical protein | subtelomeric |  | unknown |
| AFUA_2G00700 | Afu2g00700 | hypothetical protein | subtelomeric |  | unknown |
| AFUA_2G02330 | Afu2g02330 | hypothetical protein |  |  | unknown |
| AFUA_2G04530 | Afu2g04530 | hypothetical protein |  |  | unknown |
| AFUA_2G05300 | Afu2g05300 | hypothetical protein |  |  | unknown |
| AFUA_2G11530 | Afu2g11530 | hypothetical protein |  |  | unknown |
| AFUA_2G12740 | Afu2g12740 | methyltransferase, putative |  |  | metabolism |
| AFUA_2G17640 | Afu2g17640 | conserved hypothetical protein | subtelomeric |  | unknown |
| AFUA_2G17910 | Afu2g17910 | conserved hypothetical protein | subtelomeric | SignalP | unknown |
| AFUA_2G18050 | Afu2g18050 | FAD binding oxidoreductase CpoX1 | subtelomeric | SignalP | metabolism |
| AFUA_2G18060 | Afu2g18060 | AdoMet:dimethylallyltryptophan N-methyltransferase EasF | subtelomeric |  | metabolism |
| AFUA_3G01060 | Afu3g01060 | hypothetical protein | subtelomeric |  | unknown |
| AFUA_3G01140 | Afu3g01140 | hypothetical protein | subtelomeric |  | unknown |
| AFUA_3G01230 | Afu3g01230 | MFS sugar transporte, putative |  |  | transport |
| AFUA_3G01730 | Afu3g01730 | hypothetical protein |  |  | unknown |
| AFUA_3G02110 | Afu3g02110 | MFS multidrug transporter, putative |  | SignalP | transport |
| AFUA_3G02130 | Afu3g02130 | oxidoreductase, zinc-binding, putative |  |  | metabolism |
| AFUA_3G02420 | Afu3g02420 | ThiJ/PfpI family transcriptional regulator, putative |  |  | transcriptional regulation |
| AFUA_3G03600 | Afu3g03600 | carboxylesterase, putative |  |  | metabolism |
| AFUA_3G03750 | Afu3g03750 | hypothetical protein |  |  | unknown |
| AFUA_3G07090 | Afu3g07090 | hypothetical protein |  |  | unknown |
| AFUA_3G11880 | Afu3g11880 | hypothetical protein |  |  | unknown |
| AFUA_3G14740 | Afu3g14740 | conserved hypothetical protein | subtelomeric |  | unknown |
| AFUA_4G00860 | Afu4g00860 | cell surface protein, putative | subtelomeric |  | cell wall biogenesis |
| AFUA_4G06580 | Afu4g06580 | hypothetical protein |  |  | unknown |
| AFUA_4G06660 | Afu4g06660 | hypothetical protein |  |  | unknown |
| AFUA_4G08840 | Afu4g08840 | RING finger protein, putative |  | SignalP | unknown |
| AFUA_4G13520 | Afu4g13520 | oxidoreductase, short-chain dehydrogenase/reductase family |  |  | metabolism |
| AFUA_5G00190 | Afu5g00190 | hypothetical protein | subtelomeric |  | unknown |
| AFUA_5G00390 | Afu5g00390 | hypothetical protein | subtelomeric |  | unknown |
| AFUA_5G00490 | Afu5g00490 | hypothetical protein | subtelomeric |  | unknown |
| AFUA_5G01410 | Afu5g01410 | hypothetical protein |  |  | unknown |
| AFUA_5G01610 | Afu5g01610 | hypothetical protein |  |  | unknown |
| AFUA_5G02960 | Afu5g02960 | hypothetical protein |  |  | unknown |
| AFUA_5G10350 | Afu5g10350 | conserved hypothetical protein |  | SignalP | unknown |
| AFUA_5G12220 | Afu5g12220 | conserved hypothetical protein |  |  | unknown |
| AFUA_5G13770 | Afu5g13770 | hypothetical protein |  |  | unknown |
| AFUA_5G14280 | Afu5g14280 | integral membrane protein | subtelomeric |  | unknown |
| AFUA_5G14900 | Afu5g14900 | hypothetical protein | subtelomeric |  | unknown |
| AFUA_6G00140 | Afu6g00140 | hypothetical protein | subtelomeric | SignalP | unknown |
| AFUA_6G00690 | Afu6g00690 | conserved hypothetical protein | subtelomeric | SignalP | unknown |
| AFUA_6G02290 | Afu6g02290 | hypothetical protein |  |  | unknown |
| AFUA_6G03220 | Afu6g03220 | hypothetical protein |  |  | unknown |
| AFUA_6G07790 | Afu6g07790 | hypothetical protein |  |  | unknown |
| AFUA_6G10180 | Afu6g10180 | spherulin 4 family protein, putative |  |  | unknown |
| AFUA_6G10190 | Afu6g10190 | hypothetical protein |  |  | unknown |
| AFUA_6G11500 | Afu6g11500 | dipeptidase, putative |  |  | metabolism |
| AFUA_6G11950 | Afu6g11950 | hypothetical protein |  |  | unknown |
| AFUA_6G11990 | Afu6g11990 | hypothetical protein |  |  | unknown |
| AFUA_6G12100 | Afu6g12100 | nitrilase family protein |  |  | metabolism |
| AFUA_7G00790 | Afu7g00790 | hypothetical protein | subtelomeric |  | unknown |
| AFUA_7G01020 | Afu7g01020 | hypothetical protein | subtelomeric |  | unknown |
| AFUA_7G04560 | Afu7g04560 | hypothetical protein |  |  | unknown |
| AFUA_7G07130 | Afu7g07130 | hypothetical protein |  |  | unknown |
| AFUA_7G08450 | Afu7g08450 | ornithine decarboxylase, putative | subtelomeric |  | metabolism |
| AFUA_8G00100 | Afu8g00100 | aspartate-tRNA ligase, putative | subtelomeric |  | metabolism |
| AFUA_8G00490 | Afu8g00490 | PKS-like enzyme, putative | subtelomeric |  | metabolism |
| AFUA_8G00500 | Afu8g00500 | acetate-CoA ligase, putative | subtelomeric |  | metabolism |
| AFUA_8G00810 | Afu8g00810 | hypothetical protein | subtelomeric |  | unknown |
| AFUA_8G01460 | Afu8g01460 | hypothetical protein |  |  | unknown |
| AFUA_8G01650 | Afu8g01650 | hypothetical protein |  |  | unknown |
| AFUA_8G01950 | Afu8g01950 | hypothetical protein |  | SignalP | unknown |
| AFUA_8G02260 | Afu8g02260 | neutral amino acid permease |  |  | transport |
| AFUA_8G05050 | Afu8g05050 | hypothetical protein |  |  | unknown |
| AFUA_8G05080 | Afu8g05080 | hypothetical protein |  |  | unknown |
| AFUA_8G05470 | Afu8g05470 | hypothetical protein |  |  | unknown |
| AFUA_8G05770 | Afu8g05770 | hypothetical protein |  |  | unknown |
| AFUA_8G05780 | Afu8g05780 | NACHT and Ankyrin domain protein |  |  | signaling |
| AFUA_8G06640 | Afu8g06640 | ubiE/COQ5 methyltransferase, putative | subtelomeric |  | metabolism |

**Af-specific genes having decreased transcript abundance**

| **Accession** | **Old accession** | **Protein name** | **Location** | **SignalP** | **Process** |
| --- | --- | --- | --- | --- | --- |
| AFUA_1G00270 | Afu1g00270 | hypothetical protein | subtelomeric | | unknown |
| AFUA_1G00310 | Afu1g00310 | class V chitinase, putative | subtelomeric | SignalP | cell wall biogenesis |
| AFUA_1G01010 | Afu1g01010 | polyketide synthase, putative |  |  | metabolism |
| AFUA_1G02220 | Afu1g02220 | hypothetical protein |  |  | unknown |
| AFUA_1G02960 | Afu1g02960 | conserved hypothetical protein |  |  | unknown |
| AFUA_1G03230 | Afu1g03230 | ABC multidrug transporter, putative |  |  | transport |
| AFUA_1G03270 | Afu1g03270 | hypothetical protein |  |  | unknown |
| AFUA_1G17420 | Afu1g17420 | hypothetical protein | subtelomeric | | unknown |
| AFUA_2G01470 | Afu2g01470 | hypothetical protein |  |  | unknown |
| AFUA_2G05280 | Afu2g05280 | F-box domain protein |  |  | unknown |
| AFUA_2G06160 | Afu2g06160 | alpha-1,3-glucanase/mutanase, putative |  |  | cell wall biogenesis |
| AFUA_2G09460 | Afu2g09460 | potassium transporter |  |  | transport |
| AFUA_2G16440 | Afu2g16440 | hypothetical protein |  |  | unknown |
| AFUA_3G02180 | Afu3g02180 | NRPS-like enzyme, putative |  |  | metabolism |
| AFUA_3G07010 | Afu3g07010 | hypothetical protein |  |  | unknown |
| AFUA_3G09120 | Afu3g09120 | hypothetical protein |  |  | unknown |
| AFUA_3G09510 | Afu3g09510 | 3-oxoacyl-(acyl-carrier-protein) reductase |  |  | metabolism |
| AFUA_3G09660 | Afu3g09660 | hypothetical protein |  |  | unknown |
| AFUA_3G13710 | Afu3g13710 | GTP cyclohydrolase I, putative |  |  | metabolism |
| AFUA_3G13720 | Afu3g13720 | oxidoreductase, 2OG-Fe(II) oxygenase family, putative |  |  | metabolism |
| AFUA_4G00510 | Afu4g00510 | hypothetical protein | subtelomeric | | unknown |
| AFUA_4G00850 | Afu4g00850 | hypothetical protein | subtelomeric | | unknown |
| AFUA_4G00880 | Afu4g00880 | hypothetical protein | subtelomeric | | unknown |
| AFUA_4G01430 | Afu4g01430 | hypothetical protein |  | SignalP | unknown |
| AFUA_4G06810 | Afu4g06810 | hypothetical protein |  |  | unknown |
| AFUA_4G07920 | Afu4g07920 | hypothetical protein |  |  | unknown |
| AFUA_4G08860 | Afu4g08860 | hypothetical protein |  |  | unknown |
| AFUA_5G00170 | Afu5g00170 | extracellular serine threonine rich protein, putative | subtelomeric | SignalP | cell wall biogenesis |
| AFUA_5G00220 | Afu5g00220 | hypothetical protein | subtelomeric | | unknown |
| AFUA_5G00230 | Afu5g00230 | hypothetical protein | subtelomeric | | unknown |
| AFUA_5G00860 | Afu5g00860 | Ankyrin repeat protein | subtelomeric | | unknown |
| AFUA_5G01070 | Afu5g01070 | C6 transcription factor, putative | subtelomeric | | transcriptional regulation |
| AFUA_5G02710 | Afu5g02710 | hypothetical protein |  |  | unknown |
| AFUA_5G13870 | Afu5g13870 | mRNA export factor Mlo3, putative |  |  | metabolism |
| AFUA_5G15140 | Afu5g15140 | hypothetical protein | subtelomeric | | unknown |
| AFUA_6G00180 | Afu6g00180 | hypothetical protein | subtelomeric | | unknown |
| AFUA_6G00350 | Afu6g00350 | hypothetical protein | subtelomeric | | unknown |
| AFUA_6G03120 | Afu6g03120 | hypothetical protein |  |  | unknown |
| AFUA_6G09310 | Afu6g09310 | class V chitinase, putative |  | SignalP | cell wall biogenesis |
| AFUA_6G09340 | Afu6g09340 | hypothetical protein |  |  | unknown |
| AFUA_6G10150 | Afu6g10150 | hypothetical protein |  |  | unknown |
| AFUA_6G10350 | Afu6g10350 | hypothetical protein |  |  | unknown |
| AFUA_6G11630 | Afu6g11630 | FAD-dependent isoamyl alcohol oxidase, putative |  | SignalP | metabolism |
| AFUA_6G11700 | Afu6g11700 | hypothetical protein |  |  | unknown |
| AFUA_7G00400 | Afu7g00400 | hypothetical protein | subtelomeric | | unknown |
| AFUA_7G04750 | Afu7g04750 | hypothetical protein |  |  | unknown |
| AFUA_7G05710 | Afu7g05710 | hypothetical protein |  |  | unknown |
| AFUA_7G06000 | Afu7g06000 | hypothetical protein |  |  | unknown |
| AFUA_7G07040 | Afu7g07040 | hypothetical protein |  |  | unknown |
| AFUA_7G07060 | Afu7g07060 | hypothetical protein |  |  | unknown |
| AFUA_7G07140 | Afu7g07140 | hypothetical protein | subtelomeric | | unknown |
| AFUA_7G08250 | Afu7g08250 | C6 finger domain protein, putative | subtelomeric | | transcriptional regulation |
| AFUA_7G08380 | Afu7g08380 | hypothetical protein | subtelomeric | | unknown |
| AFUA_7G08470 | Afu7g08470 | peroxisomal copper amine oxidase, putative | subtelomeric | | metabolism |
| AFUA_7G08520 | Afu7g08520 | hypothetical protein | subtelomeric | | unknown |
| AFUA_8G02110 | Afu8g02110 | hypothetical protein |  |  | unknown |
| AFUA_8G06150 | Afu8g06150 | serine-threonine rich protein, putative |  |  | unknown |
| AFUA_8G06370 | Afu8g06370 | monooxygenase, putative |  |  | metabolism |

**Affc-specific genes having increased transcript abundance**

| **Accession** | **Old Accession** | **Protein name** | **Location** | **signalP** | **Process** |
| --- | --- | --- | --- | --- | --- |
| AFUA_1G04160 | Afu1g04160 | aspartate aminotransferase, putative |  |  | metabolism |
| AFUA_1G05910 | Afu1g05910 | conserved hypothetical protein |  |  | unknown |
| AFUA_1G11370 | Afu1g11370 | GMC oxidoreductase, putative |  | signalP | metabolism |
| AFUA_1G13410 | Afu1g13410 | conserved hypothetical protein |  |  | unknown |
| AFUA_1G16740 | Afu1g16740 | conserved hypothetical protein |  |  | unknown |
| AFUA_1G17370 | Afu1g17370 | HSP9/12 family heat shock protein | subtelomeric |  | unknown |
| AFUA_1G17600 | Afu1g17600 | conserved hypothetical protein | subtelomeric |  | unknown |
| AFUA_2G00580 | Afu2g00580 | conserved hypothetical protein | subtelomeric |  | unknown |
| AFUA_2G00990 | Afu2g00990 | glycerophosphoryl diester phosphodiesterase family protein | subtelomeric |  | metabolism |
| AFUA_2G02500 | Afu2g02500 | conserved hypothetical protein |  |  | unknown |
| AFUA_2G12780 | Afu2g12780 | von Willebrand domain protein |  |  |  |
| AFUA_2G12850 | Afu2g12850 | 1,3-beta-glucanosyltransferase Gel3 |  | signalP | cell wall biogenesis |
| AFUA_2G17420 | Afu2g17420 | Pfs and NB-ARC domain protein | subtelomeric |  | signaling |
| AFUA_2G17650 | Afu2g17650 | DUF907 domain protein | subtelomeric | signalP | unknown |
| AFUA_3G01130 | Afu3g01130 | cell wall protein, putative | subtelomeric | signalP | cell wall biogenesis |
| AFUA_3G01320 | Afu3g01320 | homocysteine S-methyltransferase, putative |  |  | metabolism |
| AFUA_3G02800 | Afu3g02800 | lipase/esterase, putative |  |  | metabolism |
| AFUA_3G12220 | Afu3g12220 | ABC transporter, putative |  |  | transport |
| AFUA_3G12610 | Afu3g12610 | conserved hypothetical protein |  |  | unknown |
| AFUA_3G13130 | Afu3g13130 | HHE domain protein |  |  | metabolism |
| AFUA_3G13290 | Afu3g13290 | conserved hypothetical protein |  |  | unknown |
| AFUA_3G14770 | Afu3g14770 | NAD dependent epimerase/dehydratase family protein | subtelomeric | signalP | metabolism |
| AFUA_3G15080 | Afu3g15080 | conserved hypothetical protein | subtelomeric |  | unknown |
| AFUA_4G01360 | Afu4g01360 | MFS transporter of unkown specificity |  |  | transport |
| AFUA_4G01370 | Afu4g01370 | pyoverdine/dityrosine biosynthesis family protein |  |  | signaling |
| AFUA_4G01392 | Afu4g01390 | C6 transcription factor, putative |  |  | transcriptional regulation |
| AFUA_4G01470 | Afu4g01470 | C6 finger domain protein, putative |  |  | transcriptional regulation |
| AFUA_4G08390 | Afu4g08390 | conserved hypothetical protein |  |  | unknown |
| AFUA_4G13950 | Afu4g13950 | GNAT family acetyltransferase, putative | subtelomeric |  | metabolism |
| AFUA_4G14170 | Afu4g14170 | conserved hypothetical protein | subtelomeric | signalP | unknown |
| AFUA_4G14180 | Afu4g14180 | conserved hypothetical protein | subtelomeric | signalP | unknown |
| AFUA_4G14200 | Afu4g14200 | conserved hypothetical protein | subtelomeric | signalP | unknown |
| AFUA_5G00810 | Afu5g00810 | conserved hypothetical protein | subtelomeric |  | unknown |
| AFUA_5G01130 | Afu5g01130 | conserved hypothetical protein | subtelomeric |  | unknown |
| AFUA_5G01300 | Afu5g01300 | integral membrane protein |  | signalP | unknown |
| AFUA_5G08180 | Afu5g08180 | cell wall protein, putative |  | signalP | cell wall biogenesis |
| AFUA_5G09130 | Afu5g09130 | polysaccharide deacetylase family protein |  |  | metabolism |
| AFUA_5G09440 | Afu5g09440 | amino acid permease, putative |  |  | transport |
| AFUA_6G00620 | Afu6g00620 | GPI anchored hypothetical protein | subtelomeric |  | cell wall biogenesis |
| AFUA_6G03550 | Afu6g03550 | conserved hypothetical protein |  |  | unknown |
| AFUA_6G04280 | Afu6g04280 | integral membrane protein |  | signalP | unknown |
| AFUA_6G07260 | Afu6g07260 | purine-cytosine permease, putative |  |  | transport |
| AFUA_6G07920 | Afu6g07920 | acetyltransferase, GNAT family family |  |  | metabolism |
| AFUA_6G08500 | Afu6g08500 | phosphoglycerate mutase family protein |  |  | metabolism |
| AFUA_6G08650 | Afu6g08650 | conserved hypothetical protein |  |  | unknown |
| AFUA_6G12150 | Afu6g12150 | bZIP transcription factor, putative |  |  | transcriptional regulation |
| AFUA_7G00690 | Afu7g00690 | aminotransferase, putative | subtelomeric |  | metabolism |
| AFUA_7G01000 | Afu7g01000 | aldehyde dehydrogenase, putative | subtelomeric |  | metabolism |
| AFUA_7G04990 | Afu7g04990 | dUTP diphosphatase, putative |  |  | uracil metabolism |
| AFUA_7G05650 | Afu7g05650 | XYPPX repeat family protein |  |  | unknown |
| AFUA_8G00580 | Afu8g00580 | glutathione S-transferase, putative | subtelomeric |  | metabolism |
| AFUA_8G00710 | Afu8g00710 | secreted antimicrobial peptide, putative | subtelomeric | signalP | unknown |
| AFUA_8G01770 | Afu8g01770 | conserved hypothetical protein |  | signalP | unknown |
| AFUA_8G05930 | Afu8g05930 | conserved hypothetical protein |  |  | unknown |

**Affc-specific genes having decreased transcript abundance**

| **Accession** | **Old Accession** | **Protein name** | **Location** | **SignalP** | **Process** |
| --- | --- | --- | --- | --- | --- |
| AFUA_1G12220 | Afu1g12220 | conserved hypothetical protein |  |  | unknown |
| AFUA_1G13120 | Afu1g13120 | MIT/CorA family metal ion transporter |  |  | unknown |
| AFUA_2G02650 | Afu2g02650 | conserved hypothetical protein |  |  | unknown |
| AFUA_3G09260 | Afu3g09260 | conserved hypothetical protein |  | SignalP | unknown |
| AFUA_3G12680 | Afu3g12680 | conserved hypothetical protein |  |  | unknown |
| AFUA_4G08890 | Afu4g08890 | aldo-keto reductase family protein |  |  | metabolism |
| AFUA_5G10940 | Afu5g10940 | conserved hypothetical protein |  |  | unknown |
| AFUA_6G00500 | Afu6g00500 | chitosanase, putative | subtelomeric | SignalP | cell wall biogenesis |
| AFUA_7G06440 | Afu7g06440 | F-box domain protein |  |  | unknown |
| AFUA_8G01820 | Afu8g01820 | hypothetical protein |  |  | unknown |
