# Supplementary material for: Sub-Telomere Directed Gene Expression during Initiation of Invasive Aspergillosis
Source: PLoS Pathog. 2008 Sep 12;4(9):e1000154. doi: 10.1371/journal.ppat.1000154 (PMC2526178; doi:10.1371/journal.ppat.1000154)
Supplement: Table S6 — Cluster overview (1.33 MB DOC) [file ppat.1000154.s009.doc]

**Table S6**

**Identification and phylogenetic conservation of physically linked, co-ordinately differentially expressed *A. fumigatus* gene clusters from transcriptome analyses.** Conserved proteins are indicated as identities (%) with respect to *N. fischeri* (Nf), *A. clavatus* (Ac), *A. nidulans* (An) and *A. oryzae* (Ao). A full version of this table indicating between-species synteny within cluster loci, and all accession numbers, can be viewed in Supplementary Data 2.

| **cluster** | **Accession** | **New Accessions** | **Protein Names** | **NF** | **Ac** | **An** | **Ao** | **Expression ratio** |
| --- | --- | --- | --- | --- | --- | --- | --- | --- |
| cluster 1 | Afu1g01180 | AFUA_1G01180 | isoamyl alcohol oxidase, putative | 95 | 77 | 63 | 75 | 3.131991271 |
|  | Afu1g01200 | AFUA_1G01200 | alpha-galactosidase | 94 |  | 60 |  | 6.513162955 |
|  | Afu1g01210 | AFUA_1G01210 | hypothetical protein | 90 |  |  |  | 2.143656428 |
|  | Afu1g01220 | AFUA_1G01220 | conserved hypothetical protein | 96 |  | 68 | 76 | 4.58415831 |
|  | Afu1g01240 | AFUA_1G01240 | C6 transcription factor, putative | 94 |  |  | 58 | 2.892836902 |
|  | Afu1g01260 | AFUA_1G01260 | MFS transporter, putative | 97 |  |  | 66 | 3.828839557 |
| cluster 2 | Afu1g01530 | AFUA_1G01530 | GrpB domain protein | 93 | 68 | 61 | 65 | 4.22532319 |
|  | Afu1g01540 | AFUA_1G01540 | endonuclease/exonuclease/phosphatase family protein | 93 | 77 | 58 | 72 | 5.101467805 |
|  | Afu1g01550 | AFUA_1G01550 | high affinity zinc ion transporter, putative | 94 |  | 62 | 73 | 4.129116869 |
|  | Afu1g01580 | AFUA_1G01580 | hypothetical protein |  |  |  |  | 2.28441838 |
|  | Afu1g01600 | AFUA_1G01600 | deoxyribodipyrimidine photo-lyase Phr1, putative | 93 | 78 | 71 | 72 | 2.805471906 |
|  | Afu1g01610 | AFUA_1G01610 | conserved hypothetical protein | 84 | 65 | 59 | 51 | 3.04063218 |
| cluster 3 | Afu1g01680 | AFUA_1G01680 | branched-chain amino acid aminotransferase | 96 | 84 | 76 | 78 | 2.875467059 |
|  | Afu1g01690 | AFUA_1G01690 | cytochrome P450 alkane hydroxylase, putative | 95 | 77 | 64 | 78 | 3.181036625 |
|  | Afu1g01700 | AFUA_1G01700 | conserved serine-rich protein | 89 | 51 | 35 | 57 | 2.059137268 |
|  | Afu1g01720 | AFUA_1G01720 | protein kinase and ribonuclease Ire1, putative | 96 | 87 | 76 | 83 | 2.048819718 |
|  | Afu1g01770 | AFUA_1G01770 | short chain oxidoreductase/dehydrogenase, putative | 96 | 82 | 53 | 54 | 2.727846935 |
| cluster 4 | Afu1g03280 | AFUA_1G03280 | MFS alpha-glucoside transporter, putative | 96 |  |  |  | 2.608455529 |
|  | Afu1g03300 | AFUA_1G03300 | carboxylesterase, putative | 92 |  |  |  | 3.790045526 |
|  | Afu1g03350 | AFUA_1G03352 | alpha-1,3-glucanase/mutanase, putative | 88 |  |  | 78 | 2.058913677 |
|  | Afu1g03360 | AFUA_1G03360 | conserved hypothetical protein | 80 |  |  | 35 | 2.123715853 |
|  | Afu1g03380 | AFUA_1G03380 | UDP-N-acetylglucosaminyltransferase | 95 | 81 | 70 | 72 | 2.427899215 |
| cluster 5 | Afu1g03780 | AFUA_1G03780 | C6 finger domain protein, putative | 97 | 77 |  | 65 | -2.009326086 |
|  | Afu1g03790 | AFUA_1G03790 | mannosylphosphorylation protein (Mnn4), putative | 94 | 80 | 69 |  | -2.121111996 |
|  | Afu1g03840 | AFUA_1G03840 | CCAAT-binding factor complex subunit HapC | 94 | 87 | 76 | 82 | -2.071861565 |
|  | Afu1g03910 | AFUA_1G03910 | Spo7-like protein | 97 | 79 | 64 | 79 | -3.446396208 |
|  | Afu1g03920 | AFUA_1G03920 | DNA replication licensing factor Mcm3, putative | 99 | 93 | 84 | 86 | -2.468522375 |
|  | Afu1g03930 | AFUA_1G03930 | translation regulator (Cya5), putative | 95 | 68 | 42 | 61 | -2.073391018 |
|  | Afu1g03950 | AFUA_1G03950 | cytochrome P450 sterol C-22 desaturase, putaitve | 99 | 88 | 79 | 82 | -2.135790025 |
|  | Afu1g03990 | AFUA_1G03992 | conserved hypothetical protein | 92 | 78 | 63 | 69 | -2.801081699 |
| cluster 6 | Afu1g04070 | AFUA_1G04070 | Eukaryotic translation initiation factor eIF-5A | 98 | 88 | 88 | 83 | -2.325995856 |
|  | Afu1g05910 | AFUA_1G05910 | conserved hypothetical protein | 83 | 51 |  |  | 2.468115877 |
|  | Afu1g05940 | AFUA_1G05940 | RNA polymerase II mediator complex protein Nut2, putative | 93 | 82 | 71 |  | 2.183114466 |
|  | Afu1g05960 | AFUA_1G05960 | Peptidase family M28 family | 94 | 76 | 69 | 68 | 2.328159347 |
|  | Afu1g05970 | AFUA_1G05970 | ubiquitin ligase complex F-box protein GRR1, putative | 98 | 89 | 78 | 83 | 2.570559503 |
|  | Afu1g05980 | AFUA_1G05980 | AMP-binding domain protein, putative | 98 | 88 | 82 | 84 | 3.405374839 |
| cluster 7 | Afu1g07140 | AFUA_1G07140 | c-24(28) sterol reductase | 94 | 87 | 79 | 88 | -2.895486437 |
|  | Afu1g07160 | AFUA_1G07160 | ubiquitin C-terminal hydrolase, putative | 94 | 78 | 68 | 70 | -3.85275321 |
|  | Afu1g07170 | AFUA_1G07170 | Na/H antiporter, putative | 98 | 89 | 75 | 94 | -2.58652774 |
|  | Afu1g07210 | AFUA_1G07210 | conserved hypothetical protein | 99 | 93 | 90 | 93 | -3.517525517 |
|  | Afu1g07260 | AFUA_1G07260 | NUDIX family hydrolase, putative | 89 | 68 | 62 | 57 | -3.356670798 |
|  | Afu1g07280 | AFUA_1G07280 | conserved hypothetical protein | 92 | 72 | 55 | 65 | -2.323799671 |
| cluster 8 | Afu1g11010 | AFUA_1G11010 | short chain oxidoreductase/dehydrogenase, putative |  |  |  | 80 | 4.828259956 |
|  | Afu1g11020 | AFUA_1G11020 | L-arabinitol 4-dehydrogenase |  |  | 80 | 81 | 6.453130024 |
|  | Afu1g11030 | AFUA_1G11030 | xylitol dehydrogenase |  |  |  |  | 2.344713325 |
|  | Afu1g11040 | AFUA_1G11040 | conserved hypothetical protein |  |  | 49 |  | 7.033077414 |
|  | Afu1g11050 | AFUA_1G11050 | MFS sugar transporter, putative | 77 |  | 71 | 74 | 4.469808544 |
|  | Afu1g11080 | AFUA_1G11080 | serine/threonine protein kinase Kin1, putative | 98 | 88 | 79 | 79 | 2.200524463 |
| cluster 9 | Afu1g11220 | AFUA_1G11220 | GPI anchored protein, putative | 89 |  |  |  | 2.142318273 |
|  | Afu1g11240 | AFUA_1G11240 | hypothetical protein |  |  |  |  | 2.865361865 |
|  | Afu1g11280 | AFUA_1G11280 | oxidoreductase, putative | 97 | 87 |  | 44 | 3.471872803 |
|  | Afu1g11310 | AFUA_1G11310 | AMP-binding enzyme domain protein | 96 | 88 |  | 69 | 2.17047095 |
|  | Afu1g11320 | AFUA_1G11320 | class II DAHP synthetase family protein | 95 | 83 |  | 58 | 5.052366552 |
|  | Afu1g11330 | AFUA_1G11330 | lactonohydrolase, putative | 95 | 86 |  | 66 | 2.294487941 |
|  | Afu1g11370 | AFUA_1G11370 | GMC oxidoreductase, putative | 96 | 83 |  |  | 4.87173135 |
| cluster 10 | Afu1g12170 | AFUA_1G12170 | translation elongation factor EF-Tu, putative | 99 | 94 | 86 | 88 | -2.034099916 |
|  | Afu1g12220 | AFUA_1G12220 | conserved hypothetical protein | 98 | 78 |  |  | -2.283887882 |
|  | Afu1g12250 | AFUA_1G12250 | mitochondrial hypoxia responsive domain protein | 90 | 81 | 61 | 69 | -2.705832309 |
|  | Afu1g12260 | AFUA_1G12260 | transcriptional elongation factor Iws1, putative | 96 | 85 | 82 | 74 | -2.022547063 |
|  | Afu1g12280 | AFUA_1G12280 | nucleoporin, putative | 99 | 90 | 84 | 60 | -3.69374441 |
|  | Afu1g12330 | AFUA_1G12332 | jumonji family transcription factor, putative | 96 | 89 | 78 | 81 | -3.084853079 |
| cluster 11 | Afu1g14460 | AFUA_1G14460 | fungal specific transcription factor, putative | 89 |  |  | 46 | 6.682205385 |
|  | Afu1g14490 | AFUA_1G14490 | aminotransferase, putative | 94 | 75 | 64 | 68 | 2.253782725 |
|  | Afu1g14510 | AFUA_1G14510 | ankyrin repeat protein | 96 | 82 | 65 | 74 | 2.404565899 |
|  | Afu1g14520 | AFUA_1G14520 | pyridine nucleotide-disulphide oxidoreductase, putative | 95 | 74 | 67 | 73 | 3.960132532 |
|  | Afu1g14530 | AFUA_1G14530 | general amidase, putative | 94 | 73 |  | 74 | 3.57747094 |
|  | Afu1g14540 | AFUA_1G14540 | oxidoreductase, short-chain dehydrogenase/reductase family | 95 | 90 | 74 |  | 2.644464354 |
|  | Afu1g14550 | AFUA_1G14550 | Mn superoxide dismutase MnSOD | 98 | 69 | 68 |  | 4.270438573 |
| cluster 12 | Afu1g16670 | AFUA_1G16670 | conserved hypothetical protein | 87 |  |  |  | 2.013129454 |
|  | Afu1g16700 | AFUA_1G16700 | beta galactosidase, putative | 97 |  | 74 | 52 | 3.25484732 |
|  | Afu1g16740 | AFUA_1G16740 | conserved hypothetical protein | 92 | 32 |  |  | 2.631221179 |
|  | Afu1g16760 | AFUA_1G16760 | acetyltransferase, GNAT family family | 95 | 54 |  |  | 3.948924898 |
|  | Afu1g16770 | AFUA_1G16770 | amidase family protein, putative | 95 | 73 |  | 59 | 3.040674175 |
| cluster 13 | Afu1g17150 | AFUA_1G17150 | C6 transcription factor, putative | 93 | 55 | 56 | 58 | 2.205177391 |
|  | Afu1g17160 | AFUA_1G17160 | MFS transporter, putative | 92 |  | 75 |  | 3.285935426 |
|  | Afu1g17170 | AFUA_1G17170 | TfdA family taurine dioxygenase, putative | 96 | 53 | 79 | 84 | 5.108258594 |
|  | Afu1g17180 | AFUA_1G17180 | pyridine nucleotide-disulphide oxidoreductase AMID-like, putative | 96 | 72 | 46 | 63 | 6.924266405 |
|  | Afu1g17190 | AFUA_1G17190 | long-chain-fatty-acid-CoA ligase, putative | 97 | 89 | 82 | 82 | 5.657990042 |
|  | Afu1g17200 | AFUA_1G17200 | nonribosomal siderophore peptide synthase SidC | 93 |  | 55 | 63 | 2.664456516 |
| cluster 14 | Afu1g17260 | AFUA_1G17260 | hypothetical protein | 89 | 36 |  |  | 6.409797071 |
|  | Afu1g17270 | AFUA_1G17270 | FRE family ferric-chelate reductase, putative | 92 | 66 | 50 |  | 5.292143128 |
|  | Afu1g17280 | AFUA_1G17280 | conserved hypothetical protein | 97 | 76 |  | 43 | 3.538514901 |
|  | Afu1g17310 | AFUA_1G17310 | MFS lactose permease, putative | 95 | 85 | 72 |  | 2.082602787 |
|  | Afu1g17320 | AFUA_1G17320 | endo-arabinanase, putative | 94 | 65 | 54 |  | 2.780007346 |
|  | Afu1g17340 | AFUA_1G17340 | dioxygenase, putative | 80 |  |  |  | 2.532752908 |
|  | Afu1g17370 | AFUA_1G17370 | heat shock protein Awh11, putative | 93 | 72 |  |  | 2.195459835 |
| cluster 15 | Afu1g17470 | AFUA_1G17470 | high affinity nitrate transporter NrtB | 94 |  | 70 |  | 7.059699432 |
|  | Afu1g17480 | AFUA_1G17480 | MFS transporter, putative | 95 | 85 | 78 | 79 | 5.541309745 |
|  | Afu1g17490 | AFUA_1G17490 | glucokinase regulator family protein, putative | 91 | 73 | 72 | 37 | 2.787653728 |
|  | Afu1g17520 | AFUA_1G17520 | D-lactate dehydrogenase (cytochrome), putative |  |  | 72 | 73 | 2.684633776 |
|  | Afu1g17530 | AFUA_1G17530 | MFS transporter, putative |  |  | 78 | 73 | 2.255920613 |
| cluster 16 | Afu1g17590 | AFUA_1G17590 | phosphoesterase superfamily protein | 95 | 86 | 76 |  | 3.384709874 |
|  | Afu1g17600 | AFUA_1G17600 | conserved hypothetical protein | 90 |  |  |  | 4.050327116 |
|  | Afu1g17650 | AFUA_1G17650 | short chain dehydrogenase, putative | 92 |  | 57 | 68 | 2.374524585 |
|  | Afu1g17660 | AFUA_1G17660 | histidinol dehydrogenase, putative | 96 |  | 72 | 82 | 6.554405975 |
|  | Afu1g17670 | AFUA_1G17670 | salicylate hydroxylase, putative | 89 |  | 80 | 72 | 8.191306242 |
|  | Afu1g17680 | AFUA_1G17680 | MFS transporter, putative | 95 |  | 84 | 28 | 9.747123902 |
| cluster 17 | Afu2g00120 | AFUA_2G00120 | carboxyvinyl-carboxyphosphonate phosphorylmutase |  |  | 87 | 79 | 2.205479955 |
|  | Afu2g00130 | AFUA_2G00130 | solid-state culture specific protein, putative | 89 |  | 73 | 63 | 3.490967983 |
|  | Afu2g00170 | AFUA_2G00170 | glutathione-dependent formaldehyde dehydrogenase | 95 |  |  |  | 2.171388046 |
|  | Afu2g00210 | AFUA_2G00210 | conserved hypothetical protein | 93 | 68 |  | 50 | 3.397150427 |
|  | Afu2g00240 | AFUA_2G00240 | conserved hypothetical protein | 77 | 67 |  |  | 4.07014347 |
|  | Afu2g00260 | AFUA_2G00260 | phenylacetaldoxime dehydratase family protein, putative | 90 | 60 |  | 51 | 3.927826268 |
| cluster 18 | Afu2g00490 | AFUA_2G00490 | glycosyl hydrolase, family 31 |  |  |  |  | 4.683929957 |
|  | Afu2g00500 | AFUA_2G00500 | conserved hypothetical protein | 91 | 76 | 64 | 66 | 4.489006001 |
|  | Afu2g00510 | AFUA_2G00510 | cellulose-binding GDSL lipase/acylhydrolase, putative | 78 | 56 | 49 | 36 | 6.847938777 |
|  | Afu2g00520 | AFUA_2G00520 | conserved hypothetical protein | 93 | 72 | 69 | 56 | 6.356177609 |
|  | Afu2g00540 | AFUA_2G00540 | carboxyphosphonoenolpyruvate phosphonomutase, putative | 98 | 91 |  | 90 | 2.870431264 |
|  | Afu2g00550 | AFUA_2G00550 | conserved hypothetical protein |  |  |  |  | 7.144773387 |
|  | Afu2g00570 | AFUA_2G00570 | GNAT family acetyltransferase, putative | 94 | 67 |  |  | 2.598188699 |
|  | Afu2g00580 | AFUA_2G00580 | hypothetical protein | 86 | 63 |  |  | 6.071518342 |
|  | Afu2g00620 | AFUA_2G00620 | GDSL-like lipase/acylhydrolase domain protein | 87 | 71 | 82 | 45 | 6.14535801 |
|  | Afu2g00640 | AFUA_2G00640 | beta-N-hexosaminidase, putative | 94 | 73 | 58 | 70 | 4.867464818 |
| cluster 19 | Afu2g00700 | AFUA_2G00700 | hypothetical protein |  |  |  |  | 2.252409381 |
|  | Afu2g00740 | AFUA_2G00740 | conserved hypothetical protein | 78 | 55 |  | 57 | 2.19938168 |
|  | Afu2g00770 | AFUA_2G00770 | salicylate hydroxylase | 95 | 86 | 67 |  | 2.069436608 |
|  | Afu2g00790 | AFUA_2G00790 | conserved hypothetical protein | 99 | 80 | 53 | 68 | 2.422910949 |
|  | Afu2g00820 | AFUA_2G00820 | extracellular GDSL-like lipase/acylhydrolase, putative | 95 | 78 | 46 | 69 | 3.73879739 |
|  | Afu2g00830 | AFUA_2G00830 | short chain dehydrogenase/reductase | 91 |  | 64 | 72 | 3.889617526 |
|  | Afu2g00840 | AFUA_2G00840 | MFS transporter, putative | 95 | 86 | 81 | 85 | 2.251895807 |
|  | Afu2g00870 | AFUA_2G00870 | conserved hypothetical protein | 77 | 86 | 47 | 56 | 3.135652479 |
| cluster 20 | Afu2g01230 | AFUA_2G01230 | dihydrodipicolinate synthetase family protein | 97 | 87 | 82 | 80 | 2.989166003 |
|  | Afu2g01280 | AFUA_2G01280 | D-mandelate dehydrogenase, putative | 99 | 87 | 68 | 80 | 4.719088798 |
|  | Afu2g01300 | AFUA_2G01300 | conserved hypothetical protein | 92 | 71 |  |  | 3.409202859 |
|  | Afu2g01310 | AFUA_2G01310 | EF-hand calcium-binding domain protein, putative | 96 | 66 | 45 | 60 | 3.485752169 |
|  | Afu2g01320 | AFUA_2G01320 | potassium/sodium P-type ATPase, putative | 96 | 82 | 73 | 74 | 4.877203249 |
| cluster 21 | Afu2g03820 | AFUA_2G03820 | carboxyphosphonoenolpyruvate phosphonomutase, putative | 99 | 94 | 81 | 89 | -2.521436271 |
|  | Afu2g03850 | AFUA_2G03850 | conserved hypothetical protein | 89 | 69 | 62 | 60 | -3.039488704 |
|  | Afu2g03900 | AFUA_2G03900 | acetamidase/formamidase family protein | 94 | 76 |  |  | -3.31006273 |
|  | Afu2g03930 | AFUA_2G03930 | small nucleolar ribonucleoprotein complex subunit, putative | 95 | 85 | 66 | 77 | -3.28433353 |
|  | Afu2g03950 | AFUA_2G03950 | serine/threonine protein phosphatase, putative | 99 | 95 | 87 | 91 | -2.317643802 |
|  | Afu2g03980 | AFUA_2G03980 | alpha-1,3-glucanase/mutanase, putative | 97 | 82 | 71 | 81 | -6.804133358 |
|  | Afu2g04000 | AFUA_2G04000 | peptidase (PNG1), putative | 95 | 82 | 70 | 75 | -2.673818583 |
| cluster 22 | Afu2g04200 | AFUA_2G04200 | 4-hydroxyphenylpyruvate dioxygenase, putative | 97 | 90 | 85 | 87 | 2.557545223 |
|  | Afu2g04210 | AFUA_2G04210 | conserved hypothetical protein | 98 | 79 | 61 | 65 | 2.267772832 |
|  | Afu2g04220 | AFUA_2G04220 | homogentisate 1,2-dioxygenase (HmgA), putative | 99 | 94 | 89 | 94 | 2.949934555 |
|  | Afu2g04230 | AFUA_2G04230 | fumarylacetoacetate hydrolase FahA | 98 | 91 | 87 | 88 | 3.153790975 |
|  | Afu2g04240 | AFUA_2G04240 | maleylacetoacetate isomerase MaiA | 97 | 84 | 71 | 75 | 2.723446427 |
|  | Afu2g04260 | AFUA_2G04262 | C6 transcription factor, putative | 84 | 84 | 66 | 75 | 3.005332413 |
| cluster 23 | Afu2g04490 | AFUA_2G04490 | D-3-phosphoglycerate dehydrogenase | 89 |  |  |  | 3.428846125 |
|  | Afu2g04520 | AFUA_2G04520 | Fe-containing alcohol dehydrogenase, putative | 99 | 92 | 91 | 92 | 2.989010664 |
|  | Afu2g04530 | AFUA_2G04530 | hypothetical protein |  |  |  |  | 2.119035324 |
|  | Afu2g04540 | AFUA_2G04540 | conserved hypothetical protein | 97 | 83 | 70 | 58 | 3.968897418 |
|  | Afu2g04570 | AFUA_2G04570 | BNR/Asp-box repeat domain protein | 92 |  | 59 | 69 | 4.356346525 |
|  | Afu2g04590 | AFUA_2G04590 | alcohol dehydrogenase, putative | 98 |  | 76 | 89 | 4.749069631 |
|  | Afu2g04600 | AFUA_2G04600 | C6 transcription factor, putative | 89 |  |  | 47 | 4.089665342 |
| cluster 24 | Afu2g05230 | AFUA_2G05230 | conserved hypothetical protein | 92 | 66 | 49 | 50 | 3.34581508 |
|  | Afu2g05240 | AFUA_2G05240 | conserved hypothetical protein | 94 | 69 | 53 | 43 | 2.34499253 |
|  | Afu2g05260 | AFUA_2G05260 | salicylate hydroxylase, putative | 98 | 86 | 75 | 85 | 4.638959886 |
|  | Afu2g05290 | AFUA_2G05290 | isoflavone reductase family protein | 94 |  | 49 |  | 5.803663432 |
|  | Afu2g05300 | AFUA_2G05300 | hypothetical protein |  |  |  |  | 2.964110045 |
|  | Afu2g05310 | AFUA_2G05310 | C6 transcription factor, putative | 96 | 79 | 68 | 74 | 4.46114187 |
| cluster 25 | Afu2g07750 | AFUA_2G07750 | haloacid dehalogenase, type II | 91 | 68 | 57 | 64 | 3.003253541 |
|  | Afu2g07780 | AFUA_2G07780 | acyl-CoA synthetase, putative | 99 | 83 | 81 | 82 | 3.869190966 |
|  | Afu2g07810 | AFUA_2G07810 | cytosolic hydroxymethyltransferase, putative | 99 | 92 | 85 | 87 | 2.077475893 |
|  | Afu2g07820 | AFUA_2G07820 | MOSC domain protein | 94 | 58 | 55 | 56 | 2.999882607 |
|  | Afu2g07840 | AFUA_2G07840 | competence/damage-inducible protein CinA, putative | 97 |  |  |  | 2.656598013 |
| cluster 26 | Afu2g11740 | AFUA_2G11740 | mitochondrial serine protease Pim1, putative | 98 | 90 | 82 | 85 | -2.21913301 |
|  | Afu2g11790 | AFUA_2G11790 | conserved hypothetical protein | 96 | 82 | 59 | 76 | -2.071288399 |
|  | Afu2g11800 | AFUA_2G11800 | small nuclear ribonucleoprotein (LSM1), putative | 98 | 89 | 51 |  | -2.73951358 |
|  | Afu2g11810 | AFUA_2G11810 | pre-rRNA processing protein Rrp12, putative | 99 | 89 | 81 | 81 | -2.913924906 |
|  | Afu2g11850 | AFUA_2G11850 | 60S ribosomal protein L3 | 99 | 98 | 96 | 96 | -2.053364543 |
|  | Afu2g11870 | AFUA_2G11870 | beta-1,6 glucan synthetase (Kre6), putative | 97 | 85 | 68 | 72 | -2.203267064 |
| cluster 27 | Afu2g12120 | AFUA_2G12120 | conserved hypothetical protein | 92 | 69 | 48 | 57 | -2.188831133 |
|  | Afu2g12150 | AFUA_2G12150 | midasin, putative | 96 | 82 | 69 | 73 | -2.519986849 |
|  | Afu2g12200 | AFUA_2G12200 | cAMP-dependent protein kinase catalytic subunit PkaC1 | 96 | 88 | 86 | 77 | -2.230233973 |
|  | Afu2g12250 | AFUA_2G12250 | DNA replication factor C subunit Rfc5, putative | 100 | 97 | 91 | 93 | -2.75086174 |
|  | Afu2g12260 | AFUA_2G12260 | cytochrome c oxidase assembly protein Cox11, putative | 87 | 81 | 75 | 79 | -3.018231793 |
|  | Afu2g12280 | AFUA_2G12280 | HET domain protein, putative | 81 | 55 |  |  | -3.340709216 |
| cluster 28 | Afu2g14330 | AFUA_2G14330 | conserved hypothetical protein | 94 | 85 | 57 | 71 | 4.247391433 |
|  | Afu2g14350 | AFUA_2G14350 | AT DNA binding protein, putative | 87 | 50 | 35 | 36 | 2.266508416 |
|  | Afu2g14390 | AFUA_2G14390 | methyltransferase, putative | 94 | 73 | 58 | 62 | 3.048956191 |
|  | Afu2g14410 | AFUA_2G14410 | dioxygenase, putative | 94 | 77 | 54 | 65 | 4.509926836 |
|  | Afu2g14430 | AFUA_2G14430 | cytochrome p450, putative | 89 |  | 31 | 32 | 3.559729529 |
|  | Afu2g14450 | AFUA_2G14450 | hypothetical protein | 83 |  |  |  | 6.10760116 |
|  | Afu2g14460 | AFUA_2G14460 | oxidoreductase, short-chain dehydrogenase/reductase family | 95 |  |  | 68 | 2.637360602 |
|  | Afu2g14470 | AFUA_2G14470 | oxidoreductase, FAD-binding, putative | 91 | 66 |  |  | 3.109053324 |
|  | Afu2g14480 | AFUA_2G14480 | oxidoreductase, FAD-binding, putative | 85 |  |  |  | 7.005663035 |
| cluster 29 | Afu2g17240 | AFUA_2G17240 | C2H2 finger domain protein, putative | 81 |  |  |  | 2.225020238 |
|  | Afu2g17280 | AFUA_2G17280 | hypothetical protein | 54 |  |  |  | 4.632676481 |
|  | Afu2g17290 | AFUA_2G17290 | hypothetical protein | 85 |  |  |  | 3.32749851 |
|  | Afu2g17300 | AFUA_2G17300 | glutathione S-transferase | 90 |  |  |  | 4.050307318 |
|  | Afu2g17320 | AFUA_2G17320 | conserved hypothetical protein | 95 | 72 | 58 | 41 | 2.648858808 |
|  | Afu2g17330 | AFUA_2G17330 | extracelular serine carboxypeptidase, putative | 94 | 71 | 59 | 63 | 2.031344825 |
| cluster 30 | Afu2g17520 | AFUA_2G17520 | GNAT family N-acetyltransferase, putative | 95 |  |  |  | 5.658035927 |
|  | Afu2g17560 | AFUA_2G17560 | conidial pigment biosynthesis 1,3,6,8-tetrahydroxynaphthalene reductase Arp2 | 92 | 85 | 52 | 52 | 2.744251116 |
|  | Afu2g17630 | AFUA_2G17630 | conserved hypothetical protein | 94 | 73 |  | 45 | 4.435625483 |
|  | Afu2g17640 | AFUA_2G17640 | conserved hypothetical protein |  |  |  |  | 5.188463414 |
|  | Afu2g17650 | AFUA_2G17650 | DUF907 domain protein | 94 | 78 |  |  | 2.92140402 |
| cluster 31 | Afu3g01030 | AFUA_3G01030 | RTA1 domain protein, putative | 95 | 70 | 57 | 58 | 3.701418332 |
|  | Afu3g01060 | AFUA_3G01060 | hypothetical protein |  |  |  |  | 2.125171059 |
|  | Afu3g01080 | AFUA_3G01080 | conserved hypothetical protein | 88 | 66 |  | 51 | 2.160256738 |
|  | Afu3g01130 | AFUA_3G01130 | cell wall protein, putative | 89 | 52 |  |  | 2.194574835 |
|  | Afu3g01140 | AFUA_3G01140 | hypothetical protein |  |  |  |  | 2.69618562 |
|  | Afu3g01150 | AFUA_3G01150 | GPI anchored cell wall protein, putative | 89 |  |  |  | 2.590258975 |
|  | Afu3g01170 | AFUA_3G01170 | cystathionine beta-lyase | 89 |  |  |  | 2.48728778 |
|  | Afu3g01180 | AFUA_3G01180 | sarcosine oxidase, putative | 95 |  |  | 71 | 4.785222763 |
|  | Afu3g01210 | AFUA_3G01210 | ThiJ/PfpI family protein | 98 | 84 | 68 | 80 | 3.858784396 |
|  | Afu3g01220 | AFUA_3G01220 | aspartic-type endopeptidase, putative | 91 | 61 |  |  | 3.529729469 |
|  | Afu3g01230 | AFUA_3G01230 | MFS sugar transporte, putative |  |  |  |  | 7.167094616 |
|  | Afu3g01240 | AFUA_3G01240 | hypothetical protein | 91 |  |  |  | 6.139460316 |
|  | Afu3g01260 | AFUA_3G01260 | acetyltransferase, GNAT family family | 88 |  |  | 44 | 4.087535021 |
|  | Afu3g01280 | AFUA_3G01280 | alpha/beta hydrolase, putative | 94 | 70 |  |  | 5.206583195 |
|  | Afu3g01320 | AFUA_3G01320 | homocysteine S-methyltransferase, putative | 96 | 73 |  |  | 2.578801539 |
|  | Afu3g01330 | AFUA_3G01330 | class II aldolase/adducin domain protein | 98 | 87 | 81 | 85 | 3.662987333 |
|  | Afu3g01360 | AFUA_3G01360 | siderochrome-iron transporter, putative | 89 |  | 66 | 69 | 4.008902514 |
|  | Afu3g01370 | AFUA_3G01370 | MFS transporter, putative | 97 | 86 | 75 | 79 | 7.201360144 |
|  | Afu3g01400 | AFUA_3G01400 | ABC multidrug transporter, putative | 97 | 84 | 78 | 79 | 3.799397199 |
|  | Afu3g01410 | AFUA_3G01410 | polyketide synthase, putative | 90 | 63 |  | 52 | 3.793806288 |
|  | Afu3g01450 | AFUA_3G01450 | 3-methyl-2-oxobutanoate dehydrogenase, putative | 99 | 93 | 83 | 87 | 2.635628377 |
|  | Afu3g01470 | AFUA_3G01470 | conserved hypothetical protein | 96 |  |  | 45 | 2.060759692 |
|  | Afu3g01480 | AFUA_3G01480 | conserved hypothetical protein | 93 | 76 | 65 | 71 | 2.746959756 |
|  | Afu3g01500 | AFUA_3G01500 | integral membrane protein | 82 |  |  |  | 2.423602309 |
|  | Afu3g01530 | AFUA_3G01530 | phosphatidylglycerol specific phospholipase, putative | 94 | 84 | 63 | 72 | 4.115915989 |
|  | Afu3g01580 | AFUA_3G01580 | GMC oxidoreductase, putative | 96 | 90 | 82 | 83 | 3.826057445 |
|  | Afu3g01610 | AFUA_3G01610 | conserved hypothetical protein | 93 | 63 | 49 |  | 5.398741177 |
|  | Afu3g01620 | AFUA_3G01620 | Ankyrin and HET domain protein | 91 |  |  | 59 | 4.586943203 |
|  | Afu3g01630 | AFUA_3G01630 | RTA1 domain protein, putative | 94 |  |  |  | 3.072948784 |
|  | Afu3g01660 | AFUA_3G01660 | glycosyl hydrolase, family 43, putative | 96 |  | 52 | 73 | 3.730505951 |
|  | Afu3g01670 | AFUA_3G01670 | MFS hexose transporter, putative | 87 | 76 |  |  | 4.350252774 |
|  | Afu3g01700 | AFUA_3G01700 | MFS alpha-glucoside transporter, putative | 97 | 86 | 76 |  | 4.749333946 |
|  | Afu3g01710 | AFUA_3G01710 | TPR domain protein | 88 |  |  |  | 2.539274165 |
|  | Afu3g01730 | AFUA_3G01730 | hypothetical protein |  |  |  |  | 4.114404301 |
| cluster 32 | Afu3g02210 | AFUA_3G02210 | C6 transcription factor, putative | 89 |  |  | 51 | 2.021976641 |
|  | Afu3g02220 | AFUA_3G02220 | DUF427 domain protein | 98 | 83 | 57 | 64 | 3.154062636 |
|  | Afu3g02250 | AFUA_3G02250 | 3-beta hydroxysteroid dehydrogenase/isomerase family protein | 95 |  | 58 | 76 | 2.938298991 |
|  | Afu3g02260 | AFUA_3G02260 | conserved hypothetical protein | 94 | 92 |  |  | 3.631845548 |
|  | Afu3g02280 | AFUA_3G02280 | alpha,alpha-trehalose glucohydrolase TreA/Ath1 | 96 | 83 | 74 | 68 | 3.112103437 |
|  | Afu3g02290 | AFUA_3G02290 | arylsulfatase, putative | 96 | 89 |  |  | 3.402405641 |
|  | Afu3g02300 | AFUA_3G02300 | proline oxidase Put1, putative | 93 | 71 | 64 | 44 | 5.807179486 |
| cluster 33 | Afu3g03200 | AFUA_3G03200 | cysteine hydrolase family protein |  |  |  |  | 3.539736002 |
|  | Afu3g03240 | AFUA_3G03240 | MFS monocarboxylate transporter, putative | 93 | 47 | 44 | 42 | 3.035571574 |
|  | Afu3g03280 | AFUA_3G03280 | FAD binding monooxygenase, putative | 96 | 73 | 59 | 68 | 2.239600082 |
|  | Afu3g03310 | AFUA_3G03310 | RTA1 domain protein | 91 |  |  | 51 | 2.313003581 |
|  | Afu3g03320 | AFUA_3G03320 | MFS monocarboxylate transporter, putative | 96 | 85 |  |  | 2.769701714 |
|  | Afu3g03350 | AFUA_3G03350 | nonribosomal peptide synthase SidE | 95 | 81 |  |  | 4.854128205 |
|  | Afu3g03380 | AFUA_3G03380 | sugar O-acetyltransferase, putative | 95 |  | 75 |  | 3.203190691 |
|  | Afu3g03390 | AFUA_3G03390 | siderophore biosynthesis lipase/esterase, putative | 95 | 76 | 67 | 76 | 3.412522235 |
|  | Afu3g03400 | AFUA_3G03400 | siderophore biosynthesis acetylase AceI, putative | 96 | 85 | 80 | 81 | 6.452066875 |
|  | Afu3g03410 | AFUA_3G03410 | enoyl-CoA hydratase/isomerase family protein | 97 | 85 | 85 | 86 | 7.420572778 |
|  | Afu3g03420 | AFUA_3G03420 | nonribosomal peptide synthase SidD | 94 | 70 | 64 | 71 | 6.143170913 |
|  | Afu3g03430 | AFUA_3G03430 | ABC multidrug transporter SitT | 97 | 85 | 82 | 84 | 5.16141005 |
|  | Afu3g03440 | AFUA_3G03440 | MFS siderophore iron transporter, putative | 96 | 74 | 74 | 75 | 6.10512804 |
|  | Afu3g03450 | AFUA_3G03450 | cytochrome P450 oxidoreductase, putative | 96 |  | 76 | 67 | 3.450141292 |
| cluster 34 | Afu3g03600 | AFUA_3G03600 | carboxylesterase, putative |  |  |  |  | 2.310601524 |
|  | Afu3g03640 | AFUA_3G03640 | MFS siderochrome iron transporter MirB | 93 | 72 | 55 | 59 | 6.454904934 |
|  | Afu3g03650 | AFUA_3G03650 | GNAT family acetyltransferase, putative | 91 | 75 | 58 | 66 | 2.638083203 |
|  | Afu3g03660 | AFUA_3G03660 | siderophore esterase IroE-like, putative | 90 | 75 | 44 | 71 | 3.385422974 |
|  | Afu3g03670 | AFUA_3G03670 | ABC multidrug transporter, putative | 95 | 79 | 66 | 77 | 8.777330855 |
|  | Afu3g03700 | AFUA_3G03700 | MFS sugar transporter, putative | 91 |  |  | 77 | 5.779938316 |
|  | Afu3g03750 | AFUA_3G03750 | hypothetical protein |  |  |  |  | 2.712325812 |
|  | Afu3g03810 | AFUA_3G03810 | hypothetical protein | 81 |  | 44 |  | 7.981874644 |
|  | Afu3g03820 | AFUA_3G03820 | MFS nicotinic acid transporter Tna1, putative | 96 | 90 | 74 | 76 | 6.174113775 |
|  | Afu3g03850 | AFUA_3G03852 | C2H2 type zinc finger domain protein | 98 | 83 |  |  | 5.036546065 |
|  | Afu3g03860 | AFUA_3G03860 | 3-hydroxyacyl-CoA dehydrogenase, putative | 92 | 78 |  |  | 6.942646682 |
|  | Afu3g03870 | AFUA_3G03870 | endo-1,4-beta-glucanase, putative | 90 | 72 | 71 | 67 | 2.688104306 |
| cluster 35 | Afu3g06360 | AFUA_3G06360 | UBX domain protein | 81 | 59 | 43 | 47 | -4.259867059 |
|  | Afu3g06380 | AFUA_3G06380 | exosome-associated family protein | 92 | 75 | 68 | 78 | -2.090223245 |
|  | Afu3g06400 | AFUA_3G06400 | hypothetical protein | 94 | 82 | 62 | 49 | -2.52398494 |
|  | Afu3g06430 | AFUA_3G06430 | GDP/GTP exchange factor Sec2p, putative | 95 | 77 | 68 | 64 | -3.035262902 |
|  | Afu3g06470 | AFUA_3G06470 | DHHC zinc finger membrane protein, putative | 96 | 83 | 70 | 76 | -2.189458843 |
| cluster 36 | Afu3g07380 | AFUA_3G07380 | oligo-1,6-glucosidase | 95 | 86 | 74 | 76 | 2.313466156 |
|  | Afu3g07400 | AFUA_3G07400 | conserved hypothetical protein | 77 | 65 |  |  | 4.094660615 |
|  | Afu3g07410 | AFUA_3G07410 | isoamyl alcohol oxidase | 98 | 79 | 62 | 79 | 3.629784564 |
|  | Afu3g07420 | AFUA_3G07420 | conserved hypothetical protein | 84 | 70 | 58 | 60 | 2.315264133 |
|  | Afu3g07440 | AFUA_3G07440 | DRAP deaminase, putative | 96 |  |  |  | 2.141812797 |
|  | Afu3g07460 | AFUA_3G07460 | NACHT and WD domain protein | 90 |  |  |  | 3.616377877 |
|  | Afu3g07530 | AFUA_3G07530 | pH signal transduction protein PalI, putative | 92 | 74 | 65 | 70 | 2.342168442 |
|  | Afu3g07560 | AFUA_3G07560 | enoyl-CoA hydratase/isomerase family protein | 92 |  | 60 | 63 | 2.043493826 |
|  | Afu3g07610 | AFUA_3G07610 | conserved hypothetical protein | 84 | 48 | 55 | 38 | 3.900317546 |
| cluster 37 | Afu3g11650 | AFUA_3G11650 | hypothetical protein | 91 | 66 | 54 | 65 | 3.976815517 |
|  | Afu3g11720 | AFUA_3G11720 | conserved hypothetical protein | 81 | 39 |  | 36 | 3.903918153 |
|  | Afu3g11770 | AFUA_3G11770 | conserved hypothetical protein | 83 | 71 | 66 | 89 | 4.043829479 |
|  | Afu3g11780 | AFUA_3G11780 | beta-N-acetylglucosaminidase, putative | 91 | 76 | 50 | 64 | 2.90086232 |
|  | Afu3g11790 | AFUA_3G11790 | galactose-proton symport, putative | 98 | 85 | 76 | 74 | 4.636016964 |
| cluster 38 | Afu4g00240 | AFUA_4G00240 | amino acid transporter, putative | 96 |  |  |  | 2.498223306 |
|  | Afu4g00280 | AFUA_4G00280 | conserved hypothetical protein | 91 | 62 | 55 | 57 | 3.872109753 |
|  | Afu4g00330 | AFUA_4G00330 | hypothetical protein | 89 |  |  | 53 | 4.911442725 |
|  | Afu4g00370 | AFUA_4G00370 | general amidase, putative | 92 | 75 | 63 | 69 | 3.492088827 |
|  | Afu4g00390 | AFUA_4G00390 | glycosyl hydrolase, putative | 94 | 73 | 50 | 61 | 3.177447564 |
|  | Afu4g00400 | AFUA_4G00400 | O-methyltransferase, putative | 94 |  |  | 34 | 4.807879247 |
|  | Afu4g00430 | AFUA_4G00430 | isoamyl alcohol oxidase, putative | 95 |  | 41 |  | 3.256706045 |
| cluster 39 | Afu4g00750 | AFUA_4G00750 | conserved hypothetical protein | 84 |  | 64 |  | 4.045850721 |
|  | Afu4g00770 | AFUA_4G00770 | conserved hypothetical protein | 84 | 49 | 32 | 36 | 2.434011232 |
|  | Afu4g00800 | AFUA_4G00800 | MFS monosaccharide transporter, putative | 97 |  | 78 |  | 4.864801899 |
|  | Afu4g00860 | AFUA_4G00860 | cell surface protein, putative |  |  |  |  | 3.043095214 |
|  | Afu4g00870 | AFUA_4G00870 | antigenic cell wall galactomannoprotein, putative | 97 | 73 | 59 | 67 | 4.316438927 |
| cluster 40 | Afu4g00980 | AFUA_4G00980 | conserved hypothetical protein | 83 | 55 |  | 46 | 3.403134045 |
|  | Afu4g00990 | AFUA_4G00990 | MFS quinate transporter, putative | 97 | 90 | 79 | 83 | 4.362039147 |
|  | Afu4g01000 | AFUA_4G01000 | MFS transporter, putative | 97 | 87 | 80 | 83 | 4.434349911 |
|  | Afu4g01020 | AFUA_4G01020 | sensor histidine kinase/response regulator, putative | 87 | 53 | 40 | 43 | 3.111460169 |
|  | Afu4g01030 | AFUA_4G01030 | conserved hypothetical protein | 89 | 73 |  | 57 | 3.098783004 |
|  | Afu4g01040 | AFUA_4G01040 | short chain dehydrogenase, putative | 90 |  | 59 | 69 | 3.634087715 |
|  | Afu4g01060 | AFUA_4G01060 | conserved hypothetical protein | 92 | 71 |  |  | 3.38284477 |
|  | Afu4g01070 | AFUA_4G01070 | acid phosphatase, putative | 97 |  | 67 | 63 | 4.576610248 |
| cluster 41 | Afu4g01470 | AFUA_4G01470 | C6 finger domain protein, putative | 92 | 64 |  |  | 2.996666339 |
|  | Afu4g01480 | AFUA_4G01480 | MFS transporter, putative |  |  | 30 | 75 | 5.702928667 |
|  | Afu4g01500 | AFUA_4G01500 | lignostilbene dioxygenase, putative |  |  | 81 | 84 | 2.37983811 |
|  | Afu4g01510 | AFUA_4G01510 | C6 transcription factor, putative |  |  | 29 | 85 | 5.056532962 |
|  | Afu4g01520 | AFUA_4G01520 | gentisate 1,2-dioxygenase, putative |  |  |  | 81 | 2.418913944 |
|  | Afu4g01550 | AFUA_4G01550 | aldehyde dehydrogenase, putative |  | 57 |  | 78 | 4.842127462 |
|  | Afu4g01580 | AFUA_4G01580 | ankyrin repeat protein | 92 | 69 | 57 | 55 | 3.975194618 |
| cluster 42 | Afu4g08810 | AFUA_4G08810 | DUF757 domain protein | 99 | 82 | 82 | 78 | -2.653509322 |
|  | Afu4g08860 | AFUA_4G08860 | hypothetical protein |  |  |  |  | -2.45933288 |
|  | Afu4g08890 | AFUA_4G08890 | aldo-keto reductase family protein, putative | 97 | 78 |  |  | -2.930056249 |
|  | Afu4g08900 | AFUA_4G08900 | CDK-activating kinase assembly factor MAT1 | 98 | 86 | 72 | 84 | -2.072031932 |
|  | Afu4g08930 | AFUA_4G08930 | nucleolar GTPase, putative | 99 | 87 | 84 | 81 | -2.26193325 |
|  | Afu4g08960 | AFUA_4G08960 | GPI anchored cell wall protein, putative | 89 |  | 66 | 81 | -2.81091791 |
|  | Afu4g08970 | AFUA_4G08970 | PAP2 domain protein | 94 | 82 | 65 | 70 | -2.332427803 |
| cluster 43 | Afu4g09300 | AFUA_4G09300 | conserved hypothetical protein | 90 | 61 |  | 60 | 5.352318203 |
|  | Afu4g09310 | AFUA_4G09310 | conserved hypothetical protein | 93 |  |  |  | 3.147089686 |
|  | Afu4g09320 | AFUA_4G09320 | extracellular dipeptidyl-peptidase Dpp4 | 98 | 83 | 72 | 72 | 5.642295426 |
|  | Afu4g09330 | AFUA_4G09330 | conserved hypothetical protein | 90 | 66 |  | 66 | 2.197744238 |
|  | Afu4g09340 | AFUA_4G09340 | conserved hypothetical protein | 88 | 82 |  | 59 | 2.648390234 |
| cluster 44 | Afu4g13900 | AFUA_4G13900 | MFS transporter, putative | 93 |  |  | 77 | 3.203943269 |
|  | Afu4g13950 | AFUA_4G13950 | GNAT family acetyltransferase, putative | 90 | 62 |  |  | 3.512211488 |
|  | Afu4g13970 | AFUA_4G13970 | conserved hypothetical protein |  |  |  |  | 4.808413414 |
|  | Afu4g13990 | AFUA_4G13990 | conserved hypothetical protein | 95 | 82 |  | 67 | 4.714335586 |
|  | Afu4g14040 | AFUA_4G14040 | Hsp70 family protein | 82 |  | 55 |  | 3.253732247 |
|  | Afu4g14070 | AFUA_4G14070 | glycosyl transferase, putative | 94 |  | 61 | 62 | 6.098633033 |
|  | Afu4g14080 | AFUA_4G14080 | spherulin 4-like cell surface protein, putative | 93 |  | 48 |  | 5.323675342 |
|  | Afu4g14090 | AFUA_4G14090 | UDP-glucose 4-epimerase | 91 |  |  |  | 4.058353553 |
|  | Afu4g14120 | AFUA_4G14120 | cutinase, putative | 93 |  | 72 | 83 | 3.87044661 |
|  | Afu4g14130 | AFUA_4G14130 | ABC multidrug transporter, putative | 95 |  |  | 78 | 3.949585677 |
|  | Afu4g14150 | AFUA_4G14150 | Diacylglycerol acyltransferase family | 95 |  | 65 | 85 | 4.16628511 |
|  | Afu4g14170 | AFUA_4G14170 | conserved hypothetical protein | 91 | 80 |  |  | 2.384532328 |
|  | Afu4g14180 | AFUA_4G14180 | conserved hypothetical protein | 91 | 65 |  |  | 3.200953154 |
|  | Afu4g14200 | AFUA_4G14200 | conserved hypothetical protein | 93 | 75 |  |  | 3.376500993 |
| cluster 45 | Afu5g00480 | AFUA_5G00480 | exoinulinase InuD | 94 |  | 62 |  | 5.264025067 |
|  | Afu5g00490 | AFUA_5G00490 | hypothetical protein |  |  |  |  | 6.232906476 |
|  | Afu5g00500 | AFUA_5G00500 | MFS alpha-glucoside transporter, putative | 83 |  | 51 |  | 2.722422011 |
|  | Afu5g00510 | AFUA_5G00510 | conserved hypothetical protein | 94 |  | 76 |  | 4.289834049 |
|  | Afu5g00520 | AFUA_5G00520 | C6 transcription factor, putative | 90 |  |  |  | 3.463378172 |
|  | Afu5g00540 | AFUA_5G00540 | FacC-like extracellular signaling protein, putative | 95 |  |  |  | 5.009338142 |
|  | Afu5g00550 | AFUA_5G00550 | endoglycoceramidase, putative | 91 |  |  | 83 | 4.699216169 |
|  | Afu5g00580 | AFUA_5G00580 | conserved hypothetical protein | 89 |  |  |  | 2.464346253 |
| cluster 46 | Afu5g00670 | AFUA_5G00670 | beta-galactosidase, putative | 93 |  | 57 | 74 | 2.229215652 |
|  | Afu5g00680 | AFUA_5G00680 | CRAL/TRIO domain protein | 85 |  | 44 | 51 | 3.026191168 |
|  | Afu5g00700 | AFUA_5G00700 | hypothetical protein | 84 |  | 46 | 31 | 4.406923217 |
|  | Afu5g00710 | AFUA_5G00710 | GABA permease, putative | 96 | 82 | 78 | 66 | 6.28253003 |
|  | Afu5g00720 | AFUA_5G00720 | GNAT family acetyltransferase, putative | 94 | 71 | 63 | 29 | 5.149691907 |
|  | Afu5g00770 | AFUA_5G00770 | integral membrane protein, putative | 90 | 71 | 52 | 58 | 2.167608255 |
|  | Afu5g00790 | AFUA_5G00790 | ABC multidrug transporter, putative | 95 | 81 | 77 | 77 | 2.279282734 |
|  | Afu5g00810 | AFUA_5G00810 | conserved hypothetical protein | 92 | 62 |  |  | 2.137806622 |
|  | Afu5g00870 | AFUA_5G00870 | conserved hypothetical protein | 93 |  |  |  | 2.6539403 |
|  | Afu5g00900 | AFUA_5G00900 | G-protein signalling regulator, putative | 82 |  |  | 49 | 3.693584889 |
|  | Afu5g00920 | AFUA_5G00920 | transesterase (LovD), putative | 92 | 74 |  |  | 2.317790152 |
| cluster 47 | Afu5g01240 | AFUA_5G01240 | general amidase, putative | 86 |  |  | 63 | 2.918185492 |
|  | Afu5g01250 | AFUA_5G01250 | oxidoreductase, putative | 94 | 75 | 57 | 59 | 4.783912897 |
|  | Afu5g01270 | AFUA_5G01272 | C6 transcription factor, putative | 94 | 67 | 41 |  | 2.853038379 |
|  | Afu5g01290 | AFUA_5G01290 | zinc-binding oxidoreductase, putative | 96 | 80 | 58 | 68 | 4.154815357 |
|  | Afu5g01300 | AFUA_5G01300 | integral membrane protein | 96 | 74 |  |  | 2.073825813 |
|  | Afu5g01310 | AFUA_5G01310 | RTA1 domain protein, putative | 95 |  | 72 | 75 | 2.202659313 |
|  | Afu5g01340 | AFUA_5G01340 | lysophospholipase Plb2 | 70 |  |  |  | 3.973819527 |
|  | Afu5g01380 | AFUA_5G01380 | conserved hypothetical protein | 87 | 67 | 57 | 58 | 2.710547134 |
|  | Afu5g01410 | AFUA_5G01410 | hypothetical protein |  |  |  |  | 3.713513339 |
|  | Afu5g01420 | AFUA_5G01420 | conserved hypothetical protein | 97 | 73 | 65 |  | 3.324346036 |
| cluster 48 | Afu5g01600 | AFUA_5G01600 | PHP domain protein | 93 |  |  |  | 3.929432295 |
|  | Afu5g01610 | AFUA_5G01610 | hypothetical protein |  |  |  |  | 3.181960409 |
|  | Afu5g01620 | AFUA_5G01620 | extracellular proline-rich protein | 90 |  |  | 49 | 2.625127751 |
|  | Afu5g01630 | AFUA_5G01630 | MFS multidrug transporter, putative | 96 | 84 |  | 78 | 2.484083983 |
|  | Afu5g01650 | AFUA_5G01650 | bZIP transcription factor JlbA/IDI-4 | 91 | 48 | 72 | 39 | 2.167476669 |
|  | Afu5g01680 | AFUA_5G01680 | MFS transporter, putative | 96 |  |  | 78 | 3.179151968 |
| cluster 49 | Afu5g07520 | AFUA_5G07520 | cytochrome P450 oxidoreductase, putative | 96 | 45 | 75 | 74 | 6.324828513 |
|  | Afu5g07530 | AFUA_5G07530 | conserved hypothetical protein | 93 | 56 |  |  | 6.301741725 |
|  | Afu5g07580 | AFUA_5G07580 | methylmalonyl-CoA decarboxylase, alpha subunit, putative | 97 |  | 81 |  | 5.556850794 |
|  | Afu5g07630 | AFUA_5G07630 | ubiE/COQ5 methyltransferase, putative | 59 |  |  |  | 3.22509814 |
|  | Afu5g07640 | AFUA_5G07640 | RNA 3'-terminal phosphate cyclase, putative | 90 | 57 | 47 | 51 | 3.059335021 |
|  | Afu5g07650 | AFUA_5G07650 | F-box domain and ankyrin repeat protein | 87 |  |  |  | 3.192382551 |
|  | Afu5g07660 | AFUA_5G07660 | conserved hypothetical protein | 89 | 59 |  |  | 4.666792813 |
|  | Afu5g07670 | AFUA_5G07670 | SH3 domain signalling protein | 95 | 72 | 52 | 63 | 2.506063144 |
| cluster 50 | Afu5g10090 | AFUA_5G10090 | 3-demethylubiquinone-9 3-methyltransferase, putative | 59 |  |  |  | 9.469958352 |
|  | Afu5g10130 | AFUA_5G10130 | bZIP transcription factor, putative | 80 |  |  | 44 | 2.494044757 |
|  | Afu5g10150 | AFUA_5G10150 | hypothetical protein |  |  |  |  | 2.252464475 |
|  | Afu5g10160 | AFUA_5G10160 | NmrA-like family protein | 93 |  |  | 65 | 3.129720828 |
|  | Afu5g10180 | AFUA_5G10180 | salicylate hydroxylase, putative | 93 | 77 | 52 | 58 | 2.909037876 |
|  | Afu5g10190 | AFUA_5G10190 | conserved hypothetical protein | 96 | 73 |  | 65 | 3.451492246 |
|  | Afu5g10250 | AFUA_5G10250 | conserved hypothetical protein | 87 | 60 | 67 | 66 | 4.949763519 |
|  | Afu5g10290 | AFUA_5G10290 | fructose-bisphosphate aldolase, putative | 99 | 87 | 85 | 88 | 3.740114247 |
|  | Afu5g10300 | AFUA_5G10300 | hypothetical protein | 76 |  | 41 |  | 2.792953879 |
|  | Afu5g10350 | AFUA_5G10350 | conserved hypothetical protein |  |  |  |  | 3.1051708 |
| cluster 51 | Afu5g12620 | AFUA_5G12620 | CorA family metal ion transporter, putative | 92 | 77 | 65 | 69 | -3.199133794 |
|  | Afu5g12660 | AFUA_5G12660 | phosphotidylinositol kinase Tel1, putative | 95 | 78 | 61 | 67 | -3.547230463 |
|  | Afu5g12710 | AFUA_5G12710 | TPR domain protein | 84 | 63 | 56 |  | -5.485837901 |
|  | Afu5g12740 | AFUA_5G12740 | MFS multidrug transporter, putative |  |  | 33 |  | -2.342342652 |
|  | Afu5g12750 | AFUA_5G12750 | hypothetical protein | 85 |  |  |  | -2.497723391 |
| cluster 52 | Afu5g13640 | AFUA_5G13640 | thioredoxin, putative | 96 | 69 | 60 | 49 | 2.186078193 |
|  | Afu5g13650 | AFUA_5G13650 | conserved hypothetical protein | 97 | 83 | 64 | 55 | 2.674300737 |
|  | Afu5g13690 | AFUA_5G13690 | DUF74 domain protein | 94 |  |  |  | 2.99860483 |
|  | Afu5g13730 | AFUA_5G13730 | NlpC/P60-like cell-wall peptidase, putative | 89 | 81 |  |  | 4.392796521 |
|  | Afu5g13770 | AFUA_5G13770 | hypothetical protein |  |  |  |  | 2.232815469 |
|  | Afu5g13800 | AFUA_5G13800 | transcriptional regulator, putative | 97 | 77 | 70 | 54 | 4.30404914 |
|  | Afu5g13810 | AFUA_5G13810 | transulfuration enzyme family protein, putative | 97 | 85 | 77 | 77 | 2.420602741 |
| cluster 53 | Afu5g14420 | AFUA_5G14420 | hypothetical protein | 95 |  | 61 | 72 | 2.662337466 |
|  | Afu5g14500 | AFUA_5G14500 | MFS transporter, putative | 92 |  | 76 | 68 | 2.237832005 |
|  | Afu5g14540 | AFUA_5G14540 | MFS monosaccharide transporter, putative | 97 |  | 74 | 41 | 3.313150507 |
|  | Afu5g14550 | AFUA_5G14550 | beta-galactosidase, putative | 93 |  | 62 | 40 | 3.165725818 |
|  | Afu5g14570 | AFUA_5G14570 | conserved hypothetical protein | 85 |  |  |  | 3.565071431 |
|  | Afu5g14620 | AFUA_5G14620 | hypothetical protein | 78 |  |  |  | 5.990361982 |
|  | Afu5g14650 | AFUA_5G14650 | RING finger protein | 89 | 70 |  | 47 | 3.937759939 |
|  | Afu5g14660 | AFUA_5G14660 | GABA permease, putative | 86 | 69 | 74 | 68 | 2.913645773 |
|  | Afu5g14670 | AFUA_5G14670 | conserved hypothetical protein | 96 | 68 | 41 | 59 | 3.378679288 |
| cluster 54 | Afu6g00120 | AFUA_6G00120 | C6 transcription factor, putative |  |  |  | 48 | 4.452331416 |
|  | Afu6g00130 | AFUA_6G00130 | MFS transporter, putative |  |  | 64 | 63 | 5.092558942 |
|  | Afu6g00140 | AFUA_6G00140 | hypothetical protein |  |  |  |  | 3.712223012 |
|  | Afu6g00160 | AFUA_6G00160 | Ser/Thr protein phosphatase family protein | 88 |  |  |  | 6.03232491 |
|  | Afu6g00190 | AFUA_6G00190 | conserved hypothetical protein | 81 | 54 | 61 | 74 | 2.231858819 |
| cluster 55 | Afu6g00600 | AFUA_6G00600 | conserved hypothetical protein | 89 | 68 | 55 | 72 | 2.895425252 |
|  | Afu6g00620 | AFUA_6G00620 | GPI anchored hypothetical protein | 82 | 55 |  |  | 3.841135358 |
|  | Afu6g00630 | AFUA_6G00630 | MFS transporter, putative | 92 | 80 | 44 | 74 | 4.579668794 |
|  | Afu6g00640 | AFUA_6G00640 | integral membrane protein | 87 | 69 |  | 52 | 3.37076586 |
|  | Afu6g00670 | AFUA_6G00670 | extracellular serine-rich protein | 86 | 69 | 62 | 72 | 2.851689488 |
|  | Afu6g00680 | AFUA_6G00680 | conserved hypothetical protein | 96 | 82 | 59 | 61 | 3.175655826 |
|  | Afu6g00690 | AFUA_6G00690 | conserved hypothetical protein |  |  |  |  | 2.9597392 |
|  | Afu6g00710 | AFUA_6G00710 | MFS transporter, putative | 96 | 83 | 70 | 87 | 4.660339586 |
|  | Afu6g00750 | AFUA_6G00750 | pyruvate decarboxylase, putative | 97 | 87 | 68 | 81 | 3.676179822 |
| cluster 56 | Afu6g01790 | AFUA_6G01790 | conserved hypothetical protein | 95 | 86 | 55 |  | 2.530618 |
|  | Afu6g01820 | AFUA_6G01820 | MFS transporter, putative | 94 | 86 | 67 | 84 | 2.64960727 |
|  | Afu6g01830 | AFUA_6G01830 | O-methyltransferase, putative | 92 | 76 | 53 | 56 | 4.942509074 |
|  | Afu6g01860 | AFUA_6G01860 | MFS lactose permease, putative | 95 | 85 | 78 | 40 | 3.081148942 |
|  | Afu6g01870 | AFUA_6G01870 | conserved hypothetical protein | 96 | 78 |  |  | 3.099163624 |
|  | Afu6g01900 | AFUA_6G01900 | flavin-binding monooxygenase-like protein | 92 | 76 | 57 | 68 | 3.204288422 |
|  | Afu6g01930 | AFUA_6G01930 | MFS transporter Seo1, putative | 95 | 88 | 77 | 78 | 2.971877781 |
| cluster 57 | Afu6g03140 | AFUA_6G03140 | oligopeptide transporter, putative | 94 |  | 48 | 80 | 2.477453437 |
|  | Afu6g03150 | AFUA_6G03150 | conserved hypothetical protein | 88 | 72 |  |  | 4.936094037 |
|  | Afu6g03160 | AFUA_6G03160 | NACHT domain protein | 87 |  |  |  | 5.197974408 |
|  | Afu6g03180 | AFUA_6G03180 | hypothetical protein | 92 |  |  | 57 | 4.615753024 |
|  | Afu6g03190 | AFUA_6G03190 | hypothetical protein | 95 |  |  | 69 | 5.271828082 |
|  | Afu6g03200 | AFUA_6G03200 | solute symporter family transporter | 96 | 83 | 47 |  | 4.595958817 |
|  | Afu6g03210 | AFUA_6G03210 | conidiation-specific protein (Con-10), putative | 88 | 84 | 84 | 79 | 4.761186886 |
|  | Afu6g03220 | AFUA_6G03220 | hypothetical protein |  |  |  |  | 2.163863051 |
|  | Afu6g03240 | AFUA_6G03240 | HET-s/LopB domain protein | 95 | 74 |  |  | 3.879392753 |
|  | Afu6g03250 | AFUA_6G03252 | protein kinase, putative | 91 | 64 |  |  | 2.470825448 |
| cluster 58 | Afu6g03320 | AFUA_6G03320 | MFS multidrug transporter, putative | 95 |  | 81 | 66 | 2.163683831 |
|  | Afu6g03330 | AFUA_6G03330 | conserved hypothetical protein | 85 |  | 58 | 29 | 4.038789218 |
|  | Afu6g03340 | AFUA_6G03340 | conserved hypothetical protein | 82 | 54 |  |  | 5.138939861 |
|  | Afu6g03350 | AFUA_6G03350 | GNAT family N-acetyltransferase, putative | 93 | 74 |  |  | 5.576748015 |
|  | Afu6g03370 | AFUA_6G03370 | short-chain dehydrogenase/reductase, putative | 93 |  |  |  | 2.737279327 |
|  | Afu6g03430 | AFUA_6G03430 | C6 finger domain protein, putative | 88 |  |  |  | 5.0650387 |
|  | Afu6g03440 | AFUA_6G03440 | fructosyl amino acid oxidase, putative | 95 |  |  | 46 | 5.915371237 |
|  | Afu6g03450 | AFUA_6G03450 | N-methyltransferase, putative | 93 |  |  | 48 | 2.4312916 |
|  | Afu6g03460 | AFUA_6G03460 | conserved hypothetical protein | 92 |  |  | 68 | 5.192317207 |
|  | Afu6g03470 | AFUA_6G03470 | ABC multidrug transporter, putative | 95 |  |  |  | 4.379100246 |
|  | Afu6g03480 | AFUA_6G03480 | NRPS-like enzyme, putative | 87 |  |  | 57 | 3.096718237 |
|  | Afu6g03490 | AFUA_6G03490 | phenol 2-monooxygenase, putative | 91 |  |  | 52 | 6.76054364 |
| cluster 59 | Afu6g04270 | AFUA_6G04270 | MFS sugar transporter, putative | 98 | 89 | 65 | 82 | -3.045780493 |
|  | Afu6g04300 | AFUA_6G04300 | conserved hypothetical protein | 99 | 85 | 81 | 71 | -2.096583787 |
|  | Afu6g04340 | AFUA_6G04340 | hypothetical protein | 92 |  |  |  | -4.569038636 |
|  | Afu6g04350 | AFUA_6G04350 | conserved hypothetical protein | 97 | 71 | 54 | 57 | -2.470672432 |
|  | Afu6g04380 | AFUA_6G04380 | molecular chaperone (ABC1), putative | 96 | 82 | 63 | 70 | -3.412983011 |
|  | Afu6g04390 | AFUA_6G04390 | histone ubiquitinationc protein (Bre1), putative | 97 | 88 | 78 | 78 | -2.426328681 |
| cluster 60 | Afu6g09610 | AFUA_6G09610 | nonribosomal peptide synthase, putative | 76 |  |  |  | 2.718608946 |
|  | Afu6g09630 | AFUA_6G09630 | C6 finger domain protein GliZ | 79 |  |  |  | 4.690653018 |
|  | Afu6g09640 | AFUA_6G09640 | aminotransferase GliI | 92 | 37 |  | 36 | 5.189362579 |
|  | Afu6g09650 | AFUA_6G09650 | membrane dipeptidase GliJ | 95 | 51 | 46 | 51 | 5.580823512 |
|  | Afu6g09660 | AFUA_6G09660 | nonribosomal peptide synthase GliP | 95 | 29 |  | 29 | 5.882285514 |
|  | Afu6g09670 | AFUA_6G09670 | cytochrome P450 oxidoreductase GliC | 96 | 38 |  | 39 | 5.670002518 |
|  | Afu6g09680 | AFUA_6G09680 | O-methyltransferase GliM | 96 | 45 |  | 44 | 8.341131382 |
|  | Afu6g09690 | AFUA_6G09690 | glutathione S-transferase GliG | 98 | 45 |  | 46 | 6.929057623 |
|  | Afu6g09700 | AFUA_6G09700 | gliotoxin biosynthesis protein GliK | 92 | 36 |  | 34 | 5.689811099 |
|  | Afu6g09710 | AFUA_6G09710 | MFS gliotoxin efflux transporter GliA | 96 |  |  | 51 | 4.456308908 |
|  | Afu6g09720 | AFUA_6G09720 | methyltransferase GliN | 95 |  |  |  | 8.226337111 |
|  | Afu6g09730 | AFUA_6G09730 | cytochrome P450 oxidoreductase GliF | 97 |  |  |  | 2.159268201 |
| cluster 61 | Afu6g11850 | AFUA_6G11850 | conserved hypothetical protein | 92 | 80 |  |  | 6.150258592 |
|  | Afu6g11910 | AFUA_6G11910 | glycosyl hydrolase family 3, putative | 94 |  | 78 |  | 2.266930252 |
|  | Afu6g11920 | AFUA_6G11920 | MFS alpha-glucoside transporter, putative | 91 |  | 84 |  | 4.692639711 |
|  | Afu6g11930 | AFUA_6G11930 | conserved hypothetical protein | 82 |  | 69 |  | 4.021360538 |
|  | Afu6g11950 | AFUA_6G11950 | hypothetical protein |  |  |  |  | 2.764165084 |
|  | Afu6g11990 | AFUA_6G11990 | hypothetical protein |  |  |  |  | 4.369839472 |
|  | Afu6g12000 | AFUA_6G12000 | hypothetical protein | 80 |  |  |  | 5.728909377 |
| cluster 62 | Afu6g13740 | AFUA_6G13740 | conserved hypothetical protein | 95 | 73 |  | 49 | 4.732190795 |
|  | Afu6g13750 | AFUA_6G13750 | ferric-chelate reductase, putative | 92 | 65 | 55 | 60 | 3.971797308 |
|  | Afu6g13790 | AFUA_6G13790 | flavin-binding monooxygenase, putative | 94 | 77 | 64 | 59 | 2.721944246 |
|  | Afu6g13810 | AFUA_6G13810 | hypothetical protein | 82 |  |  | 44 | 3.260365793 |
|  | Afu6g13830 | AFUA_6G13830 | oxidoreductase, short chain dehydrogenase/reductase family | 98 | 87 | 77 | 83 | 4.202229935 |
|  | Afu6g13840 | AFUA_6G13840 | conserved hypothetical protein | 94 | 63 | 57 | 72 | 2.57041876 |
|  | Afu6g13850 | AFUA_6G13850 | GTPase activating protein (Evi5), putative | 95 | 83 | 76 | 74 | 2.935997693 |
| Cluster 63 | Afu7g00110 | AFUA_7G00110 | secretory lipase, putative | 92 |  | 57 | 60 | 4.325402085 |
|  | Afu7g00120 | AFUA_7G00120 | metallo-beta-lactamase domain protein | 96 |  | 63 | 45 | 3.867942574 |
|  | Afu7g00130 | AFUA_7G00130 | C6 transcription factor, putative | 87 |  |  |  | 6.301774788 |
|  | Afu7g00150 | AFUA_7G00150 | FAD-dependent monooxygenase, putative | 94 |  | 60 |  | 3.321228691 |
|  | Afu7g00160 | AFUA_7G00160 | polyketide synthase, putative | 96 |  | 66 | 46 | 5.959945705 |
|  | Afu7g00170 | AFUA_7G00170 | dimethylallyl tryptophan synthase GliD1 | 97 |  |  |  | 3.820033083 |
|  | Afu7g00180 | AFUA_7G00180 | NAD dependent epimerase/dehydratase, putative | 93 |  |  |  | 3.72144793 |
|  | Afu7g00200 | AFUA_7G00200 | conserved hypothetical protein | 95 |  |  | 56 | 2.258183067 |
| cluster 64 | Afu7g00370 | AFUA_7G00370 | conserved hypothetical protein | 87 | 74 |  | 53 | 2.940454264 |
|  | Afu7g00380 | AFUA_7G00380 | GNAT family acetyltransferase, putative | 90 | 69 |  |  | 3.856463842 |
|  | Afu7g00420 | AFUA_7G00420 | hypothetical protein | 91 |  | 38 | 36 | 2.415556946 |
|  | Afu7g00440 | AFUA_7G00440 | GABA permease, putative | 92 |  | 67 | 66 | 2.394013499 |
|  | Afu7g00460 | AFUA_7G00460 | conserved hypothetical protein | 87 |  | 57 |  | 2.512210651 |
| cluster 65 | Afu7g00770 | AFUA_7G00770 | C6 transcription factor, putative | 81 | 67 | 39 | 70 | 2.09203924 |
|  | Afu7g00780 | AFUA_7G00780 | MFS monocarboxylate transporter, putative | 94 | 85 | 76 | 68 | 5.059434305 |
|  | Afu7g00790 | AFUA_7G00790 | hypothetical protein |  |  |  |  | 2.388696848 |
|  | Afu7g00800 | AFUA_7G00800 | acid phosphatase, putative | 93 | 81 | 71 | 75 | 2.026791648 |
|  | Afu7g00820 | AFUA_7G00820 | hypothetical protein | 87 |  |  |  | 2.922440636 |
|  | Afu7g00830 | AFUA_7G00830 | alpha/beta hydrolase, putative | 90 | 66 |  |  | 2.041648909 |
|  | Afu7g00850 | AFUA_7G00850 | GNAT family acetyltransferase, putative | 92 | 72 | 56 | 54 | 3.521534547 |
|  | Afu7g00870 | AFUA_7G00870 | RNA exonuclease, putative | 88 | 59 | 39 | 55 | 4.025127504 |
|  | Afu7g00880 | AFUA_7G00880 | hypothetical protein | 46 |  |  |  | 2.159992685 |
| cluster 66 | Afu7g00970 | AFUA_7G00970 | GPI anchored serine-threonine rich protein | 96 | 48 | 63 | 34 | 5.85922836 |
|  | Afu7g00990 | AFUA_7G00990 | transcriptional activator of ethanol catabolism AlcS | 82 | 65 | 69 | 66 | 2.044935224 |
|  | Afu7g01000 | AFUA_7G01000 | aldehyde dehydrogenase, putative | 96 | 86 |  |  | 3.977209593 |
|  | Afu7g01010 | AFUA_7G01010 | alcohol dehydrogenase, putative | 96 | 92 | 86 | 90 | 3.654818324 |
|  | Afu7g01020 | AFUA_7G01020 | hypothetical protein |  |  |  |  | 5.078819257 |
|  | Afu7g01030 | AFUA_7G01030 | calcium transporting ATPase (Pmc1), putative | 94 |  | 70 |  | 5.714089664 |
|  | Afu7g01040 | AFUA_7G01040 | cytidine deaminase, putative | 89 |  |  | 66 | 2.900649597 |
|  | Afu7g01050 | AFUA_7G01050 | salicylate hydroxylase, putative | 84 | 59 | 54 | 37 | 9.03889508 |
|  | Afu7g01060 | AFUA_7G01060 | cysteine-rich secreted protein | 93 | 75 | 50 | 64 | 2.428341869 |
|  | Afu7g01070 | AFUA_7G01070 | endo-1,4-beta-mannosidase | 92 | 81 | 69 | 72 | 3.941711298 |
|  | Afu7g01090 | AFUA_7G01090 | proline permease PrnB | 96 | 88 | 79 | 81 | 5.706903085 |
|  | Afu7g01100 | AFUA_7G01100 | conserved hypothetical protein | 80 | 65 | 42 |  | 2.007279656 |
| cluster 67 | Afu7g04910 | AFUA_7G04910 | phosphatidylglycerol specific phospholipase C, putative | 96 | 86 | 86 | 66 | -2.399263838 |
|  | Afu7g04950 | AFUA_7G04950 | lipase, putative |  |  |  | 46 | -5.342730922 |
|  | Afu7g05000 | AFUA_7G05000 | conserved hypothetical protein | 80 |  |  |  | -2.169819226 |
|  | Afu7g05030 | AFUA_7G05030 | pectin lyase B | 97 |  |  | 83 | -2.188756473 |
|  | Afu7g05070 | AFUA_7G05070 | FAD dependent oxidoreductase, putative | 90 | 66 | 60 | 63 | -2.77654697 |
| cluster 68 | Afu7g06120 | AFUA_7G06120 | transmembrane transporter, putative | 96 | 86 |  | 64 | 2.831226159 |
|  | Afu7g06130 | AFUA_7G06130 | plasma membrane protein Pth11-like, putative | 96 | 79 |  |  | 5.151990278 |
|  | Afu7g06140 | AFUA_7G06140 | beta-D-glucoside glucohydrolase | 97 |  | 72 |  | 7.164853239 |
|  | Afu7g06160 | AFUA_7G06160 | hypothetical protein | 94 |  |  |  | 4.76288789 |
|  | Afu7g06170 | AFUA_7G06170 | thiamin pyrophosphokinase-related protein | 90 |  |  |  | 4.027532163 |
|  | Afu7g06180 | AFUA_7G06180 | hypothetical protein | 86 |  |  |  | 3.752578792 |
| cluster 69 | Afu7g06750 | AFUA_7G06750 | phosphoglycerate mutase family protein, putative | 87 | 69 | 70 | 80 | 2.229437718 |
|  | Afu7g06760 | AFUA_7G06760 | CRAL/TRIO domain protein | 90 |  |  |  | 3.357878842 |
|  | Afu7g06800 | AFUA_7G06800 | glutamyl-tRNA(Gln) amidotransferase, subunit A | 87 |  | 41 | 50 | 3.033689135 |
|  | Afu7g06810 | AFUA_7G06810 | L-amino acid oxidase LaoA | 95 |  | 80 | 73 | 7.728249039 |
|  | Afu7g06850 | AFUA_7G06850 | phosphoserine phosphatase, putative | 85 |  |  | 57 | 5.280902897 |
|  | Afu7g06910 | AFUA_7G06910 | hypothetical protein |  |  |  |  | 2.407292882 |
|  | Afu7g06920 | AFUA_7G06920 | NmrA family transcriptional regulator, putative |  |  | 69 | 55 | 5.181303111 |
| cluster 70 | Afu7g07010 | AFUA_7G07010 | hypothetical protein |  |  |  |  | -2.373756533 |
|  | Afu7g07040 | AFUA_7G07040 | hypothetical protein |  |  |  |  | -4.190921839 |
|  | Afu7g07060 | AFUA_7G07060 | hypothetical protein |  |  |  |  | -2.781983116 |
|  | Afu7g07140 | AFUA_7G07140 | hypothetical protein |  |  |  |  | -3.340161027 |
|  | Afu7g08250 | AFUA_7G08250 | C6 finger domain protein, putative |  |  |  |  | -3.171463299 |
| cluster 71 | Afu8g00100 | AFUA_8G00100 | aspartate-tRNA ligase, putative |  |  |  |  | 3.61950154 |
|  | Afu8g00110 | AFUA_8G00110 | oxidoreductase, 2OG-Fe(II) oxygenase family, putative |  |  |  |  | 3.262305967 |
|  | Afu8g00120 | AFUA_8G00120 | hypothetical protein |  | 57 |  | 52 | 2.45392198 |
|  | Afu8g00130 | AFUA_8G00130 | hypothetical protein |  | 58 |  | 44 | 3.145020629 |
|  | Afu8g00150 | AFUA_8G00150 | conserved hypothetical protein |  |  |  |  | 4.310435853 |
|  | Afu8g00160 | AFUA_8G00160 | conserved hypothetical protein |  |  |  |  | 2.132452537 |
| cluster 72 | Afu8g00240 | AFUA_8G00240 | cytochrome P450 monooxygenase, putative | 93 |  |  |  | 4.619857417 |
|  | Afu8g00280 | AFUA_8G00280 | short-chain dehydrogenase, putative | 92 | 79 | 57 |  | 2.542006175 |
|  | Afu8g00360 | AFUA_8G00360 | NlpC/P60-like cell-wall peptidase, putative | 94 |  |  |  | 5.6821133 |
|  | Afu8g00370 | AFUA_8G00370 | polyketide synthase, putative | 84 |  |  |  | 5.149378699 |
|  | Afu8g00390 | AFUA_8G00390 | O-methyltransferase, putative | 96 |  | 41 | 49 | 4.448825474 |
|  | Afu8g00420 | AFUA_8G00420 | C6 finger transcription factor, putative | 85 |  |  |  | 4.824826376 |
|  | Afu8g00430 | AFUA_8G00430 | conserved hypothetical protein |  |  |  |  | 4.777625406 |
|  | Afu8g00440 | AFUA_8G00440 | steroid monooxygenase, putative |  |  |  |  | 3.650200403 |
|  | Afu8g00480 | AFUA_8G00480 | phytanoyl-CoA dioxygenase family protein | 94 |  |  |  | 4.994227345 |
|  | Afu8g00490 | AFUA_8G00490 | PKS-like enzyme, putative |  |  |  |  | 4.744191198 |
|  | Afu8g00500 | AFUA_8G00500 | acetate-CoA ligase, putative |  |  |  |  | 7.309815039 |
|  | Afu8g00520 | AFUA_8G00520 | integral membrane protein |  |  |  |  | 8.686607449 |
|  | Afu8g00530 | AFUA_8G00530 | alpha/beta superfamily hydrolase |  | 93 |  | 41 | 2.441133251 |
|  | Afu8g00540 | AFUA_8G00540 | hybrid PKS-NRPS PsoA |  | 85 |  |  | 6.150315997 |
|  | Afu8g00550 | AFUA_8G00550 | methyltransferase SirN-like, putative |  | 93 |  |  | 6.619716458 |
|  | Afu8g00560 | AFUA_8G00560 | cytochrome P450 oxidoreductase, putative |  | 90 |  |  | 6.533699741 |
|  | Afu8g00570 | AFUA_8G00570 | alpha/beta hydrolase, putative |  | 91 |  |  | 4.46285962 |
|  | Afu8g00580 | AFUA_8G00580 | glutathione S-transferase, putative | 93 | 88 |  |  | 7.766976909 |
|  | Afu8g00600 | AFUA_8G00600 | NAD dependent epimerase/dehydratase family protein | 94 |  | 73 | 80 | 3.117442854 |
|  | Afu8g00610 | AFUA_8G00610 | cell surface protein Mas1, putative | 93 |  |  | 61 | 2.229933644 |
|  | Afu8g00620 | AFUA_8G00620 | dimethylallyl tryptophan synthase, putative | 94 |  |  |  | 2.957643214 |
| cluster 73 | Afu8g00900 | AFUA_8G00900 | spherulin 4-like cell surface protein, putative | 91 | 58 |  | 63 | 3.902936734 |
|  | Afu8g00910 | AFUA_8G00910 | conserved hypothetical protein | 93 | 72 | 55 | 64 | 3.452619366 |
|  | Afu8g00940 | AFUA_8G00940 | MFS multidrug transporter, putative | 90 |  | 61 | 66 | 2.217857839 |
|  | Afu8g00960 | AFUA_8G00962 | cytochrome P450, putative | 92 | 83 | 79 |  | 2.020698497 |
|  | Afu8g00980 | AFUA_8G00980 | conserved hypothetical protein | 87 | 60 | 63 |  | 2.088233023 |
|  | Afu8g01030 | AFUA_8G01030 | conserved hypothetical protein | 85 |  |  |  | 3.733105568 |
|  | Afu8g01050 | AFUA_8G01050 | lipase/esterase, putative | 91 |  | 63 | 64 | 4.659742223 |
|  | Afu8g01080 | AFUA_8G01080 | conserved hypothetical protein | 91 |  |  |  | 2.177663592 |
|  | Afu8g01090 | AFUA_8G01090 | thioredoxin, putative | 75 |  |  | 61 | 3.4116913 |
|  | Afu8g01100 | AFUA_8G01100 | Melibiase subfamily, putative | 94 | 76 | 67 | 74 | 2.044703972 |
|  | Afu8g01130 | AFUA_8G01130 | alpha-galactosidase C, putative | 95 | 84 | 72 | 73 | 4.628176816 |
|  | Afu8g01160 | AFUA_8G01160 | tartrate dehydrogenase, putative | 97 | 88 | 84 | 87 | 3.824264728 |
| cluster 74 | Afu8g01580 | AFUA_8G01580 | aminotransferase, classes I and II family | 85 |  |  | 71 | 4.149667794 |
|  | Afu8g01590 | AFUA_8G01590 | hypothetical protein |  |  | 78 | 86 | 3.81451801 |
|  | Afu8g01640 | AFUA_8G01640 | NRPS-like enzyme, putative | 89 |  | 40 | 41 | 3.639002051 |
|  | Afu8g01650 | AFUA_8G01650 | hypothetical protein |  |  |  |  | 6.297404902 |
|  | Afu8g01670 | AFUA_8G01670 | bifunctional catalase-peroxidase Cat2 | 97 | 86 | 72 | 83 | 2.33904235 |
| cluster 75 | Afu8g02550 | AFUA_8G02550 | MFS peptide transporter, putative | 93 | 81 | 69 | 70 | 3.865598746 |
|  | Afu8g02560 | AFUA_8G02560 | glyceraldehyde-3-phosphate dehydrogenase, putative | 91 | 84 | 77 | 80 | 4.534340334 |
|  | Afu8g02610 | AFUA_8G02610 | cytochrome P450 monooxygenase, putative | 98 |  | 72 | 80 | 4.108686173 |
|  | Afu8g02620 | AFUA_8G02620 | CobW domain protein | 85 | 74 | 66 | 63 | 4.077518315 |
|  | Afu8g02630 | AFUA_8G02630 | extracellular exo-polygalacturonase, putative | 91 | 61 | 73 | 79 | 2.837323716 |
| cluster 76 | Afu8g05010 | AFUA_8G05010 | C2H2 finger domain protein, putative | 95 | 78 | 62 | 60 | 2.968370144 |
|  | Afu8g05030 | AFUA_8G05030 | conserved hypothetical protein | 97 | 88 | 64 | 70 | 2.636278848 |
|  | Afu8g05040 | AFUA_8G05040 | dihydrodipicolinate synthetase family protein | 98 | 84 | 79 | 79 | 3.069190646 |
|  | Afu8g05050 | AFUA_8G05050 | hypothetical protein |  |  |  |  | 2.017026615 |
|  | Afu8g05080 | AFUA_8G05080 | hypothetical protein |  |  |  |  | 2.84533871 |
| Cluster 77 | Afu8g07240 | AFUA_8G07240 | MFS maltose permease, putative | 97 | 90 | 77 | 83 | 2.242519337 |
|  | Afu8g07250 | AFUA_8G07250 | pectin methylesterase family protein | 95 |  |  |  | 2.207169421 |
|  | Afu8g07260 | AFUA_8G07260 | conserved hypothetical protein | 88 | 54 |  | 62 | 3.865866395 |
|  | Afu8g07300 | AFUA_8G07300 | alpha/beta hydrolase, putative | 91 |  |  | 66 | 2.86708433 |
|  | Afu8g07320 | AFUA_8G07320 | nucleoside-diphosphate-sugar epimerase, putative | 85 | 65 | 49 |  | 6.765290657 |
