# Supplementary material for: Sub-Telomere Directed Gene Expression during Initiation of Invasive Aspergillosis
Source: PLoS Pathog. 2008 Sep 12;4(9):e1000154. doi: 10.1371/journal.ppat.1000154 (PMC2526178; doi:10.1371/journal.ppat.1000154)
Supplement: Table S9 — Oligo sequences for RT-PCR (0.04 MB DOC) [file ppat.1000154.s012.doc]

**Table S9** Oligonucleotides used for QPCR

| Oligo name | Oligonucleotide sequence |
| --- | --- |
| Afu3g03080F | ATACTTGGCGCATCGTCTGG |
| Afu3g03080R | ACGGCCACGTTGAGAATGAA |
| Afu2g07770F | TACGATGTCGTTCGCATGCT |
| Afu2g07770R | TCACAGGATGACGCACTTGAT |
| Afu3g12060F | GTGTTGAAGAGGCCCTCAAG |
| Afu3g12060R | GGGCCAGATATCGTTAGCAAT |
| Afu1g12310F | CTATGCCCGCAAGCATTTCA |
| Afu1g12310R | TCTTGGAGGACATCGATCCAT |
| Afu1g16890F | CCAGATGATGTGCCCGTGAA |
| Afu1g16890R | CGGCTGGAATAGATTCCTTC |
| Afu2g07910F | TTTGGGTTCTATGCTGGGAT |
| Afu2g07910R | GCTTCTGCCTCTCCTGAAGTA |
| Afu2g06210F | AGTTCGGCCGGCTTTGGTA |
| Afu2g06210R | TGAATACAAAGGGGAGGCAA |
| Afu1g15970F | GGATTGATTGCAATCCCAGG |
| Afu1g15970R | TAATGACCGACCATCGATTG |
| Afu2g05360F | CGGATTTCGATTCACGAAGC |
| Afu2g05360R | TGGTATACATGCTCTCCGTGT |
| Afu2g14990F | TCGACCTCATGTACTCGAAG |
| Afu2g14990R | CCTTCCTCCTCTGGTTCCATA |
| Afu6g09610F | ATGGGCCATGCTCGAGATCA |
| Afu6g09610R | GCGAGACCTTCAAGGCATT |
| Afu6g09620F | CGACGATAAGTTCGCGCCGT |
| Afu6g09620R | AAAATCTGCCGGTCCCTGAAA |
| Afu6g09630F | TCTTTGAACCCTATGATCCG |
| Afu6g09630R | AGTAGGTTGCACAATCGAGCC |
